# Manganese-Based Polyoxometalate Nanozyme-Metformin Co-functionalized Hydrogel Promotes Diabetic Wound Regeneration by Enhancing Phagocyte Efferocytosis

**DOI:** 10.34133/research.0964

**Published:** 2025-11-27

**Authors:** Linfeng Li, Jing Zhang, Caiping Yan, Yongjie Wen, Lifu Jia, Xiyang Tang, Yuan Yong, Ke Jiang, Hanfeng Yang, Lu Chen, Yuling Li

**Affiliations:** ^1^ Department of Orthopedics, Laboratory of Biological Tissue Engineering and Digital Medicine, Affiliated Hospital of North Sichuan Medical College, Nanchong, Sichuan 637000, PR China.; ^2^ Institute of Nanomedicine Innovation and Translational Research, Affiliated Hospital of North Sichuan Medical College, Nanchong, Sichuan 637000, PR China.; ^3^ Department of Gastroenterology, Affiliated Hospital of North Sichuan Medical College, Nanchong, Sichuan 637000, PR China.; ^4^ Biotechnology Innovation Drug Application and Transformation Key Laboratory of Sichuan Province, North Sichuan Medical College, Nanchong, Sichuan 637000, PR China.; ^5^ School of Chemistry and Environment, Southwest Minzu University, Chengdu, Sichuan 610041, PR China.

## Abstract

Efferocytosis, mediated by macrophages and dendritic cells (DCs), clears apoptotic cells to maintain tissue homeostasis, suppress inflammation, and promote repair. In diabetic wounds, hyperglycemia disrupts their efferocytic function, impeding healing progression. Therefore, restoring the efferocytic capacity of DCs and macrophages and establishing a dynamic “cyclic efferocytosis” between these 2 cell types are critical for diabetic wound regeneration. DCs are key drivers of cyclic efferocytosis because of their tissue-resident properties and glycogenolysis-driven energy supply. We postulated that targeted modulation of DCs was a promising strategy to establish this cycle. We developed GPP-M@L hydrogel, coencapsulating metformin (Met) and DC-targeted MnPOM nanozyme liposomes (Lipo-Dcpep@MnPOM). Met improved the high-glucose microenvironment. Lipo-Dcpep@MnPOM restored DC efferocytosis by enhancing mitochondrial adenosine triphosphate production. Treated DCs activated macrophage phosphatidylinositol 3-kinase/Akt signaling via paracrine effects, reinstating macrophage efferocytosis and promoting M2 polarization. Macrophages reciprocally enhanced DC efferocytosis via Rap1 and mitochondrial oxidative phosphorylation (OXPHOS), establishing a self-sustaining “cyclic efferocytosis” loop. This process further promoted regeneration by regulating endothelial cells and fibroblasts. Efficacy was confirmed in vitro and in diabetic rat models. In conclusion, by establishing a dynamic cyclic efferocytosis process using GPP-M@L, this study, grounded in the regulatory framework of “hyperglycemia-impaired immune homeostasis–efferocytosis cycle”, provides a novel therapeutic paradigm for healing diabetic wounds and other immune homeostasis-related diseases.

## Introduction

The equilibrium between cellular apoptosis and clearance of damaged cells is critical for tissue homeostasis [1]. Every day, billions of cells undergo apoptosis and are removed by phagocytes such as dendritic cells (DCs) and macrophages through a process called efferocytosis [2,3]. Macrophage efferocytosis is a vital component of tissue repair under physiological conditions. Through this mechanism, macrophages rapidly clear apoptotic neutrophils and prevent the release of inflammatory mediators and subsequent tissue damage [4]. Efferocytosis also accelerates healing by driving reprogramming of the macrophages from a proinflammatory M1 phenotype to an anti-inflammatory M2 phenotype [5]. Current research and therapeutic strategies related to efferocytosis have disproportionately focused on the macrophages. Therefore, the complementary role of DCs in efferocytosis is not well characterized [6]. This knowledge gap impedes comprehensive understanding of efferocytosis and hinders the development of effective regulatory approaches with clinical relevance. DCs play a pivotal role in the activation of adaptive immunity and serve as core effectors in tissue efferocytosis with distinct immunoregulatory functions. Their ability to recognize and clear apoptotic cells is well established under normal physiological conditions [7,8]. DCs express diverse phagocytic and pattern-recognition receptors, which play a critical role in the regulation of innate and adaptive immunity as well as maintenance of tissue homeostasis. DCs are primarily recognized for their role in T cell activation via costimulatory molecules such as CD80, CD86, and major histocompatibility complex class II (MHC-II). DC efferocytosis significantly modulates immunity by suppressing autoimmune responses against self-antigens and releasing factors such as CD86/CXCL7, which induce anti-inflammatory mediators such as prostaglandin E_2_ (PGE_2_) and interleukin-10 (IL-10) post-phagocytosis [9,10]. While the efferocytosis capability of DCs is recognized, research has predominantly centered on their antigen presentation and adaptive immune functions. However, the specific molecular mechanisms underlying DC efferocytosis and its association with inflammation resolution have not been sufficiently investigated.

Although the roles of DCs and macrophages in efferocytosis are well established, the precise details of their molecular crosstalk and functional cooperation are not fully understood. Furthermore, current models are inadequate in capturing their interdependent efferocytic networks [11,12]. We hypothesize that these cells form a dynamic “efferocytic circuit” through coordinated signaling and functional synergy. This intercellular communication reciprocally modulates their activation states, efferocytic efficacy, and immunoregulatory functions. This closed-loop system leads to optimal clearance of apoptotic cells, restoration of immune homeostasis, and well-orchestrated tissue repair. Consequently, targeting this efferocytic circuit represents a promising therapeutic strategy to modulate pathological microenvironments, restore tissue immune homeostasis, and promote healing.

Diabetes mellitus (DM) is a metabolic disorder characterized by chronic hyperglycemia. Globally, adult cases of DM are projected to reach 643 million by 2030 and 783 million by 2045 [13]. Chronic nonhealing diabetic wounds represent a major clinical challenge globally, with recurrence rates of >40% after healing in 4% to 10% of patients and accounting for 50% to 70% of nontraumatic lower-limb amputations [14]. These wounds represent a significant public health burden by substantially increasing healthcare costs. They also trigger sepsis through recurrent infections, potentially leading to multi-organ failure and mortality [15]. Chronic nonhealing diabetic wounds represent a vicious cycle of immune dyshomeostasis, hyperglycemic pathology, and disrupted efferocytic circuitry. The hyperglycemic microenvironment induces excessive apoptosis in functional cells (fibroblasts and endothelial cells) and impairs phagocytic efficacy of the DCs and macrophages. This leads to exacerbation of apoptosis and immune imbalance to perpetuate the vicious cycle. Clinically, this is manifested by sustained inflammation, increased risk of infection, and impaired tissue regeneration, thereby prolonging the chronic inflammatory phase [16–18]. Based on prior research, we hypothesize that DCs represent the “engines” that restore efferocytic circuit. Their ability to sense tissue damage early is critical in initiating efferocytic cycles during wound healing through glycogenolysis-driven energy process [19,20]. However, DC-mediated efferocytosis is energetically demanding. In the diabetic wounds, hyperglycemia disrupts electron transport in the mitochondrial respiratory chain (MRC), thereby inducing oxidative stress and impairing adenosine triphosphate (ATP) production [1,6,21]. ATP depletion causes delayed or failed circuit initiation and directly compromises DC efferocytic capacity. This exacerbates accumulation of apoptotic cells and immune dyshomeostasis, thereby perpetuating a vicious cycle of immune imbalance in the wound, impaired efferocytosis, accumulation of apoptotic cells, and aggravated immune imbalance. Therefore, restoration of mitochondrial ATP synthesis in the DCs represents a promising therapeutic strategy to break this vicious cycle, reinvigorate DC–macrophage efferocytic synergy, and promote wound regeneration.

Nanozymes are nanomaterials mimicking natural enzymes that modulate oxidative stress and immune responses [22]. Molybdenum-based polyoxometalate (POM) nanoclusters show immense potential in the treatment of oxidative stress-related disorders by mimicking superoxide dismutase (SOD) and catalase (CAT) activities to scavenge various reactive oxygen species (ROS) [23]. Nanozyme complexes generated by assembling POM structures with metal ions further enhance their bioactivity [24]. Mitochondrial MnSOD is located in the mitochondrial matrix, where it catalyzes the dismutation of superoxide radicals (O₂^−^) into oxygen and hydrogen peroxide via metal redox cycling at the active site, thereby preventing mitochondrial damage [25]. Manganese-doped POM (MnPOM) stabilizes electron transport and restores mitochondrial function by mimicking both MnSOD and CAT activities. Gelatin methacrylate (GelMA) serves as a drug delivery platform because of its excellent biocompatibility, biodegradability, and rapid gelation [26]. However, GelMA has limited mechanical strength. Functionalized GelMA/PVA/PCA (GPP) hydrogels developed by modifying GelMA with polyvinyl alcohol (PVA) and protocatechuic acid (PCA) have enhanced mechanical properties because of amide bond formation, hydrogen bonding, and physical interpenetration between GelMA and PVA [27]. Functionalized GPP hydrogels demonstrate significant potential for treating refractory diabetic wounds by enabling controlled release of therapeutics (drugs/nanozymes) while adhering to the wound beds through topographical adhesion and hydrogen bonding.

**Fig. 1.**
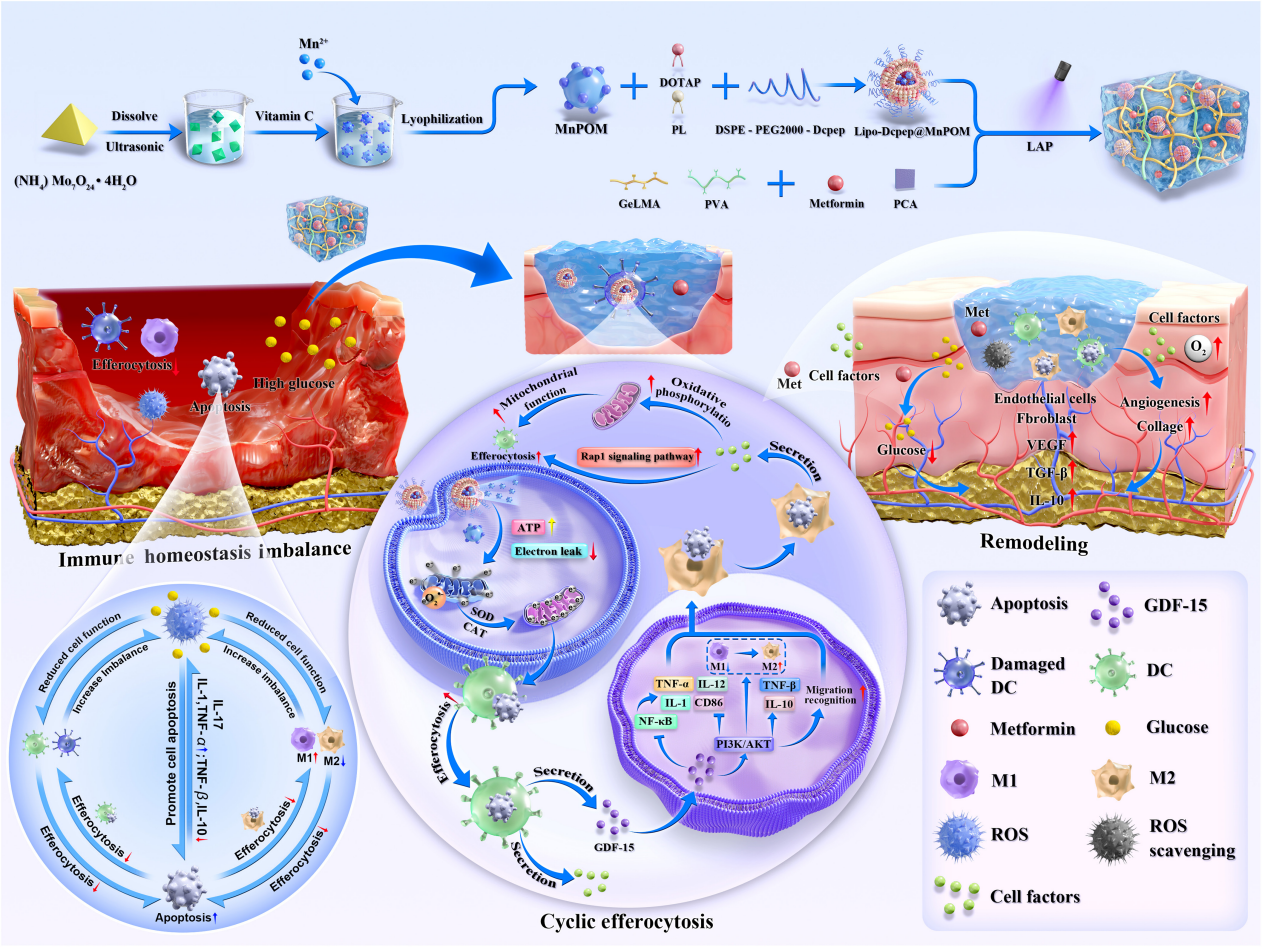
Schematic illustration of manganese-based POM nanozyme-Met co-functionalized hydrogel regulates phagocyte efferocytosis to promote diabetic wound regeneration.

In this study, we engineered an efferocytosis regulator by integrating manganese-doped POM (MnPOM) nanozymes and metformin (Met) to restore DC–macrophage efferocytic circulation and promote diabetic wound healing. Based on a previously published protocol [23], MnPOM with SOD/CAT activities was synthesized via single-step reduction. Then, MnPOM-loaded liposomes (Lipo-Dcpep@MnPOM) were prepared by thin-film hydration [28], with DC-targeting peptides (Dcpep) enabling efficient DC-specific delivery. The efferocytosis regulator GPP-M@L was fabricated by coencapsulating Lipo-Dcpep@MnPOM and Met in the GPP hydrogel. The released Met accelerates glucose consumption in insulin-independent tissues, enhances insulin sensitivity, reduces glucose levels in the wound, and alleviates phagocyte efferocytic burden, thereby improving the hyperglycemic microenvironment [29–31]. The characterization of GPP-M@L was based on in vitro experiments. Conditioned medium was used to determine the mechanism by which GPP-M@L regulated DC–macrophage efferocytic circulation. Topical application of GPP-M@L in the diabetic wound model rats was used to evaluate its in vivo efficacy and mechanisms. Our data show that this regulator breaks the pathological cycle of immune dyshomeostasis, impaired efferocytosis, accumulation of apoptotic cells, and exacerbated immune imbalance. It establishes a restorative DC–macrophage efferocytic circuit, regenerates the wound microenvironment, and significantly accelerates healing (Fig. 1).

## Results and Discussion

### Fabrication and functional characterization of the efferocytosis regulator GPP-M@L

**Fig. 2.**
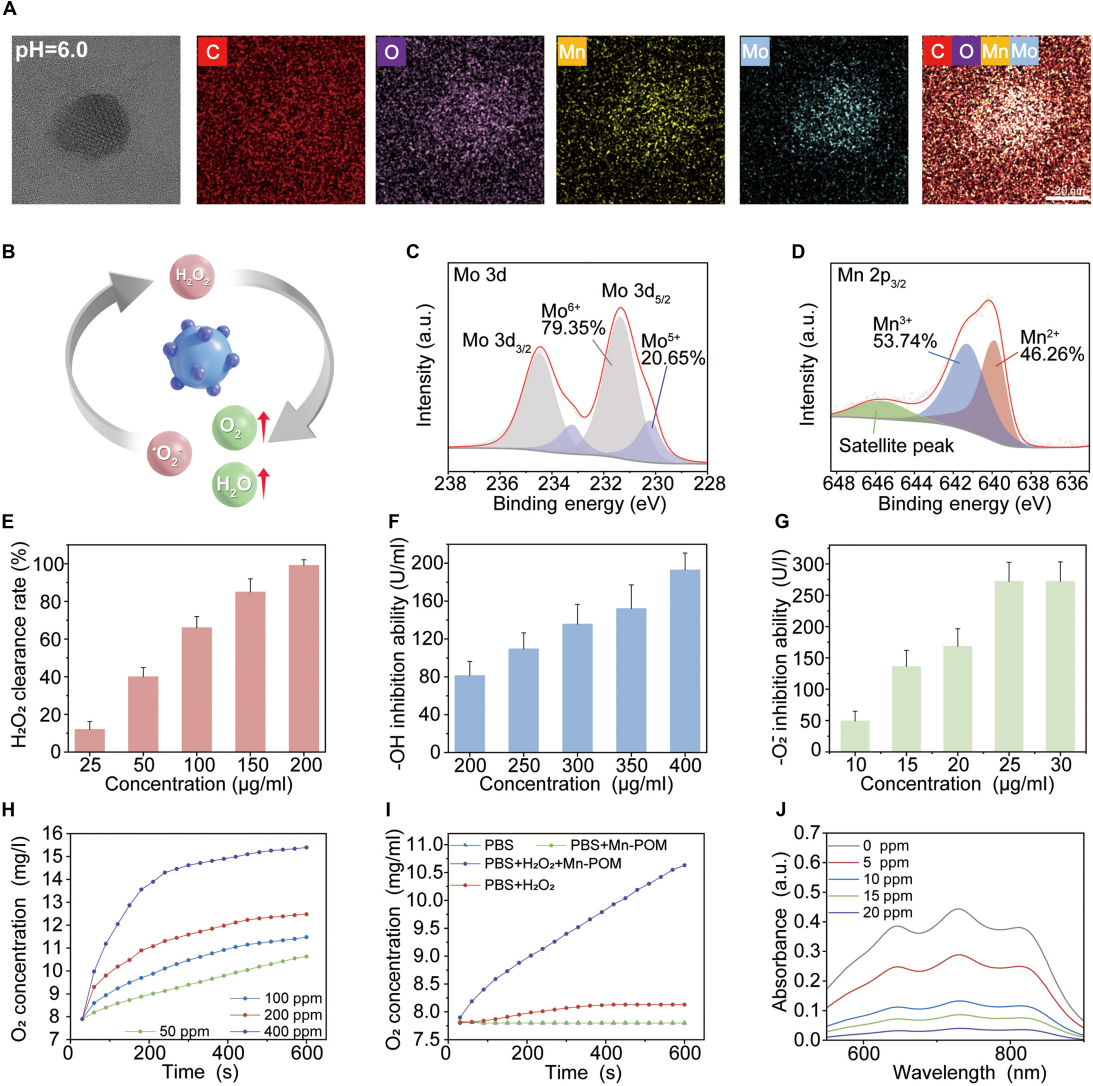
Preparation and characterization of MnPOM. (A) Elemental map of MnPOM. Scale bar, 20 nm. (B) Schematic illustration of MnPOM degradation of O_2_^−^ and H_2_O_2_. (C) Valence band spectrum of Mo 3d in MnPOM. (D) Valence band spectrum of Mn 2p in MnPOM. (E) H_2_O_2_ scavenging efficiency at different MnPOM concentrations. (F) Hydroxyl radical scavenging ability of MnPOM at different concentrations. (G) Superoxide anion scavenging ability of MnPOM at varying concentrations. (H) Oxygen generation curves of MnPOM at different concentrations. (I) Oxygen generation curves for various experimental groups. (J) UV spectra of ABTS scavenging activity at different MnPOM concentrations. All values are presented as mean ± SD; *P < 0.05, **P < 0.01; *P* ≥ 0.05 is considered nonsignificant (NS).

We developed an efferocytosis regulator (GPP-M@L) for enhancing the efficacy of diabetic wound repair by restoring the mitochondrial function of DCs, thereby reinvigorating phagocyte efferocytic circulation and reestablishing immune homeostasis. MnPOM was synthesized via a single-step reduction method to mimic mitochondrial MnSOD and CAT activities. MnPOM efficiently converted MRC-derived ROS into water and oxygen (Fig. 2B). Transmission electron microscopy (TEM) results demonstrated homogeneous dispersion of 4-nm MnPOM at pH 7.4. Acid-induced protonation at pH 6.0 promoted hydrogen bonding and self-assembly into larger nanoparticles (Fig. 2A and Fig. S1), thereby enhancing ROS scavenging in a diabetic wound environment [23,32]. X-ray photoelectron spectroscopy (XPS) valence analysis results confirmed that the elemental states with Mo 3d peaks at 233.15/230.1 eV (Mo^5+^) and 234.5/231.35 eV (Mo^6+^) and Mn 2p peaks at 641.3 eV (Mn^3+^) and 639.0 eV (Mn^2+^) (Fig. 2C and D and Fig. S1). Electron spin resonance (ESR) results (Fig. S2) showed enhanced oxygen vacancy signals at *g* = 2.00393 in MnPOM compared to the undoped POM. Combined TEM/XPS/ESR analyses confirmed successful incorporation of Mn^2+^ doping ions into POM vacancies. ROS scavenging relies on valence transitions. ROS binding triggers redox reactions wherein Mn^2+^/Mo^5+^ ions donate electrons (oxidation) and Mn^3+^/Mo^6+^ ions accept electrons (reduction), thereby enabling continuous ROS clearance through electron transfer cycles. The efficacy of H₂O₂ clearance [KI-starch spectrophotometry/ultraviolet (UV); Figs. S3 and S4] increased in a dose-dependent manner (Fig. 2E). The O_2_ production rate (dissolved oxygen monitoring; Fig. 2H and Fig. S5) peaked at 50 parts per million (ppm) and showed positive concentration dependence and negative pH dependence (Fig. 2I). The scavenging efficiency increased in a dose-dependent manner against multiple ROS (superoxide/[2,2'-azinobis-(3-ethylbenzthiazoline-6-sulphonate)] (ABTS)/hydroxyl radicals) (Fig. 2F and J and Figs. S6 and S7). MnPOM demonstrated dose-dependent scavenging of hydroxyl radicals and SOD-mimetic activity based on quantitative assays, and achieved 272 U/ml SOD activity at a concentration of 30 μg/ml (Fig. 2G). Collectively, these data show that MnPOM exhibits potent SOD/CAT-mimetic activities and functions as a nanozyme for restoring DC mitochondrial function.

**Fig. 3.**
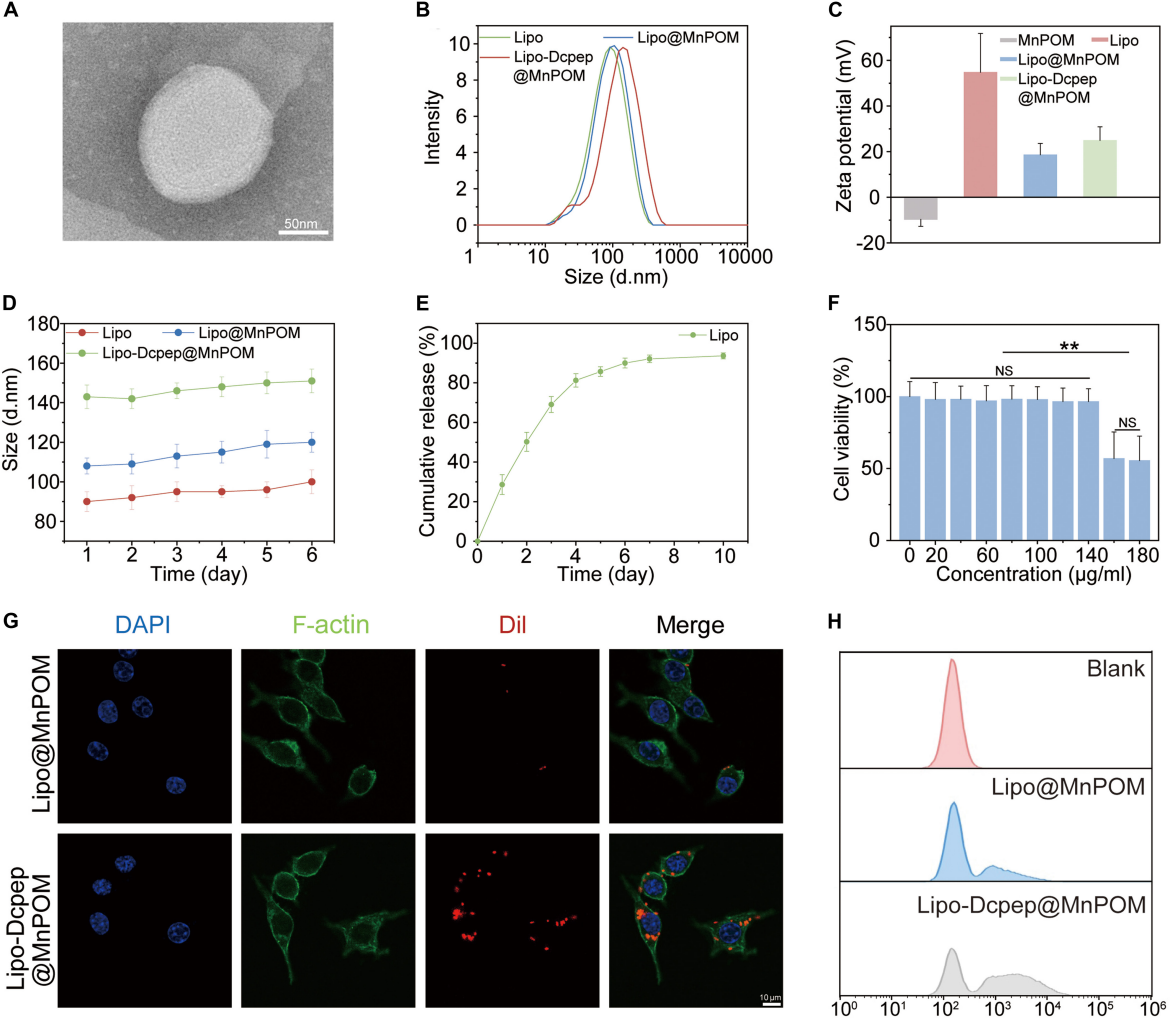
Preparation and characterization of Lipo-Dcpep@MnPOM. (A) TEM image of Lipo-Dcpep@MnPOM. Scale bar, 50 nm. (B and C) Particle size and zeta potential analysis of different nanoformulations (Lipo, Lipo@MnPOM, Lipo-Dcpep@MnPOM) via DLS. (D) Particle size of various nanoformulations measured over 6 d. (E) Cumulative drug release curve of MnPOM in Lipo-Dcpep@MnPOM. (F) Cell viability measured by CCK-8 assay under different concentrations of MnPOM. (G) Targeting ability of different Dil-labeled liposomes (red) to DCs (green). Scale bar, 10 μm. (H) Flow cytometry analysis of intracellular uptake of Cy5.5-labeled liposomes. All values are presented as mean ± SD; *P < 0.05, **P < 0.01; *P* ≥ 0.05 is considered nonsignificant (NS).

To enhance MnPOM bioavailability in the DCs, we developed targeted liposomes (Lipo-Dcpep@MnPOM) using thin-film hydration. Dcpep, a positively charged 12-mer peptide, enables DC-specific targeting with high cellular uptake efficiency and favorable biosafety. Inductively coupled plasma mass spectrometry (ICP-MS) results demonstrated 79.14 ± 3.25% MnPOM encapsulation efficiency. TEM imaging results confirmed multilamellar morphology with a diameter of ~110 nm (Fig. 3A). Dynamic light scattering (DLS) analysis results (Fig. 3B) showed that the diameter of unmodified liposomes (Lipo) and MnPOM-loaded liposomes (Lipo@MnPOM) was ~100 nm, whereas 1,2-distearoyl-sn-glycero-3-phospho-ethanolamine (DSPE)–PEG2000 (polyethylene glycol, molecular weight 2000)–Dcpep modification increased the size of the liposomes to ~120 nm. This was consistent with the TEM results. Zeta potential analysis (Fig. 3C) demonstrated charge modulation for the free MnPOM (−9.81 mV), (2,3-dioleoyloxy-propyl)-trimethylammonium-chloride (DOTAP)-containing liposomes (+55.3 mV), and Dcpep-modified liposomes (+21.1 mV). This charge attenuation suggests electrostatic neutralization between cationic liposomes and anionic MnPOM. The positive charge and nanoscale size (~120 nm) enhances interaction of MnPOM with the DC membranes via electrostatic attraction, thereby promoting cellular uptake. Stability assessments showed minimal size variation over 1 week (Fig. 3D). Release kinetics demonstrated cumulative release of 90% MnPOM release within 2 weeks (Fig. 3E). Biocompatibility studies demonstrated that concentrations ≤140 μg/ml were safe and concentrations >140 μg/ml increased the cytotoxicity (Fig. 3F and Fig. S14). Flow cytometry (Fig. 3H) and confocal microscopy (Fig. 3G) results confirmed significantly enhanced DC uptake of the Dcpep-modified liposomes compared to the unmodified liposomes. This improved targeting was due to binding of Dcpep to DC-specific receptors (DEC205/CLEC9A/DC-SIGN), thereby facilitating receptor-mediated endocytosis [33].

**Fig. 4.**
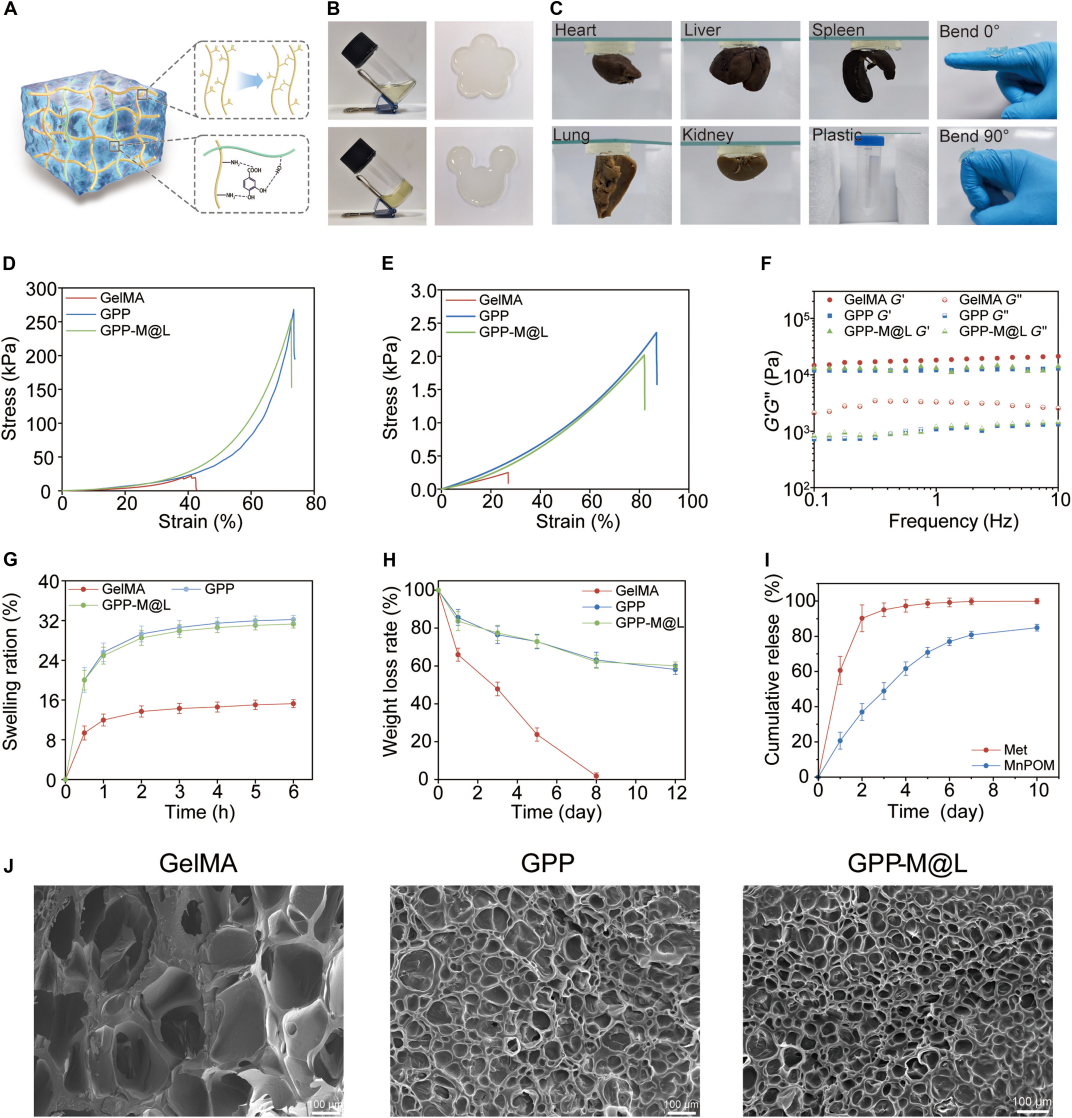
Preparation and characterization of GPP-M@L. (A) Gelation mechanism of GPP-M@L hydrogel. (B) Sol–gel transition image of GPP-M@L. (C) Adhesion and bending of GPP-M@L hydrogel. (D) Compressive stress–strain curves of GelMA, GPP, and GPP-M@L hydrogels. (E) Tensile stress–strain curves of GelMA, GPP, and GPP-M@L hydrogels. (F) Viscosity–frequency range (0.1 to 10 Hz) of different hydrogels. (G) Swelling ratio of different hydrogels. (H) Weight loss curve of hydrogels in PBS solution. (I) MnPOM and drug release curve from GPP-M@L hydrogel. (J) Representative SEM images of GelMA, GPP, and GPP-M@L hydrogels. Scale bar, 100 μm. All values are presented as mean ± SD; *P < 0.05, **P < 0.01; *P* ≥ 0.05 is considered nonsignificant (NS).

An ideal wound dressing biomaterial requires balanced mechanical strength, tissue adhesion, and biocompatibility. Notably, pure gelatin methacryloyl (GelMA) hydrogels lack sufficient mechanical integrity and exhibit brittleness under stress. To address this, we constructed a base GPP hydrogel (GelMA/PVA/PCA) via a one-pot method by integrating PVA and PCA into the GelMA network. This was further loaded with liposomes (Lipo-Dcpep@MnPOM) and Met, yielding the drug-loaded GPP-M@L hydrogel (Fig. 4A). The mechanical properties of the hydrogels were evaluated through tensile and compression tests. Compression tests indicated that the compressive strengths of GPP and GPP-M@L were 267.1 and 253.76 kPa, respectively (Fig. 4D), while their elastic moduli were approximately quadruple that of GelMA, matching the properties of human dermis [34]. Tensile tests revealed that the tensile performance of both GPP and GPP-M@L was superior to that of GelMA (0.25 kPa), with tensile strengths reaching 2.38 and 2.01 kPa, respectively (Fig. 4E), demonstrating enhanced toughness relative to GelMA. Rheological analysis (0.1 to 10 Hz) further confirmed that they had excellent elastic solid properties (stable *G*′ > *G*″) and structural stability (Fig. 4F). These results indicated that the incorporation of PVA and PCA significantly improved the mechanical strength of the base GPP hydrogel. The addition of PVA led to the formation of a PVA–GelMA interpenetrating network, which provided structural support, while the incorporation of PCA enhanced dynamic crosslinking within the hydrogel via hydrogen bonding. This physicochemical synergy markedly improved the mechanical performance of the base hydrogel. The mechanical properties of GPP-M@L remained highly consistent with those of the base GPP after drug loading, thus confirming that the encapsulation of liposomes and Met did not compromise the structural integrity of the hydrogel network.

Furthermore, GPP-M@L exhibited exceptional universal adhesiveness, firmly adhering to the surfaces of various tissues, including the heart, liver, spleen, lungs, and kidneys, and could even adapt to the 90° bending deformation of a finger joint (Fig. 4B and C). An evaluation of key functional attributes for wound dressings showed that GPP-M@L possessed excellent water absorption and swelling capacity (its equilibrium swelling ratio was 31 ± 0.5%, which was significantly higher than the 15 ± 0.9% recorded for GelMA), thereby maintaining an optimal hydrated state for healing (Fig. 4G) [35–37]. Degradation assays indicated that both GPP and GPP-M@L degraded more slowly than GelMA and remained incompletely degraded after 8 d, thus meeting the requirements for supporting tissue regeneration (Fig. 4H). Scanning electron microscopy (SEM) revealed that GPP and GPP-M@L possessed an interconnected porous network with an average pore size that was smaller than that of GelMA (Fig. 4J), which facilitated sustained drug release. Within 3 d, the cumulative release of Met reached 90%, while that of MnPOM reached 83.1% over 10 d (Fig. 4I).

Following confirmation that the GPP-M@L hydrogel possesses physicochemical properties that make it desirable as a wound dressing, we further evaluated the delivery efficiency and cellular targeting specificity of its loaded liposomes within the complex in vivo environment. Initially, through in vitro flow cytometric (Fig. S12) and confocal microscopic analysis (Fig. S13), we confirmed that Dcpep-modified liposomes (Lipo-Dcpep@MnPOM) were taken up by DCs with significantly higher efficiency compared with other phagocytic cell types (macrophages and endothelial cells), thereby confirming their superior DC-targeting capability. To verify whether these liposomes retained this specific targeting ability after release from the hydrogel in vivo, we conducted an in vivo distribution study. As shown in Fig. S11, immunohistochemical analysis [fluorescein isothiocyanate (FITC)-labeled liposomes, CD11c for DCs, CD68 for macrophages] in diabetic wounds 24 h post-modeling revealed strong colocalization of FITC and CD11c signals in the Lipo-Dcpep@MnPOM group. This indicated that the Lipo-Dcpep@MnPOM released from the GPP-M@L hydrogel was extensively internalized by DCs, with only minimal uptake by CD68^+^ macrophages. In contrast, liposomes without the targeting peptide (Lipo@MnPOM) were dispersed within the tissue interstitium and did not exhibit DC-specific uptake. These complementary in vitro and in vivo findings collectively demonstrated that the Dcpep-modified liposomes delivered by the GPP-M@L hydrogel are preferentially and efficiently internalized by DCs in diabetic wounds, thereby laying a crucial targeted delivery foundation for the subsequent initiation of the “efferocytosis cycle”.

### Biocompatibility and antibacterial activity of GPP-M@L

Cell Counting Kit-8 (CCK-8) assay results showed that MnPOM concentrations below 140 μg/ml did not show cytotoxicity but higher concentrations significantly reduced cell viability (Fig. 3F and Fig. S14). Excessive nanozymes may induce cytotoxicity by disrupting membrane integrity, whereas their intracellular accumulation causes dysregulated metabolism or ion homeostasis [23]. Live/dead staining results (Fig. S15) confirmed excellent biocompatibility of GPP-M@L hydrogel with minimal cell death comparable with the corresponding phosphate-buffered saline (PBS) controls at therapeutic concentrations. GPP-M@L demonstrated potent antibacterial activity against common pathogens (*Staphylococcus aureus* and *Escherichia coli*). Hydrogel extracts showed significant antimicrobial effects compared to the corresponding controls (Figs. S16 and S17). The inhibition rates were 80 ± 2.7% for *S. aureus* and 71 ± 1.4% for *E. coli* (Fig. S18). This broad-spectrum efficacy is potentially caused by PCA, which disrupts bacterial membrane permeability and interferes with essential metabolic processes to inhibit bacterial growth [38].

### Efficacy of GPP-M@L in modulating DC function

**Fig. 5.**
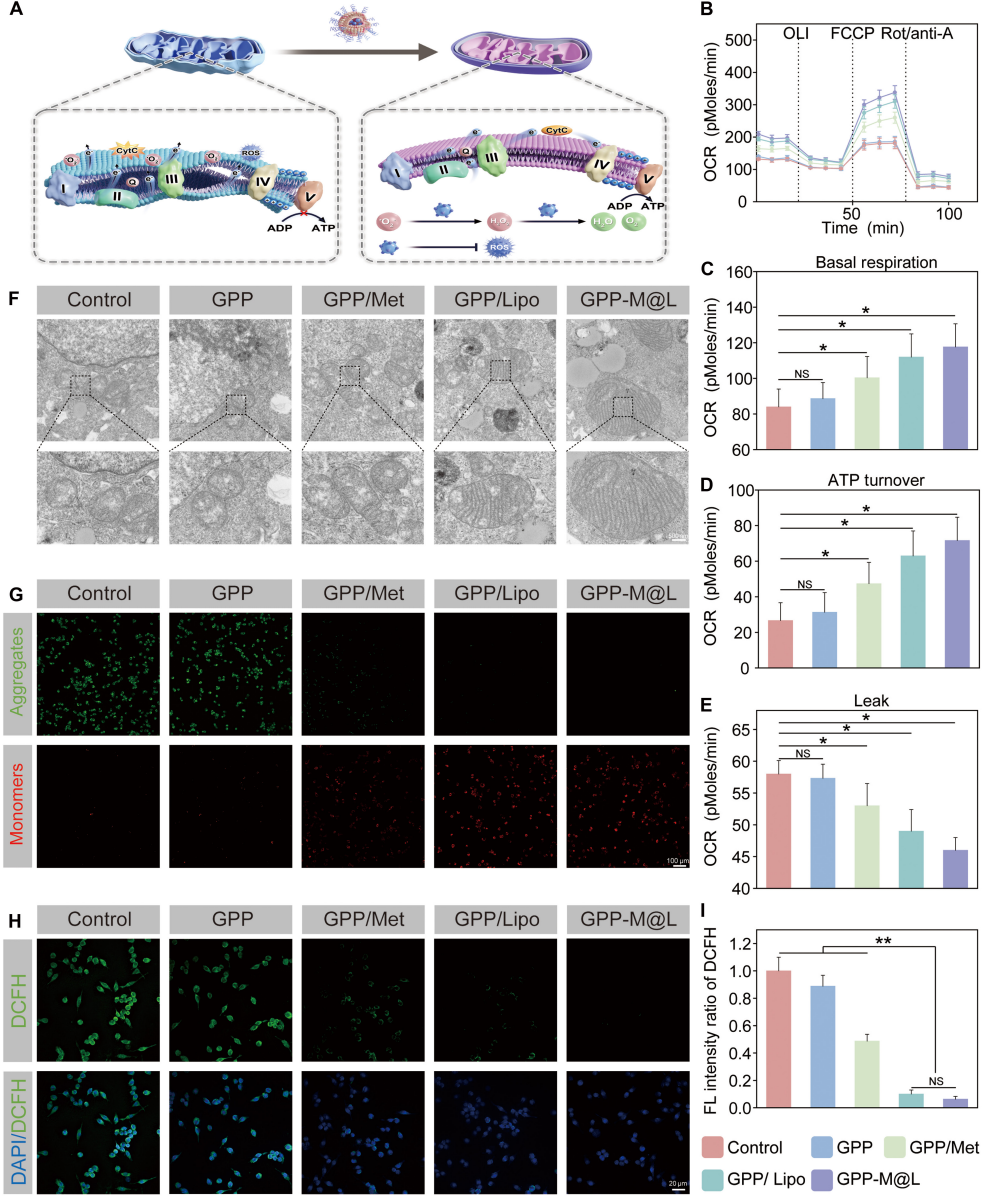
Regulation of DC MRC function by GPP-M@L. (A) Schematic diagram showing the effect of GPP-M@L hydrogel on MRC. (B) OCR of DC cells under different treatment groups. (C to E) Parameter analysis based on OCR results: basal respiration, ATP turnover, and proton leak. (F) Biological TEM images of intracellular mitochondria in different treatment groups. Scale bar, 500 nm. (G) Representative fluorescent images showing mitochondrial membrane depolarization interference in different groups, analyzed by JC-1 staining. Scale bar, 100 nm. (H) Images of DCFH-DA in different treatment groups; ROS (green), DAPI (blue). Scale bar, 20 μm. (I) Quantitative analysis of DCFH-DA expression. All values are presented as mean ± SD; *P < 0.05, **P < 0.01; *P* ≥ 0.05 is considered nonsignificant (NS). The control group represents hyperglycemia. GPP/Lipo denotes the abbreviated form of GPP/Lipo-Dcpep@MnPOM.

**Fig. 6.**
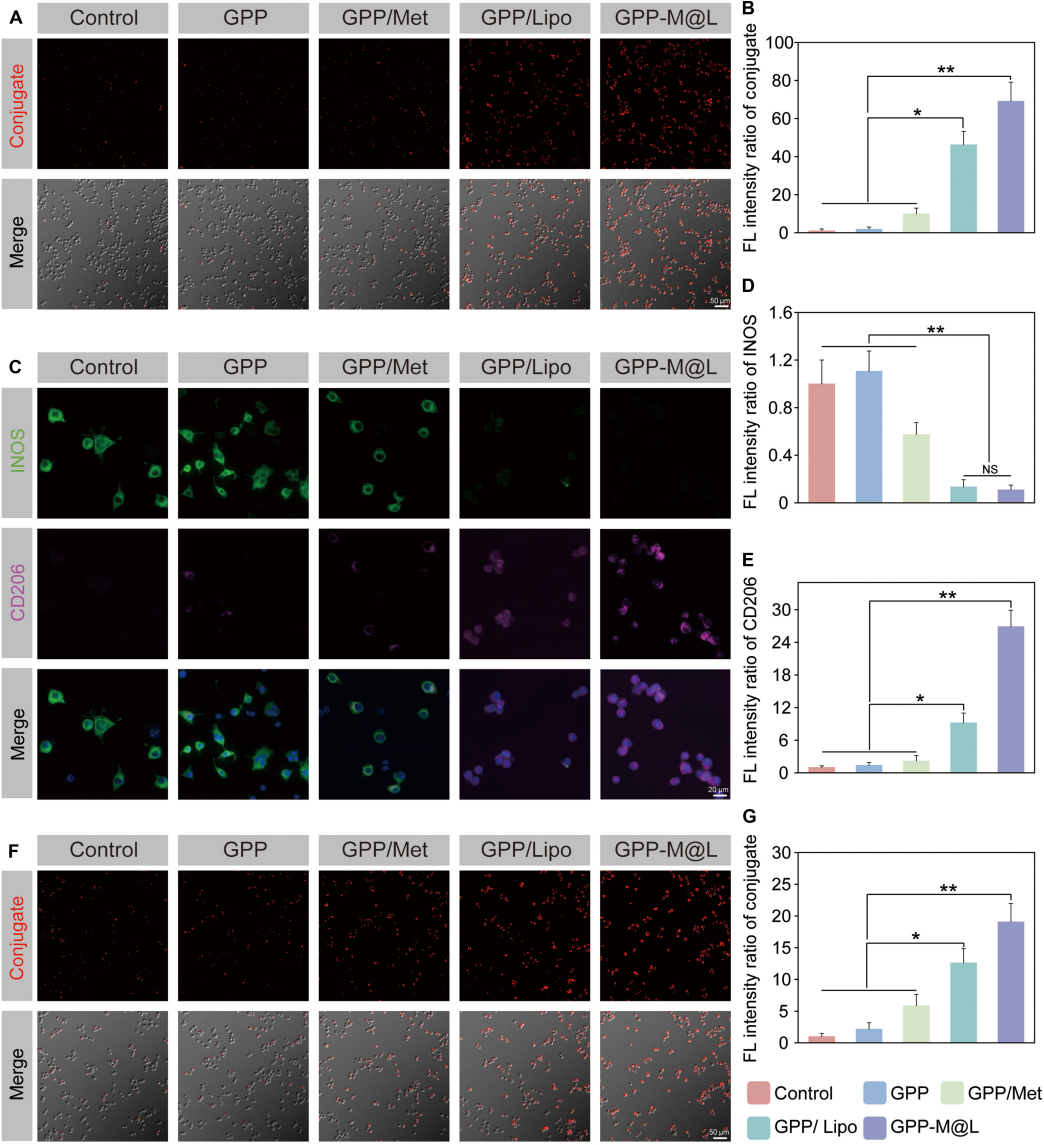
Regulation of efferocytosis in DC and macrophages by GPP-M@L. (A) Efferocytosis of DCs following treatment with different hydrogel groups as detected by conjugate product reaction. Scale bar, 50 μm. (B) Quantitative analysis of conjugate expression from (A). (C) Immunofluorescence images showing the expression of iNOS (green) and CD206 (red) in macrophages treated with different conditioned media. Nuclei were counterstained with DAPI (blue). Scale bar, 20 μm. (D) Quantitative analysis of iNOS expression. (E) Quantitative analysis of CD206 expression. (F) Efferocytosis of macrophages following treatment with different conditioned media as detected by conjugate product reaction. Scale bar, 50 μm. (G) Quantitative analysis of conjugate expression from (F). All values are presented as mean ± SD; *P < 0.05, **P < 0.01; *P* ≥ 0.05 is considered nonsignificant (NS). The control group represents hyperglycemia.

Persistence of diabetic wounds is caused by a vicious cycle involving hyperglycemia, immune dyshomeostasis, and impaired phagocyte efferocytic circulation. In this pathological microenvironment, DCs—central to efferocytic regulation—exhibit increased mitochondrial electron leakage. These electrons react with elemental oxygen to generate excessive levels of ROS. Elevated ROS directly damages oxidation-sensitive mitochondria (disrupting lipids, proteins, and DNA), disrupts mitochondrial function, and subsequently damages the adjacent organelles [39–41]. This cascade significantly reduces ATP production and severely impairs DC efferocytosis. Concomitant macrophage efferocytic dysfunction and impaired DC activity leads to accumulation of apoptotic cells and debris, thereby exacerbating immune dyshomeostasis. Therefore, therapeutic strategies should mitigate hyperglycemic oxidative stress, restore the functional MRC in DCs, and reactivate efferocytosis to reestablish immune homeostasis. Our data demonstrated that GPP-M@L hydrogel effectively improved DC functions (Fig. 5A). Oxygen consumption rate (OCR) measurements showed decreased basal respiration, increased proton leakage, and reduced ATP production in the DCs subjected to H₂O₂-induced oxidative stress compared to the corresponding controls (Fig. 5B to E). This confirmed impaired MRC in the H₂O₂-treated DCs. However, GPP-M@L treatment showed reduced proton leakage and restored ATP production in the DCs subjected to H₂O₂-induced oxidative stress. In this context, GPP-M@L treatment effect was better than the GPP/Met and GPP/Lipo controls. Mechanistically, MnPOM nanozymes mimic MnSOD by dismutating O₂^−^ into H₂O₂ and O₂, thereby preventing damage to the inner mitochondrial membrane; they also exhibit CAT activity to decompose H₂O₂ into H₂O and O₂, thereby synergistically alleviating oxidative stress. TEM results (Fig. 5F) demonstrated that GPP-M@L significantly improved mitochondrial structural integrity. TEM results showed severe cristae fragmentation in the control and GPP groups, moderate protection in the GPP/Met group, significant improvement in the GPP/Lipo group, and preserved cristae integrity in the GPP-M@L group. JC-1 fluorescence analysis (Fig. 5G) demonstrated that GPP-M@L significantly preserved mitochondrial membrane potential compared to the controls. Restoration of mitochondrial function also directly enabled recovery of DC function. Dichlorofluorescin diacetate (DCFH-DA) analysis (Fig. 5H and I) showed that GPP-M@L significantly reduced intracellular ROS in the hyperglycemic and oxidatively stressed DCs. Furthermore, pHrodo BioParticles efferocytosis assays (Fig. 6A and B) demonstrated that DC efferocytosis was severely impaired under pathological conditions (control group) but was significantly restored by treatment with GPP-M@L and GPP/Lipo. GPP-M@L enhanced energy-intensive efferocytosis by repairing mitochondrial structure and function and restoring ATP synthesis in the DCs. This mitigated the apoptotic cell accumulation–immune dysregulation cycle and accelerated wound healing.

### Efficacy of GPP-M@L in regulating macrophage efferocytosis

In the diabetic wounds, macrophage function is impaired and manifested by reduced efferocytic capacity and blocked M1-to-M2 phase transition. This exacerbates immune dyshomeostasis and impedes healing. Therefore, restoration of macrophage efferocytosis and M2 polarization is critical for effective therapy [42]. We hypothesized that modulating DC efferocytosis would enhance bidirectional DC–macrophage regulation in diabetic wounds. Mechanistically, efferocytosis-competent DCs enhance secretion of paracrine factors, including GDF15, a member of the transforming growth factor-β (TGF-β) superfamily [6,43]. Our data showed that GDF15 levels were higher in the GPP-M@L and GPP/Lipo groups compared to the controls (Fig. S21). Elevated GDF15 signaling functionally reshapes macrophage functions. In lipopolysaccharide (LPS)/high-glucose M1-polarizing conditions, GPP-M@L-conditioned medium significantly reduces the expression levels of proinflammatory inducible nitric oxide synthase (iNOS) (Fig. 6C and D) and enhances the levels of anti-inflammatory CD206 compared to all other groups (Fig. 6C and E). Consistently, immunofluorescence analysis demonstrated that GPP-M@L-treated DCs were more effective in promoting M2 macrophage repolarization than Met or manganese alone (Figs. S33 and S34). This confirmed that GPP-M@L-conditioned medium significantly promotes M2 repolarization under inflammatory conditions induced by LPS or high glucose. LPS/high-glucose-induced macrophages treated with GPP-M@L-conditioned medium exhibit restoration of optimal efferocytic function (Fig. 6F and G). Efferocytosis-restored macrophages reciprocally enhanced DC efferocytic function via paracrine signaling (Figs. S19 and S20), thereby establishing a positive feedback loop. Therefore, GPP-M@L restores DC efferocytosis by repairing mitochondrial function and stimulates DC-derived GDF15 synthesis and secretion to restore macrophage efferocytosis and M2 polarization. GPP-M@L also facilitates macrophage-mediated recovery of DC functions, thereby establishing a self-sustaining efferocytic circuit and reversing immune dysregulation in the diabetic wounds.

### GPP-M@L promotes vascularization–extracellular matrix coupled regeneration

**Fig. 7.**
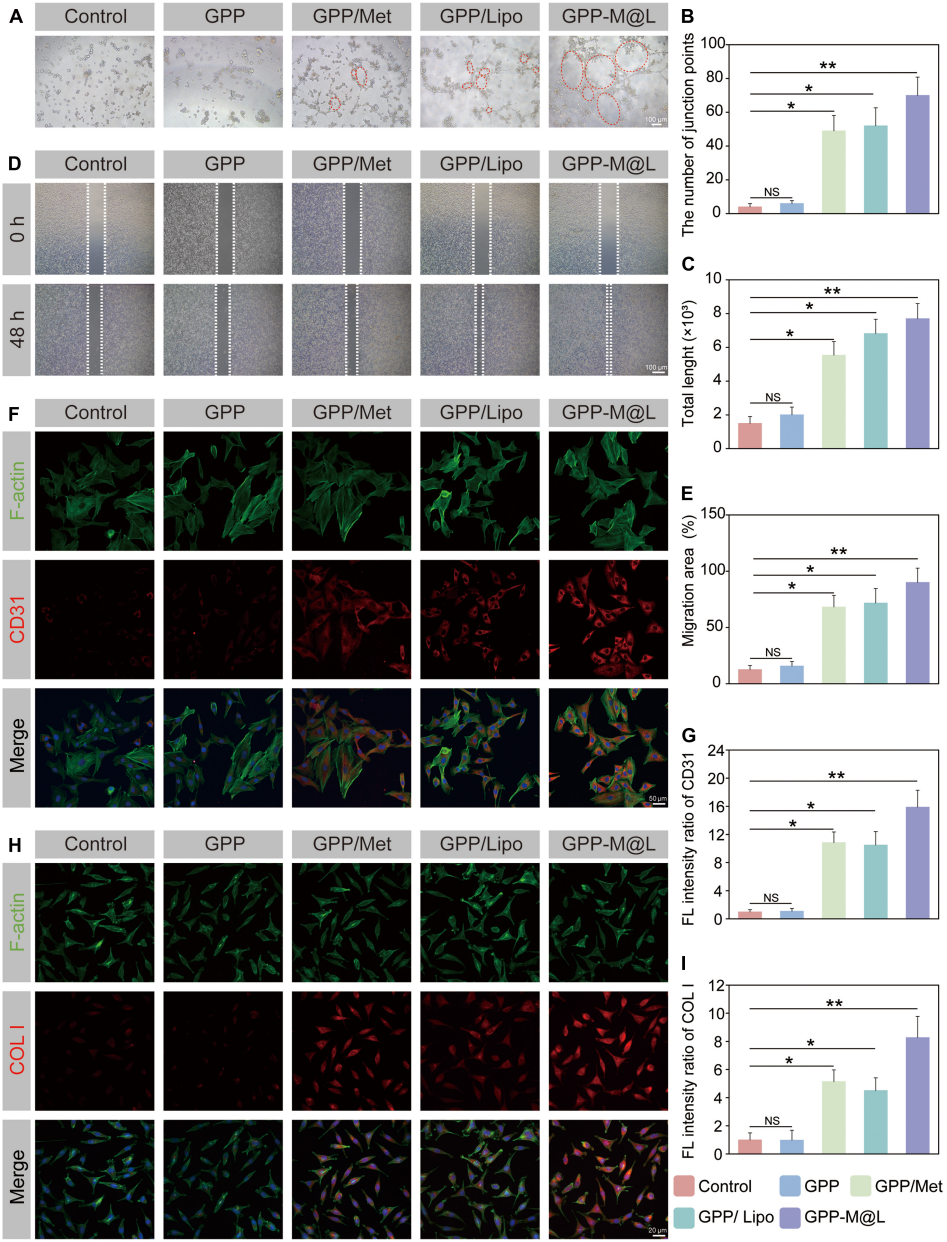
Efferocytosis modulators promote collagen production and angiogenesis in vitro. (A) Tube assay for different treatment groups. Scale bar, 100 μm. (B) Quantification of node number. (C) Quantification of total lumen diameter. (D) Cell migration assay in different treatment groups. Scale bar, 100 μm. (E) Quantitative analysis of cell migration rate. (F) Immunofluorescence images of CD31 expression in different treatment groups. F-actin (green), CD31 (red), DAPI (blue). Scale bar, 50 μm. (G) Quantitative analysis of CD31 expression. (H) Immunofluorescence images of COL I expression in different treatment groups. F-actin (green), COL I (red), DAPI (blue). Scale bar, 20 μm. (I) Quantitative analysis of COL I expression. All values are presented as mean ± SD; *P < 0.05, **P < 0.01; *P* ≥ 0.05 is considered nonsignificant (NS). The control group represents hyperglycemia.

Diabetic wound chronicity is caused by impairment of essential tissue regeneration processes, including cell migration, angiogenesis, and collagen deposition [44,45]. Restoration of phagocyte efferocytic circulation via GPP-M@L hydrogel promotes secretion of paracrine cytokines, which significantly enhance angiogenesis and collagen deposition, thereby remodeling the regenerative microenvironment. In vitro tube formation assay results demonstrated that under high glucose conditions, human umbilical vein endothelial cells (HUVECs) treated with conditioned medium from the control group exhibited impaired angiogenesis with sparse nodes and shortened tubules. However, HUVECs treated with GPP-M@L-conditioned medium generated the most robust vascular networks with significantly higher node counts and total tubule length compared to all other groups (Fig. 7A to C). Migration assays (Fig. 7D and E) demonstrated that GPP-M@L-conditioned medium enhanced HUVEC migration area by 6.9-fold compared to the controls. This demonstrated that GPP-M@L-conditioned medium significantly enhanced the migratory activity of HUVECs. Immunofluorescence experiments confirmed that GPP-M@L-conditioned medium significantly up-regulated endothelial CD31 expression (Fig. 7F and G) and fibroblast collagen I (COL I) production (Fig. 7H and I) compared to all the other groups. Collectively, these data showed that GPP-M@L-induced paracrine factors restored efferocytic circulation by stimulating endothelial migration, tubulogenesis, and fibroblast collagen synthesis, thereby accelerating diabetic wound healing.

### Molecular mechanisms underlying restoration of efferocytic circuit by GPP-M@L

**Fig. 8.**
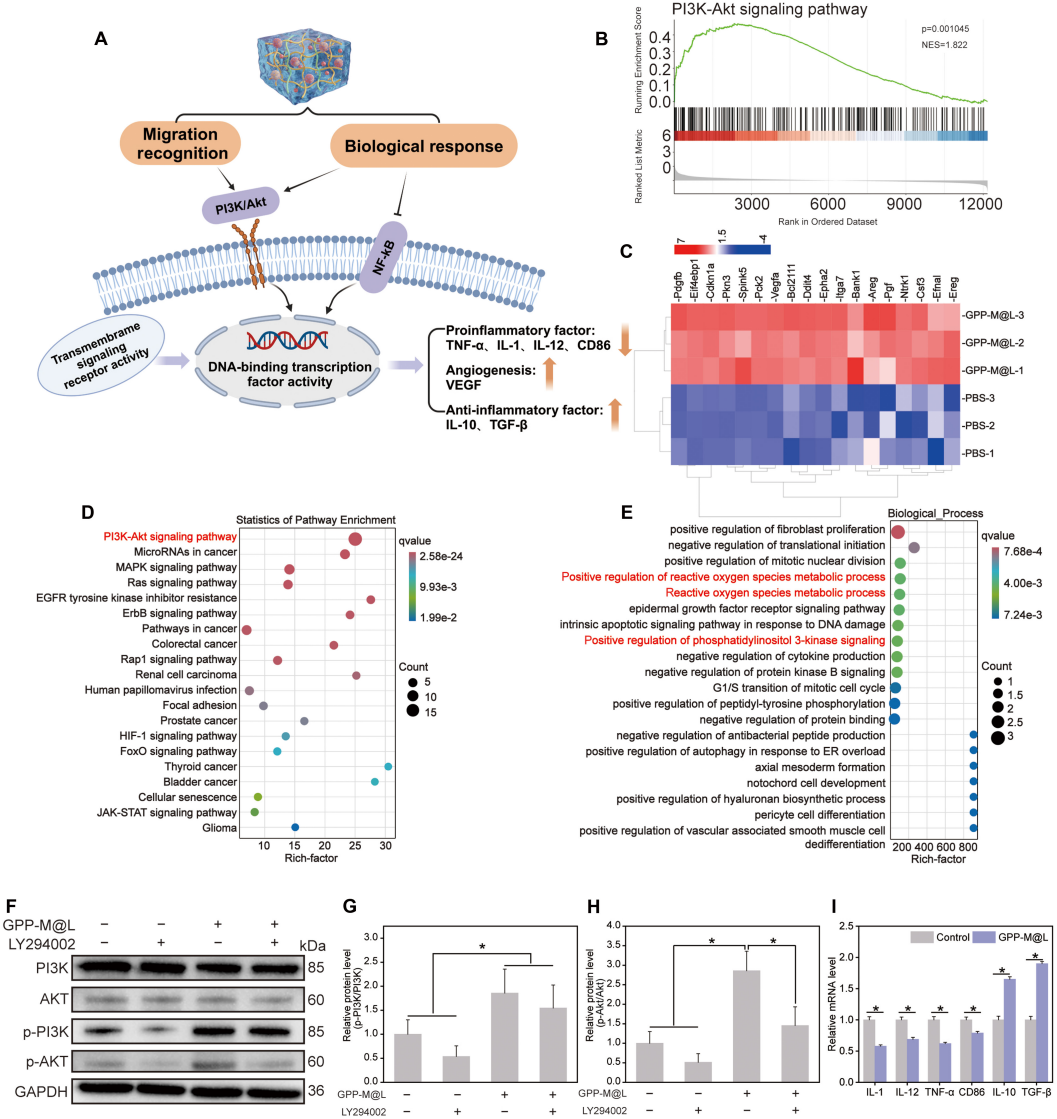
RNA-seq analysis of “circular efferocytosis”. (A) Diagram illustrating the promotion of macrophage efferocytosis and M2 polarization during the first phase. (B) Gene set enrichment analysis (GSEA) of the PI3K–Akt signaling pathway. (C) Normalized clustering analysis of differentially expressed genes regulated by PI3K/Akt. (D) KEGG pathway enrichment analysis of differentially expressed genes in the first phase. (E) Gene ontology (GO) biological process analysis of differentially expressed genes in the first phase. (F to H) Protein blotting assay and quantitative analysis. (I) Relative mRNA expression level of proinflammatory cytokines (IL-1, CD86, IL-12, and TNF-α) and anti-inflammatory cytokines (IL-10 and TGF-β) after treatment with GPP-M@L. All values are presented as mean ± SD; *P < 0.05, **P < 0.01; *P* ≥ 0.05 is considered nonsignificant (NS). The control group represents hyperglycemia.

We used transcriptomic analysis to elucidate the mechanism by which GPP-M@L restored phagocyte efferocytic circulation and decipher how efferocytosis-competent DCs regulate macrophage efferocytosis and M2 polarization through paracrine signaling (Fig. 8A). Transcriptomic analysis results showed 517 up-regulated and 137 down-regulated genes by comparing LPS-polarized M1 macrophages treated with GPP-M@L-conditioned medium versus PBS controls (Fig. S22). Gene set enrichment analysis (GSEA) (Fig. 8B) and Kyoto Encyclopedia of Genes and Genomes (KEGG) (Fig. 8D) demonstrated significant activation of the phosphatidylinositol 3-kinase (PI3K)–Akt pathway in the GPP-M@L groups. This suggested that the PI3K–Akt pathway regulates efferocytosis. Heatmap was used to visualize the differentially expressed genes (Fig. 8C). Gene ontology (GO) enrichment analysis results (Fig. 8E and Fig. S23) demonstrated that GPP-M@L enhanced regulation of PI3K signaling, ROS metabolism, epidermal growth factor (EGF) pathways, and growth factor activity, and down-regulated nuclear factor κB (NF-κB) signaling (Fig. S24). Western blotting demonstrated elevated p-PI3K and p-AKT levels in the GPP-M@L group without altering total PI3K and AKT protein levels, thereby confirming activation of the PI3K/Akt pathway (Fig. 8F to H). These effects were reversed by pretreatment with the PI3K inhibitor LY294002. However, GPP-M@L partially restored p-PI3K expression, thereby confirming PI3K/Akt-dependent restoration of macrophage efferocytosis and M2 polarization. Reverse transcription quantitative polymerase chain reaction (RT-qPCR) results confirmed phenotypic switching of macrophages, wherein GPP-M@L treatment down-regulated proinflammatory mediators such as IL-1β, IL-12, tumor necrosis factor-α (TNF-α), and CD86, and up-regulated anti-inflammatory factors such as IL-10 and TGF-β (Fig. 8I). Transcriptomic analysis also identified GDF15 as the pivotal cytokine mediating the DC–macrophage crosstalk. This cytokine coordinates dual regulation by suppressing NF-κB signaling to reduce proinflammatory factors while activating PI3K/Akt pathways. Activated PI3K/Akt enhances efferocytosis by driving actin polymerization/pseudopod formation via cytoskeletal regulators (MLC/Cofilin) and up-regulating phagocytic receptors (integrins/MerTK), and promotes the synthesis and secretion of anti-inflammatory cytokines, thereby inducing M2 polarization [46–51].

**Fig. 9.**
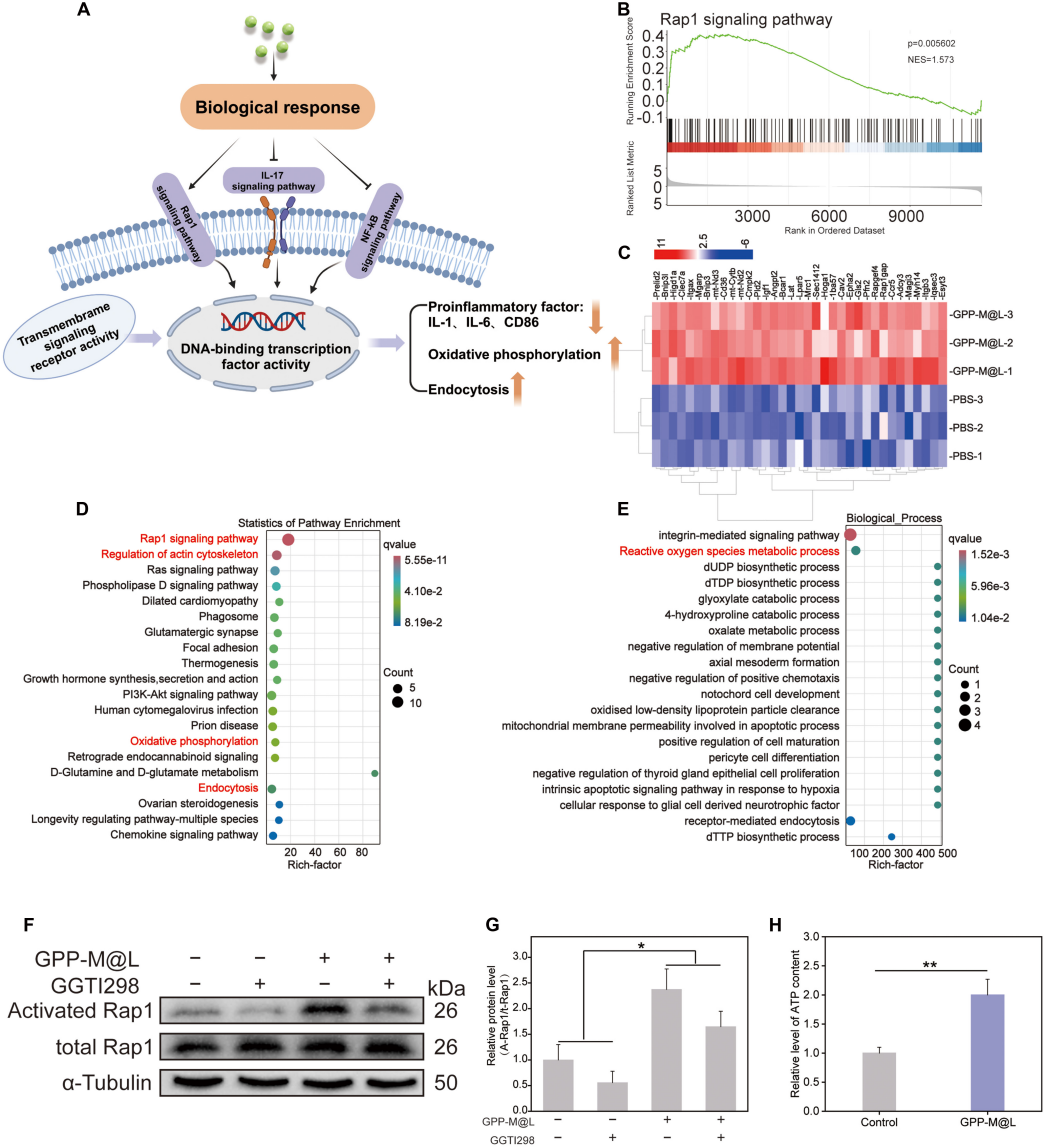
RNA-seq analysis of “circular efferocytosis”. (A) Diagram illustrating the promotion of DC efferocytosis during the second phase. (B) GSEA of the Rap1 signaling pathway. (C) Normalized clustering analysis of differentially expressed genes co-regulated by Rap1 and oxidative phosphorylation. (D) KEGG pathway enrichment analysis of differentially expressed genes in the second phase. (E) GO biological process analysis of differentially expressed genes in the second phase. (F and G) The activation levels of Rap1-GTP were measured using a Rap1 Activation Assay Kit. (H) Quantification of ATP production in the conditioned medium of different groups. All values are presented as mean ± SD; *P < 0.05, **P < 0.01; *P* ≥ 0.05 is considered nonsignificant (NS). The control group represents hyperglycemia.

We further elucidated macrophage-to-DC crosstalk mechanisms through secondary transcriptomics to determine the mechanism by which efferocytosis-restored macrophages enhanced DC function via paracrine signaling (Fig. 9A). Coculturing of GPP-M@L-treated macrophages with hyperglycemic DCs demonstrated 477 up-regulated and 248 down-regulated genes compared to the corresponding controls (Fig. S25). GSEA (Fig. 9B) and KEGG/GO analyses (Fig. 9C to E) demonstrated dual core mechanisms, including significant activation of oxidative phosphorylation with elevated NADH [reduced form of nicotinamide adenine dinucleotide (oxidized form)] dehydrogenase activity (Fig. S26) and enhanced efficacy of the mitochondrial electron transport chain. Furthermore, we observed increased ATP production (Fig. 9H) in the GPP-M@L group. ATP is necessary for DC efferocytosis. Coactivation of Rap1 signaling promotes cytoskeletal reorganization and endocytosis. Western blotting and Rap1-GTP (guanosine triphosphate) assays (Fig. 9F and G) confirmed specific Rap1 activation in the hyperglycemic DCs cocultured with GPP-M@L-treated macrophages. Pretreatment with the Rap1 inhibitor GGTI298 reversed Rap1 activation, but GPP-M@L partially preserved activity. This confirmed the role of Rap1 in enhancing DC proliferation, cytoskeletal remodeling, and apoptotic cell recognition. Concurrent down-regulation of the IL-17/NF-κB pathway (Fig. S27) generated a favorable microenvironment by reducing proinflammatory factors. These data demonstrated that macrophage-derived signals reciprocally enhanced DC efferocytosis through dual pathways to establish a self-sustaining molecular circuit that included oxidative phosphorylation, ATP production and Rap1 signaling, and modulation of cellular function. Elevated levels of vascular endothelial growth factor (VEGF) and platelet-derived growth factor (PDGF) (Fig. S28) confirmed that restoration of efferocytic circulation involved paracrine factors that positively regulated endothelial and fibroblast functions.

### GPP-M@L restores efferocytic circulation and promotes functional diabetic wound regeneration

**Fig. 10.**
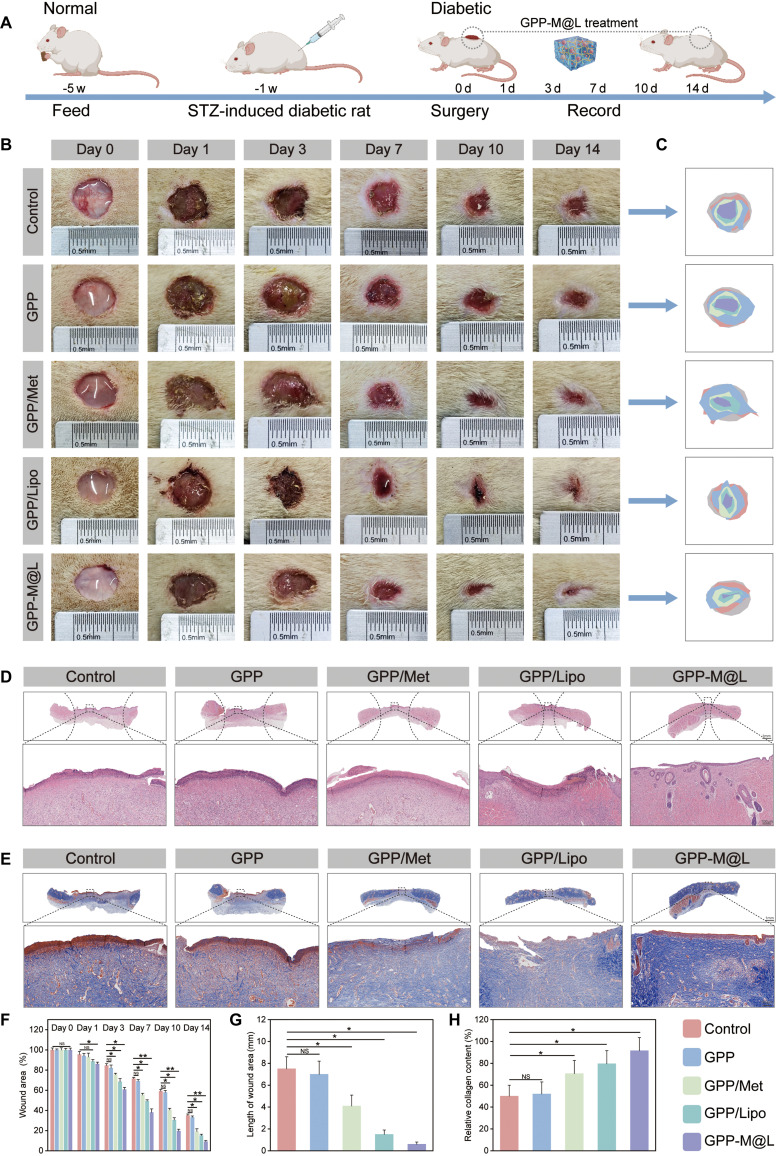
In vivo study of the efficacy of the efferocytosis modulator in promoting diabetic wound healing. (A) Schematic representation of the application of GPP-M@L hydrogel in animal experiments. (B) Representative images of the diabetic wound healing process at days 0, 1, 3, 7, 10, and 14. Scale bar, 1 mm. (C) Representative images of diabetic wound scars at days 0, 1, 3, 7, 10, and 14. (D) H&E staining of skin tissue at day 14. Scale bars, 1 mm and 100 μm. (E) Masson staining of skin tissue at day 14. Scale bars, 1 mm and 100 μm. (F) Quantitative analysis of the residual area percentage. (G) Quantitative analysis of the wound edge distance. (H) Quantification of collagen deposition percentage. All values are presented as mean ± SD; *P < 0.05, **P < 0.01; *P* ≥ 0.05 is considered nonsignificant (NS). The control group represents hyperglycemia.

**Fig. 11.**
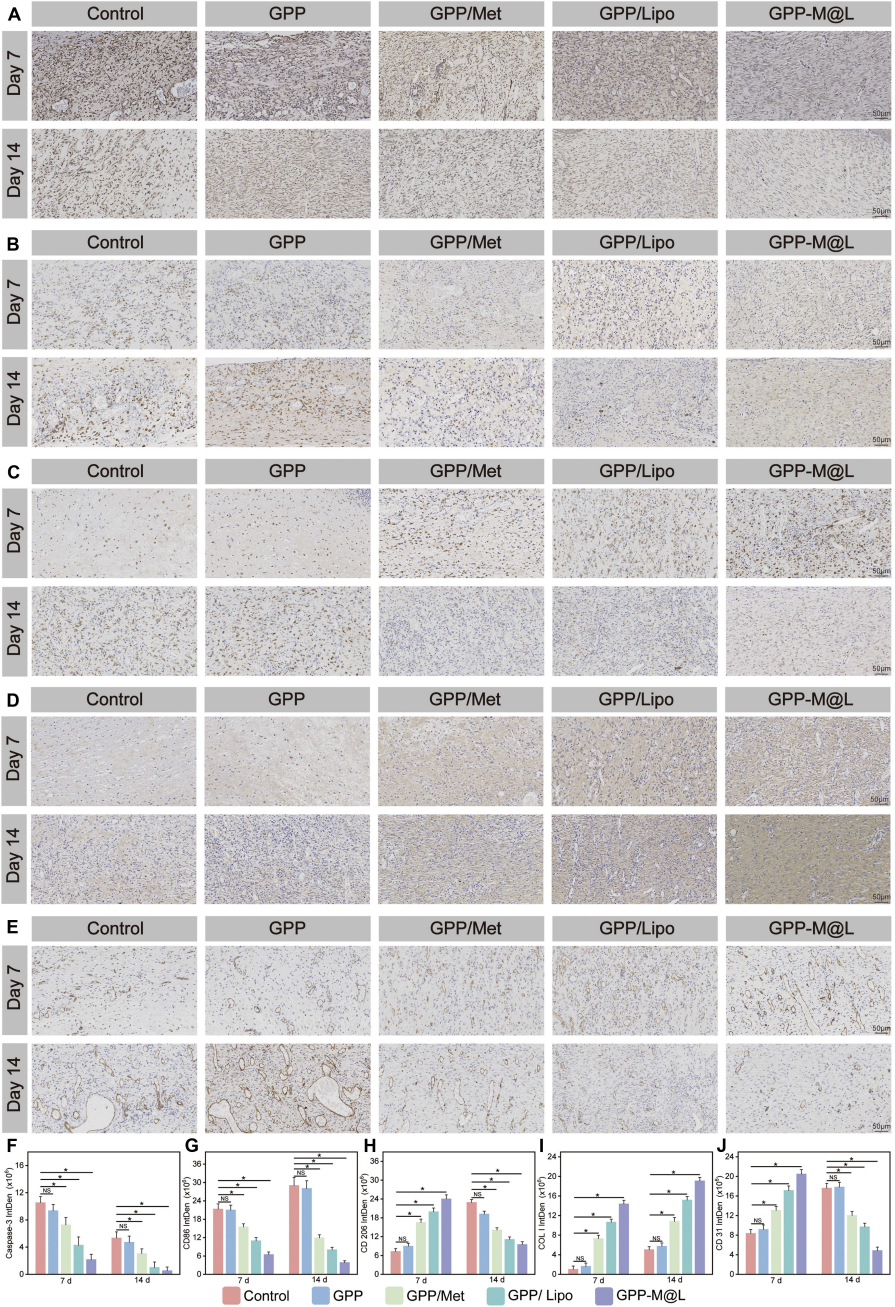
Histological analysis of animal experiments. (A to E) Immunohistochemical staining for caspase-3, CD86, CD206, COL I, and CD31 at days 7 and 14 (scale bar, 50 μm). (F to J) Quantitative analysis of respective marker expression. Data are presented as mean ± SD; *P < 0.05, **P < 0.01; NS, not significant. The control group was under hyperglycemic conditions.

To systematically evaluate the in vivo efficacy of the GPP-M@L hydrogel, we established diabetic mouse models for multidimensional assessment. The treatment strategy is shown in Fig. 10A. Wound monitoring demonstrated progressive wound contraction without infection in all the groups over 14 d, but the control group exhibited persistent erythema and exudates. Quantitative analysis demonstrated significantly accelerated healing in the GPP-M@L and GPP/Lipo groups compared to the control, with significant therapeutic synergy with the Met–MnPOM combination. Meanwhile, local glucose measurements (Fig. S29) revealed that glucose levels at the wound sites of mice treated with GPP/Met or GPP-M@L hydrogels were significantly reduced compared with those of control animals. Specifically, the glucose levels decreased from baseline values of 14.4 and 14.1 mM to 7.3 and 7.4 mM, respectively, values that approached the normal physiological glucose range for mice (reference value: approximately 4.65 ± 0.14 mM). This effective regulation of glucose levels markedly alleviated the detrimental effects of the high-glucose microenvironment on cellular function, thereby creating favorable conditions for the restoration of efferocytosis and further promoting the wound healing process. Additionally, when we extended the observation period, we found that the glucose-lowering effect mediated by the GPP-M@L hydrogel lasted approximately 80 h (Fig. S30), which closely aligned with its drug release profile. This indicated that GPP-M@L establishes a regenerative microenvironment conducive to wound healing before the complete release of Met. On day 14, wound closure area was 60% in the control group, 84% in the GPP/Lipo group, and 92% in the GPP-M@L group (Fig. 10B, C, and F). Hematoxylin and eosin (H&E) staining results (Fig. 10D and G) showed that the control group wounds contained scabs and inflammatory infiltrates at the dermal–epidermal junctions on day 14, indicating inflammatory phase arrest. Conversely, the GPP-M@L group exhibited minimal wound gaps with nascent hair follicles and sebaceous glands, thereby confirming enhanced tissue reconstruction and improved inflammatory microenvironment. Masson’s trichrome staining results (Fig. 10E and H) demonstrated increased collagen deposition density in the GPP-M@L group compared with all the other groups. This highlighted the potent extracellular matrix (ECM) remodeling capacity of the GPP-M@L hydrogels. To further investigate the enhancement of mitochondrial ATP generation in vivo, a proposed key mechanism for restoring efferocytosis, we performed immunofluorescence staining for ATP5A, a core subunit of ATP synthase, whose levels reflect mitochondrial ATP production capacity. The results showed that ATP5A fluorescence intensity was significantly higher in the GPP-M@L group than in all the other groups on day 7, the peak period of efferocytic activity (Figs. S31 and S32). This indicated that the potential for ATP production was substantially elevated, which is essential for powering the energy-demanding process of efferocytosis. The subsequent enhancement of apoptotic cell clearance, as evidenced by the minimal accumulation of caspase-3-positive cells in the GPP-M@L group from day 7 to day 14 (Fig. 11A and F), strongly supports that the observed increase in ATP generation directly contributed to more efficient efferocytosis. By day 14, the ATP5A signal in the GPP-M@L group decreased to levels comparable to those in the other groups, consistent with a reduction in energy requirements during the tissue remodeling phase.

Immunohistochemistry results showed the status of efferocytic circulation. Caspase-3 staining (Fig. 11A and F) demonstrated minimal accumulation of apoptotic cells in the GPP-M@L group between days 7 and 14, thereby demonstrating efficient clearance. CD86 (M1 marker) expression decreased progressively in the treatment groups (Fig. 11B and G), whereas CD206 (M2 marker) peaked on day 7 to resolve inflammation and physiologically declined by day 14 during tissue remodeling (Fig. 11C and H). These results suggested normal progression of wound healing. Furthermore, we also observed advanced regeneration with coordinated angiogenesis and collagen maturation: Immunohistochemical staining of COL I (Fig. 11D and I) showed highest early expression on day 7 and denser, organized fiber arrangement on day 14 in the GPP-M@L group. CD31 staining (Fig. 11E and J) demonstrated robust early angiogenesis (strongest signal on day 7) and physiological decline during late remodeling, consistent with mature wound histology. Furthermore, peak CD206 expression coincided with maximal angiogenesis, whereas its decline aligned with collagen maturation. This validated programmed immunomodulation of tissue regeneration.

In summary, our engineered efferocytosis regulator—GPP/Met/Lipo-Dcpep@MnPOM (GPP-M@L)—disrupts the vicious cycle of hyperglycemia–immune dyshomeostasis in the refractory diabetic wounds. It establishes dynamic phagocyte efferocytic circulation, remodels the regenerative microenvironment, and ultimately accelerates diabetic wound regeneration.

## Conclusion

In this study, we developed an efferocytosis–immune homeostasis regulatory system that restores for treating chronic diabetic wounds by restoring the phagocyte efferocytic circuits. DC-targeted liposomes deliver MnPOM nanozymes that repair mitochondrial respiratory complexes via SOD/CAT activities, restore ATP-dependent DC efferocytosis, and enhance paracrine-mediated macrophage M2 polarization and efferocytic function. Reciprocally, M2 macrophages release reparative factors that further enhance DC efferocytosis and establish a self-sustaining DC–macrophage efferocytic circuit. This circuit remodels the wound regenerative microenvironment by coordinately improving endothelial and fibroblast functions, thereby accelerating tissue regeneration. Our integrated strategy centered on restoring the efferocytic circuit and establishes a new therapeutic paradigm for diabetic wounds, with broader applicability to immunodysregulation-related pathologies. The clinical translation of biomaterials faces multiple systemic challenges, including scalability, process standardization, long-term biosafety evaluation, and regulatory compliance. These challenges constitute significant barriers to the translation of laboratory research into clinical applications. Within this broader context, the GPP-M@L system developed in this study must also address corresponding translational requirements. These include further validation of efficacy in large animal models, the establishment of a Good Manufacturing Practice (GMP)-compliant manufacturing process for its multi-component system, clarification of the regulatory pathway as a combination drug-device product, and assessment of the impact of individual variability on treatment outcomes in clinical practice. While these translational hurdles are common across the field, the therapeutic potential of the GPP-M@L system, shown by its ability to restore the “efferocytosis cycle” immunomodulatory mechanism, provides a valuable foundation and a developmental direction for its future clinical translation.

## Materials and Methods

### Chemical reagents and kits

Manganese chloride (catalog no. 7773-01-5), lecithin (catalog no. 8002-43-5), cholesterol (catalog no. 57-88-5), DOTAP (catalog no. 252769-92-9), and PVA (CAS number 9002-89-5) were obtained from Shanghai Macklin Biochemical Technology Co. Ltd. (Shanghai, China). 3,4-Dihydroxybenzoic acid (CAS number 99-50-3) was obtained from Shanghai Yuanye Bio-Technology Co. Ltd. (Shanghai, China). Ammonium molybdate tetrahydrate (catalog no. 12054-85-2) and vitamin C (catalog no. 50-81-7) were obtained from Shanghai Aladdin Biochemical Technology Co. Ltd. (Shanghai, China). DAPI (catalog no. AR1176) was purchased from Boster Biological Technology Co. Ltd. (Wuhan, China). Paraformaldehyde (PFA) (catalog no. P1110), dialysis membranes (catalog nos. YA1078 and YA1071), and lithium phenyl-2,4,6-trimethylbenzoylphosphinate (LAP) (catalog no. L2090) were purchased from Beijing Solarbio Science & Technology Co. Ltd. (Beijing, China). Cell Counting Kit-8 (catalog no. C0037), Live-Dead Staining Kit (catalog no. L0465), and JC-1 Mitochondrial Membrane Potential Assay Kit (catalog no. C2003S) were obtained from Biyuntian Biotechnology Co. Ltd. (Shanghai, China). COL I antibody (catalog no. AF7001), CD31 antibody (catalog no. AF6191), CD86 antibody (catalog no. DF6332), CD206 antibody (catalog no. DF4149), iNOS antibody (catalog no. AF0199), PI3K antibody (catalog no. AF6241), AKT antibody (catalog no. AF6261), phospho-AKT antibody (catalog no. AF0016), and caspase-3 antibody (catalog no. AF7022) were obtained from Affinity Biosciences Group Ltd. (Jiangsu, China). ROS probe (catalog no. D1008) was acquired from Uelandy Co. Ltd. (Suzhou, China). Streptozocin (STZ) (catalog no. 2196) and FITC–phalloidin (catalog no. 2284MG05) were purchased from Guangzhou Saiguo Biotech Co. Ltd. (Guangzhou, China). pHrodo BioParticles Conjugates (catalog no. P35361, Invitrogen), DMEM (catalog no. 11965118), and fetal bovine serum (FBS) (catalog no. A5256701) were purchased from GibcoBRL (Rockville, MD, USA). Matrigel (catalog no. 354234) was acquired from Corning, USA. The hydroxyl radical test kit, CAT assay kit (Visible light), SOD assay kit (WST-1 method), and anti-superoxide ion test kit were obtained from Jiancheng Biological Engineering Research Institute (Nanjing, China). Steady pure quick RNA extraction kit (catalog no. AG21024) was obtained from Accurate Biotechnology Co. Ltd. (Hunan, China). The peptide with a purity of >97% was purchased from Apeptide Co. Ltd. (Shanghai, China). GelMA was purchased from EFL-Tech (Suzhou) Co. Ltd. (Suzhou, China). The Mouse Growth Differentiation Factor 15 (GDF15) ELISA Kit is designed to measure GDF15 levels in mouse samples. The item (catalog no. E-EL-M0604) was acquired from Elabscience Biotechnology Co. Ltd. (Hunan, China). Rap1 Pulldown Activation Assay Kit (catalog no. 81401) was purchased from Wuhan NewEast Biosciences Co. Ltd. (Hunan, China).

### Synthesis and characterization of MnPOM

MnPOM was synthesized via a one-pot reduction technique. Ammonium molybdate tetrahydrate (0.36 mmol) was dissolved in 10 ml of ultrapure water with constant stirring at 25 °C, and then 1.08 mmol of MnCl₂ solution was added. Ascorbic acid solution (4 ml) was then added, and the mixture was stirred for 2 more hours to ensure that the reaction was complete. The solution was dialyzed for 7 h, freeze-dried, and stored at 4 °C. The morphology of MnPOM was examined using TEM (Talos F200i, USA), and its elemental composition was determined through elemental mapping. XPS (ESCALAB Qxi, USA) was used to analyze elemental composition and valence states. Fourier transform infrared (FTIR) spectroscopy (Thermo Scientific Nicolet iS5, USA) and ESR spectroscopy (Bruker A300, Germany) were employed to analyze functional groups and oxygen vacancies.

### Synthesis and characterization of the targeting peptide

Dcpep was linked to DSPE–PEG2000–NHS through *N*-hydroxy succinimide (NHS) ester–amine coupling. Dcpep was dissolved in dimethylformamide (DMF) and combined with DSPE–PEG2000–NHS at a 2:1 molar ratio. The mixture was gently stirred for 12 h at room temperature in the dark. The solution underwent dialysis against pure water for 24 h using a 2,000-Da molecular weight cutoff membrane. The dialyzed solution was freeze-dried and purified by size exclusion chromatography to obtain the final product. High-performance liquid chromatography (HPLC) was used to assess conjugation efficiency. The successful synthesis of DSPE–PEG2000–Dcpep was confirmed by ^1^H nuclear magnetic resonance (NMR) (Figs. S8 to S10).

### Synthesis and characterization of liposomes

Lipo-Dcpep@MnPOM was synthesized via the thin-film hydration technique. Phosphatidylcholine, cholesterol, DOTAP, and DSPE–PEG2000–Dcpep were dissolved in a 9:1 chloroform–methanol solvent mixture at an 8:2:1:2 mass ratio in a pear-shaped flask. A thin lipid film was formed by removing the organic solvents through rotary evaporation under vacuum. The film was then hydrated with 10 ml of PBS containing 1.4 mg of MnPOM and mixed constantly by vortexing. The dispersion was then probe-sonicated at 20% amplitude for 10 min and sequentially filtered through 0.22-μm membranes to obtain Lipo-Dcpep@MnPOM. DLS using Zetasizer Nano S (Malvern Instruments, UK) was employed to estimate the particle size and zeta potential of Lipo-Dcpep@MnPOM. The morphology was examined using TEM (JEM-1400plus, Japan) following staining with 5% phosphotungstic acid and air drying. ICP-MS (Agilent Technologies, USA) was used to quantify the encapsulation efficiency and drug release properties of Lipo-Dcpep@MnPOM.

### Preparation of GPP-M@L hydrogel

The GPP-M@L hydrogel was prepared using a straightforward one-pot method. Briefly, an aqueous solution of PVA was first prepared. Then, GelMA, PCA, Lipo-Dcpep@MnPOM, and Met were added to this solution and stirred until they dissolved fully. The GPP-M@L hydrogel, consisting of GelMA, PCA, PVA, Met, and Lipo-Dcpep@MnPOM, was formed by injecting the solution into molds and exposing it to UV light for 3 min.

### Characterization of in vitro enzyme activities of MnPOM

The H₂O₂ scavenging capacity of MnPOM solutions (0 to 400 μg/ml in PBS) was assessed by comparing UV–visible (Vis) spectra before and after a 10-min reaction with 1 mM H₂O₂. The residual levels of H₂O₂ after the scavenging reactions were quantified by iodometry. This involved recording a standard curve using H₂O₂ standards (1 to 10 mM) after reaction with potassium iodide. Absorbance at 580 nm was measured, and scavenging efficacy was determined using the following formula: H₂O₂ scavenging (%) = [(*A*₀ − *A*₁)/*A*₀] × 100%, where *A*₀ represents the absorbance of the H₂O₂ control and *A*₁ denotes the absorbance of the MnPOM-treated sample. A dissolved oxygen meter was used to estimate oxygen production every 30 s, and the kinetics of oxygen generation was determined by plotting oxygen concentration versus time.

The ABTS assay was employed to evaluate the radical scavenging capacity of MnPOM using 2,2′-azino-bis (3-ethylbenzothiazoline-6-sulfonic acid). ABTS radical cation solution was prepared by reacting ABTS with potassium persulfate for 16 h at room temperature in darkness. The solution’s absorbance at 734 nm was set to 0.70 ± 0.02 using a UV–Vis spectrophotometer. Solutions with different concentrations of MnPOM (0 to 20 μg/ml) were prepared. Each solution was mixed with an equal volume of ABTS radical cation solution and incubated for 10 min in the dark. Subsequently, absorbance was measured spectrophotometrically.

Hydroxyl radical scavenging ability was evaluated using a commercial detection kit. The working hydroxyl radical test solutions were prepared according to the manufacturer’s specifications. The MnPOM solutions (0 to 400 μg/ml) were combined with the samples using vortexing and incubated for 1 min at 37 °C. Then, the chromogenic agent was added immediately and the mixture was incubated for 20 min at room temperature. Absorbance was recorded at a wavelength of 550 nm. The hydroxyl radical inhibition capacity (U/ml) is determined using the formula: [(*A*₀ − *A*₁)/(*A*₂ − *A*₃)] × *C* × (1/*V*) × *N*, where *A*₀ represents the control group absorbance, *A*₁ is the test sample absorbance, *A*₂ denotes the standard group absorbance, *A*₃ is the blank group absorbance, *C* is the standard concentration (8.824 mM), *V* is the sample volume (0.2 ml), and *N* is the dilution factor.

Superoxide anion scavenging capacity was measured using a commercial detection kit. The xanthine/xanthine oxidase system was used to generate O₂^−^ radicals. MnPOM solutions (0 to 30 μg/ml) were introduced to the reaction system and maintained at 25 °C for 30 min. Absorbance was recorded at a wavelength of 550 nm. The superoxide anion scavenging capacity (U/ml) is determined using the formula [(*A*₀ − *A*₁)/(*A*₀ − *A*₂)] × *C* × *N*, where *A*₀, *A*₁, and *A*₂ represent the absorbance of the control, test sample, and standard group, respectively. *C* denotes the standard concentration (0.15 mg/ml), and *N* is the dilution factor. CAT and SOD activities were evaluated using enzyme assay kits following the manufacturer’s guidelines.

Each experiment was conducted 3 times.

### Liposome targeting assay

Bone marrow-derived dendritic cells (BMDCs) were maintained in RPMI 1640 medium with 10% FBS at 37 °C and 5% CO_2_. Cells were incubated with Cy5.5-labeled liposomes (Lipo@MnPOM or Lipo-Dcpep@MnPOM) for 4 h, after which viable cells were collected and resuspended in PBS. The liposome uptake efficiency of cells was analyzed by flow cytometry (Sony SA3800, Japan). For the colocalization analysis to verify liposome targeting, BMDCs treated with Cy5.5-labeled liposomes were counterstained with DAPI to visualize the nuclei and phalloidin to stain the F-actin cytoskeleton. After PBS washing, cells were visualized using confocal microscopy (Olympus, Japan) to assess liposome–BMDC interactions.

### GPP-M@L targeting assay

Different types of phagocytic cells were incubated with the extract of GPP-M@L, which contained Lipo-Dcpep@MnPOM labeled with Cy5.5, for 4 h. Subsequently, live cells were collected and resuspended in PBS, after which liposome uptake efficiency was analyzed using flow cytometry (Sony SA3800, Japan). For colocalization analysis to confirm liposome targeting, different types of phagocytic cells were treated with Dil-labeled liposomes and then counterstained with DAPI to visualize nuclei and phalloidin to stain the F-actin cytoskeleton. After washing with PBS, the cells were visualized under a confocal microscope (Olympus, Japan) to evaluate liposome–BMDC interactions.

To assess the targeted distribution of Lipo-Dcpep@MnPOM in diabetic wounds, we performed in vivo immunofluorescence experiments. A diabetic full-thickness skin defect model was established in Sprague–Dawley rats, which were subsequently randomly divided into an experimental group (Lipo-Dcpep@MnPOM) and a control group (Lipo@MnPOM). Wound tissues were harvested 24 h post-modeling, fixed in 4% PFA, embedded in paraffin, and sectioned. After deparaffinization, rehydration, and antigen retrieval in citrate buffer, the sections were blocked and then incubated with primary antibodies against CD11c and CD68 to label DCs and macrophages, respectively. After incubation with secondary antibody for 1 h, nuclei were counterstained with DAPI, and the sections were mounted. Images were acquired using a laser scanning confocal microscope.

### Physical properties of the hydrogel

Rheological properties were assessed using a Haake Mars 40 rheometer (Germany) by conducting frequency sweeps at a constant strain to measure the storage modulus (*G*′) and loss modulus (*G*″) over the frequency range. Flow scanning tests were conducted from 0.1 to 10 Hz at room temperature.

Mechanical properties: The Instron 5982 electromechanical testing system (Instron, USA) was used to perform several tests to determine the mechanical properties of the hydrogel. Compression testing was performed to evaluate cylindrical hydrogels (12 mm diameter × 6 mm height). Compression modulus was calculated within the linear region (20% to 40% strain). The samples were compressed at 2 mm/min until fracture. Tensile testing was performed on rectangular hydrogel specimens (50 mm × 1 mm × 2 mm). The samples were stretched at 15 mm/min until failure.

Swelling properties: Swelling behavior was evaluated by gravimetry. Hydrogels were immersed in PBS at 25 °C. Samples were periodically removed, blotted with filter paper to eliminate surface moisture, and then weighed. The swelling ratio (%) is determined using the following formula: [(*Wt* − *W*0)/*W*0] × 100, where *W*0 represents the initial mass and *Wt* denotes the mass at time *t*.

### Hydrogel morphology

The hydrogel’s microstructure was analyzed using SEM with Zeiss Gemini 360 from Germany. Samples were freeze-dried, sputter-coated with gold, and imaged by SEM.

### In vitro biodegradation of the hydrogel

Hydrogel samples (500 μl) were incubated in PBS at pH 7.4 and 37 °C. Subsequently, samples were retrieved at different time points, rinsed thrice with ultrapure water, freeze-dried, and weighed. The weight loss percentage was determined using the following formula: Weight loss (%) = [(*W*0 − *Wt*)/*W*0] × 100, where *W*0 represents the initial dry weight and *Wt* denotes the dry weight at time *t*.

### In vitro drug release

The release kinetics of MnPOM from liposomes or GPP hydrogel were assessed by sealing samples with 140 μM MnPOM in 1,000-Da dialysis bags, which were then immersed in 5 ml of PBS (pH 7.4) and incubated at 37 °C with agitation at 80 rpm. Then, at specific time points, we removed 1 ml of release medium for analysis and replaced with fresh PBS. The total amount of MnPOM released over 10 d was quantified by ICP-MS. Met release kinetics from the GPP hydrogel (2.0 mg/ml) were evaluated using identical methods. The total amount of Met released from the GPP hydrogel was estimated by UV–Vis spectrophotometry. The experiments were conducted 3 times.

### Assessment of in vitro biocompatibility

The biocompatibility of MnPOM was evaluated using a CCK-8 cytotoxicity assay. HUVECs, RAW 264.7, and L929 cells (5 × 10^3^ cells per well) were cultured in 96-well plates with MnPOM (0 to 180 μg/ml) in Dulbecco’s modified Eagle’s medium (DMEM) for 48 h. After removing the medium, CCK-8 working solution (100 μl per well) was added and incubated for 30 min at 37 °C. The absorbance was then analyzed at 450 nm. Cell viability was adjusted relative to untreated controls. The tests were conducted in triplicate.

Live/Dead staining: Cell viability and morphology were evaluated by live/dead staining kit. Hydrogel extracts were prepared according to Chinese National Standard GB/T 16886.5-2017. Control, GPP, GPP/Met, GPP/Lipo, and GPP-M@L (0.2 g/ml) were incubated in DMEM for 24 h at 37 °C to prepare extracts. HUVECs (5 × 10^4^ cells per well) were cultured in 24-well plates, treated with extracts for 24 h, and subsequently stained using a live/dead solution for imaging via inverted fluorescence microscopy. All the assays were repeated thrice.

### Antibacterial activity assay

Antibacterial properties of the hydrogel were evaluated using the spread plate method. Following the gelation of hydrogels (Control, GPP, GPP/Met, GPP/Lipo, and GPP-M@L; 200 μl) in 48-well plates, a 10-μl bacterial suspension [10^7^ colony-forming units (CFU)/ml] was applied to the hydrogel surfaces. The plates were exposed to ambient light and incubated at 37 °C for 2 h. Subsequently, bacteria from each well were collected and resuspended in 1 ml of sterile PBS. Bacterial suspension (10^7^ CFU/ml in PBS) without hydrogel treatment was designated as the negative control. We then distributed 100 μl of bacterial suspensions onto agar plates. The plates were maintained at 37 °C for 20 h. The bacterial colonies were photographed and quantified with ImageJ software. Each experiment was conducted 3 times.

Bacterial morphology: Bacterial morphology after hydrogel treatments was analyzed by SEM. Bacterial suspensions, whether control or hydrogel-treated, were centrifuged at 4,000*g* for 10 min at 25 °C. Bacterial pellets were collected and fixed overnight at 4 °C using 2.5% glutaraldehyde. Following glutaraldehyde removal, samples underwent PBS washing and ethanol series dehydration (30% to 100%, 15 min per step). Then, 20-μl aliquots were air-dried on silicon wafers, sputter-coated with gold, and imaged by SEM.

### Seahorse metabolic analysis

BMDCs (10^5^ cells per well) were plated in XF-24 microplates (Agilent Technologies) and left to incubate overnight. Cells were exposed to 100 μM H₂O₂ to induce oxidative stress and subsequently incubated for 12 h with hydrogel extracts (Control, GPP, GPP/Met, GPP/Lipo, or GPP-M@L). Cellular OCR was subsequently measured using an XF-24 Extracellular Flux Analyzer (Agilent Technologies). Mitochondrial function was evaluated using sequential injections: 2.0 μM oligomycin for ATP-linked respiration, 1 μM carbonyl cyanide 4-(trifluoromethoxy)phenylhydrazone (FCCP) for maximal respiratory capacity, and 0.5 μM antimycin A combined with 0.5 μM rotenone to inhibit mitochondrial respiratory complex activity. Each experiment was conducted 3 times.

### Analysis of intracellular ROS, mitochondrial membrane potential, and mitochondrial morphology

To induce oxidative stress, 1 × 10^6^ BMDCs were seeded in confocal dishes and exposed to 100 μM H₂O₂. Cells were exposed to hydrogel extracts (Control, GPP, GPP/Met, GPP/Lipo, or GPP-M@L) for 24 h. Post-exposure, the extracts were aspirated, and cells were incubated with DCFH-DA solution at 37 °C in the dark for 30 min. Nuclei were counterstained with DAPI for 10 min before confocal microscopy imaging. ROS expression was quantified by analyzing the fluorescence intensity of DCFH-DA using the ImageJ software. Mitochondrial membrane potential was assessed using the JC-1 kit (Biosharp, China). BMDCs in different experimental groups were stained with JC-1 for 30 min. Then, they were analyzed by confocal imaging. To evaluate the effects of hydrogel treatment on mitochondrial morphology, the cells were scraped and centrifuged at 1,500*g* for 5 min. After removing the supernatant, the cell pellets were fixed in ice-cold 2.5% glutaraldehyde. Samples were sectioned into 70- to 90-nm slices using a Leica EM UC7 ultramicrotome following dehydration. Sections were stained with uranyl acetate and lead citrate for 5 to 10 min, air-dried, and analyzed using a Hitachi H-7650 TEM. Each experiment was conducted 3 times.

### Efferocytosis assay

To induce oxidative stress, 1 × 10^6^ BMDCs were seeded in confocal dishes and exposed to 100 μM H_2_O_2_. Cells were exposed to hydrogel extracts (Control, GPP, GPP/Met, GPP/Lipo, or GPP-M@L) for 24 h. Following aspiration of the extracts, the cells were incubated with pHrodo BioParticles Conjugates (Invitrogen P35361) at a 1:5 cell-to-particle ratio for 4 h at 37 °C and 5% CO_2_. Efferocytosis was visualized by confocal fluorescence microscopy. Fluorescence intensity was quantified using the ImageJ software to determine efferocytic capacity. Each experiment was conducted 3 times.

### GPP-M@L hydrogel experiments to enhance macrophage efferocytosis and modulate phenotype

To simulate the pathological microenvironment of oxidative stress and apoptotic accumulation, BMDCs (1 × 10^6^ cells) were exposed to 100 μM H_2_O_2_ and UVC-induced apoptotic cells (incubated for 4 h under 150 mJ/cm^2^ UVC at 37 °C with 5% CO_2_). The cells were then treated with hydrogel extracts (Control, GPP, GPP/Met, GPP/Lipo, or GPP-M@L) for 24 h. The conditioned supernatants (“Supernatant 1”) were collected, mixed with complete medium at a specified ratio, and used as conditioned medium for subsequent macrophage polarization assays.

Efferocytosis assay: To simulate immune dysregulation in diabetic wounds, RAW 264.7 macrophages (1 × 10^6^ cells) were induced into the M1 phenotype using 200 ng/ml LPS and 100 μM H_2_O_2_. The cells were exposed to supernatant 1 for 24 h. The cells were then incubated for 4 h at 37 °C with 5% CO_2_ using pHrodo BioParticles (Invitrogen P35361) at a 1:5 cell-to-particle ratio. Efferocytosis was assessed via confocal microscopy, and fluorescence intensity was quantified with ImageJ software. The experiments were conducted 3 times.

Phenotype analysis: RAW 264.7 cells were polarized into the M1 phenotype as described above for the efferocytosis assay. The cells were incubated with the conditioned medium for 24 h, followed by fixation with 4% PFA for 30 min at room temperature. They were then permeabilized using 0.1% Triton X-100 for 15 min and blocked with a rapid blocking solution for 20 min. Cells were incubated overnight at 4 °C with primary antibodies targeting anti-CD206 and iNOS, followed by a 2-h incubation with fluorescent secondary antibodies at room temperature in the dark. DAPI was used to counterstain nuclei for 10 min. Macrophage polarization was analyzed by confocal microscopy and ImageJ software. The assays were repeated thrice.

Immunofluorescence quantification of polarization: To further evaluate macrophage phenotypic switching, M1-polarized RAW 264.7 cells were treated with the conditioned medium as above. After 24 h of incubation, the cells were fixed, permeabilized, and blocked. They were then costained with antibodies against iNOS (M1 marker) and CD206 (M2 marker), followed by incubation with appropriate fluorescent secondary antibodies and DAPI nuclear staining. Fluorescence images were captured using a confocal microscope. The fluorescence intensities of iNOS and CD206 were quantified with ImageJ software, and the M2/M1 polarization ratio was calculated based on the ratio of CD206 to iNOS signal intensity. Three independent experiments were performed.

### Evaluating DC efferocytic capacity after treatment with efferocytosis-restored macrophage

Supernatant 2 preparation: Polarized M1 macrophages and apoptotic cells were cocultured with group-specific supernatant 1 for 24 h. Then, the conditioned medium (supernatant 2) was collected by centrifugation. BMDCs cultured in high-glucose conditions were exposed to supernatant 2 for 24 h. Post-aspiration, cells were incubated with pHrodo BioParticles (Invitrogen P35361) at a 1:5 cell-to-particle ratio at 37 °C with 5% CO_2_ for 4 h. Efferocytosis was assessed using confocal microscopy and ImageJ software. Each experiment was conducted 3 times.

### Evaluating effects of GPP-M@L hydrogel on endothelial and fibroblast function

Cell migration assay: HUVECs (1 × 10^5^ cells per well) were cultured to confluence in 6-well plates at 37 °C with 5% CO₂. Then, scratch wounds were created in the monolayer using 200-μl pipette tips. Cells were exposed to serum-free supernatant mixtures (1/2) from the Control, GPP, GPP/Met, GPP/Lipo, or GPP-M@L groups. Wound areas were captured at 0 and 36 h using CapStudio and analyzed with ImageJ software. The migration rate was determined using the formula: Migration (%) = (*A*₁/*A*₀) × 100, where *A*₀ represents the initial wound area and *A*₁ denotes the area at the final time point.

For the tube formation assay, Matrigel was thawed on ice, combined in equal parts with serum-free DMEM, and allowed to polymerize in 96-well plates at 37 °C. HUVECs (3 × 10^4^ cells per well) were seeded on the Matrigel and treated with serum-free supernatant 1/2 mixtures from the Control, GPP, GPP/Met, GPP/Lipo, or GPP-M@L groups for 18 h. The tube networks were imaged by bright-field microscopy, and the angiogenic capacity was quantified using the ImageJ software.

HUVECs (1 × 10^4^ cells) were cultured in confocal dishes and exposed to serum-free supernatant mixtures (1/2) from the Control, GPP, GPP/Met, GPP/Lipo, or GPP-M@L groups for 48 h. Subsequently, cells were fixed with 4% PFA at room temperature for 30 min. Cells underwent permeabilization with 0.1% Triton X-100 for 15 min, followed by a 20-min blocking step. The samples were incubated overnight at 4 °C with the anti-CD31 primary antibody and then exposed to a fluorescent secondary antibody for 2 h at room temperature in the dark. The actin cytoskeleton was labeled using FITC–phalloidin, while the nuclei were stained with DAPI for 10 min. CD31 and COL I levels were quantified by confocal microscopy and ImageJ. Fibroblasts were also analyzed using an identical protocol. Each experiment was conducted 3 times.

### GDF15 quantification and transcriptomic analysis

GDF15 levels in supernatants were quantified using the mouse/rat ELISA kit (Elabscience; catalog no. E-EL-M0604). Transcriptomic profiles of macrophages cocultured with supernatant 1 samples from various experimental groups were analyzed by mRNA sequencing (mRNA-seq). To simulate immune dysregulation, macrophages were cultured in 6-well plates and treated with 200 ng/ml LPS for 24 h. Cells were treated with PBS or supernatant 1 samples from various experimental groups for 24 h. Then, mRNA samples were prepared using TRIzol (Biomarker Technologies, Beijing) in triplicate biological replicates. An identical mRNA-seq analysis was performed for the BMDCs cultured with supernatant 2 samples from various experimental groups under high-glucose conditions.

Western blot analysis: Whole-cell protein lysates were prepared using radioimmunoprecipitation assay (RIPA) buffer (Beyotime) supplemented with protease inhibitors. Protein concentrations were measured using the bicinchoninic acid (BCA) assay from Beyotime. Protein samples (20 μg each) were denatured in Laemmli buffer at 100 °C for 10 min, subjected to sodium dodecyl sulfate–polyacrylamide gel electrophoresis (SDS-PAGE), and transferred onto 0.45-μm polyvinylidene difluoride (PVDF) membranes (Millipore). Membranes were incubated with 5% bovine serum albumin (BSA) for 1 h to block, followed by overnight probing with primary antibodies at 4 °C. The blots were washed 3 times with tris-buffered saline Tween (TBST) for 5 min each and then incubated with horseradish peroxidase (HRP)-conjugated secondary antibodies for 1 h at room temperature. The blots were developed and visualized using chemiluminescence, and protein bands were quantified with the ImageJ software.

Rap1-GTP levels were assessed utilizing the Rap1 activation assay kit from NewEast Biosciences (catalog no. 81401). Samples were incubated with active Rap1 antibody and protein A/G agarose at 4 °C for 1 h. The beads were then pelleted at 5,000*g* for 1 min, washed, resuspended in loading buffer, and subjected to Western blot analysis.

ATP quantification: ATP levels were measured using the CheKine kit (Abbkine; catalog no. KTB1016) according to the manufacturer’s instructions.

RT-qPCR: Gene expression levels of IL-1β, CD86, TNF-α, IL-4, IL-12, IL-10, VEGF, and PDGF were analyzed by RT-qPCR. All experiments were conducted with 3 independent biological replicates.

### In vivo measurement of glucose levels in the wound periphery in diabetic model mice

Blood was collected from the periphery of wounds of diabetic mice from the different gel formulation treatment groups (Control, GPP, GPP/Met, GPP/Lipo, and GPP-M@L) at various time points (0, 3, 6, 12, 24, 48, 72, 96, and 120 h). Blood glucose levels were monitored using a glucometer (GA-3, Sinocare Inc., China).

### Animal study

Rats weighing 200 to 250 g were subjected to a high-fat/high-sucrose diet for 4 weeks to induce glucose intolerance. Type 2 diabetes was induced by intraperitoneal streptozotocin (STZ; 35 mg/kg). Fasting blood glucose ≥16.7 mM at 72 h post-STZ confirmed successful modeling of diabetes. Diabetic rats were randomly assigned to 5 groups and anesthetized using sodium pentobarbital at a dose of 20 mg/kg. Following dorsal sterilization, biopsy punches were used to create full-thickness wounds with a 10-mm diameter. Then, they were treated with GPP, GPP/Met, GPP/Lipo, or GPP-M@L hydrogels and the control. Wounds were photographed and analyzed with the ImageJ software on days 0, 1, 3, 7, 10, and 14 post-treatment. The percentage of wound closure was determined using the following formula: Wound area (%) = (*A*₁/*A*₀) × 100, where *A*₀ represents the initial wound area and *A*₁ denotes the area at a given time point. At the endpoint, rats were euthanized (pentobarbital, 150 mg/kg, intraperitoneally). Wound tissues were harvested and fixed in 4% formaldehyde. Tissues were evaluated by H&E staining, Masson’s trichrome staining, and immunohistochemistry for ATP5A, caspase-3, CD31, CD86, CD206, and COL I. ImageJ software was used to quantify epithelial gap, collagen density, CD31^+^ vessels (angiogenesis), and COL I deposition (regeneration). Biocompatibility was evaluated on day 14 using H&E staining of sections from major organs, including the heart, liver, spleen, lungs, and kidneys. Experiments were conducted in triplicate biological replicates.

### Statistical analysis

Statistical data were analyzed using the SPSS 24.0 (IBM) and Origin 2022 (OriginLab) software. Independent *t* tests were used to evaluate differences between 2 groups, while one-way analysis of variance (ANOVA) was employed for comparisons among multiple groups. Results are presented as mean ± standard deviation (SD). Statistical significance was indicated as follows: NS for not significant, * for *P* < 0.05, ** for *P* < 0.01, and *** for *P* < 0.001.

## Ethical Approval

The Ethics Committee of North Sichuan Medical College approved all animal experiments (approval number NSMC2024095).

## Data Availability

The study’s data can be obtained from the corresponding author upon reasonable request.
