# Supplementary material for: Manganese-Based Polyoxometalate Nanozyme-Metformin Co-functionalized Hydrogel Promotes Diabetic Wound Regeneration by Enhancing Phagocyte Efferocytosis
Source: Research (Wash D C). 2025 Nov 27;8:0964. doi: 10.34133/research.0964 (PMC12697061; doi:10.34133/research.0964)
Supplement: Supplementary 1 — Figs. S1 to S34 [file research.0964.f1.zip › Supplementary Information_Clean Version.docx]

*Supplementary information:*

**Manganese-Based Polyoxometalate Nanozyme-Metformin Co-Functionalized Hydrogel Promotes Diabetic Wound Regeneration by Enhancing Phagocyte Efferocytosis**

*Linfeng Li^1, 3#^, Jing Zhang^1, 3, 4#^, Caiping Yan^1, 3#^, Yongjie Wen^1, 3^, Lifu Jia^1^, Xiyang Tang^2^, Yuan Yong^2, 3, 5^, Ke Jiang^1^, Hanfeng Yang^2, 3^*, Lu Chen^1^*, Yuling Li^1, 2, 3^**

*^1^* Department of Orthopedics, Laboratory of Biological Tissue Engineering and Digital Medicine, Affiliated Hospital of North Sichuan Medical College, Nanchong, Sichuan 637000, PR China

*^2^* Biotechnology Innovation Drug Application and Transformation Key Laboratory of Sichuan Province, North Sichuan Medical College, Nanchong, Sichuan 637000, China

*^3^* Institute of Nanomedicine Innovation and Translational Research, Affiliated Hospital of North Sichuan Medical College, Nanchong, Sichuan 637000, PR China

*^4^* Department of Gastroenterology, Affiliated Hospital of North Sichuan Medical College, Nanchong, Sichuan 637000, PR China

*^5^* School of Chemistry and Environment, Southwest Minzu University, Chengdu, Sichuan 610041, PR China

E-mail: yhf5@nsmc.edu.cn (H. Yang), nsmccl@aliyun.com (L. Chen), lyl1987@nsmc.edu.cn (Y. Li)

^#^ These authors contributed equally to this work.


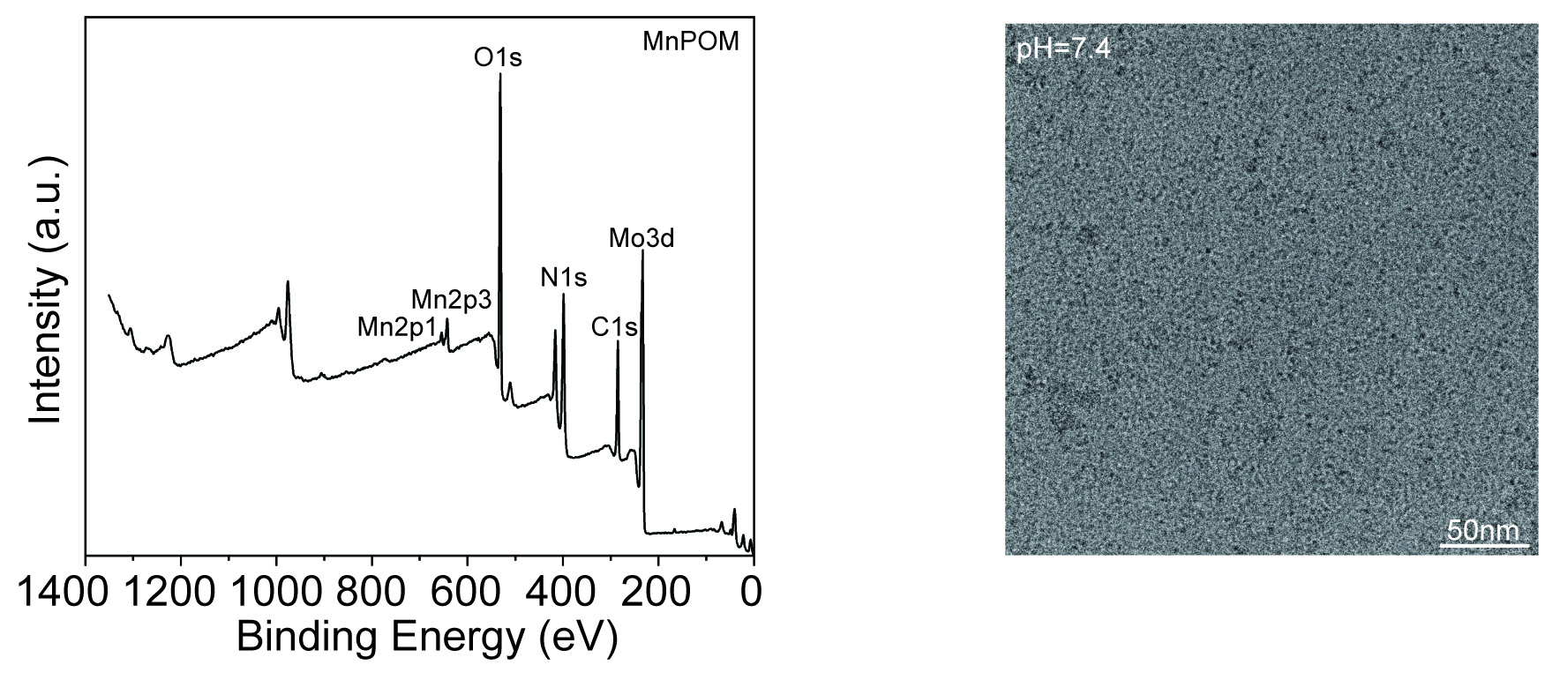


Figure S1: High-resolution XPS spectrum of MnPOM and TEM at pH = 7.4.


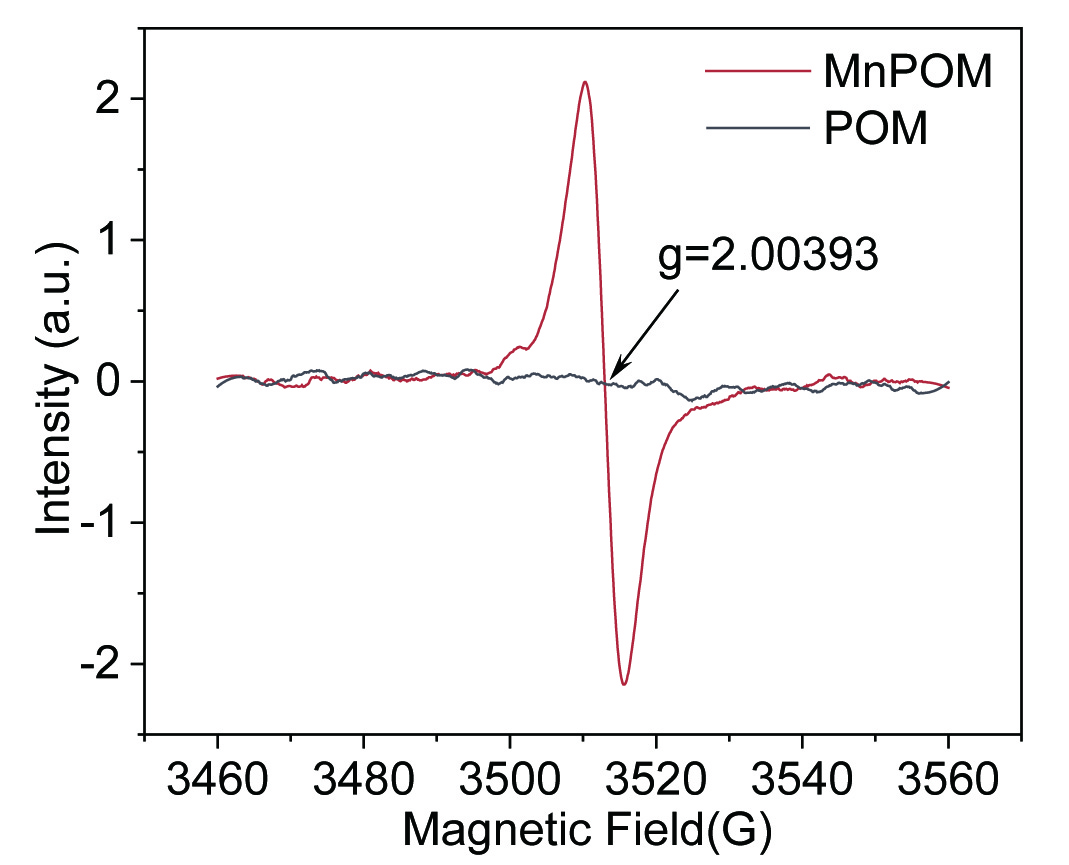


Figure S2: ESR of POM and MnPOM.


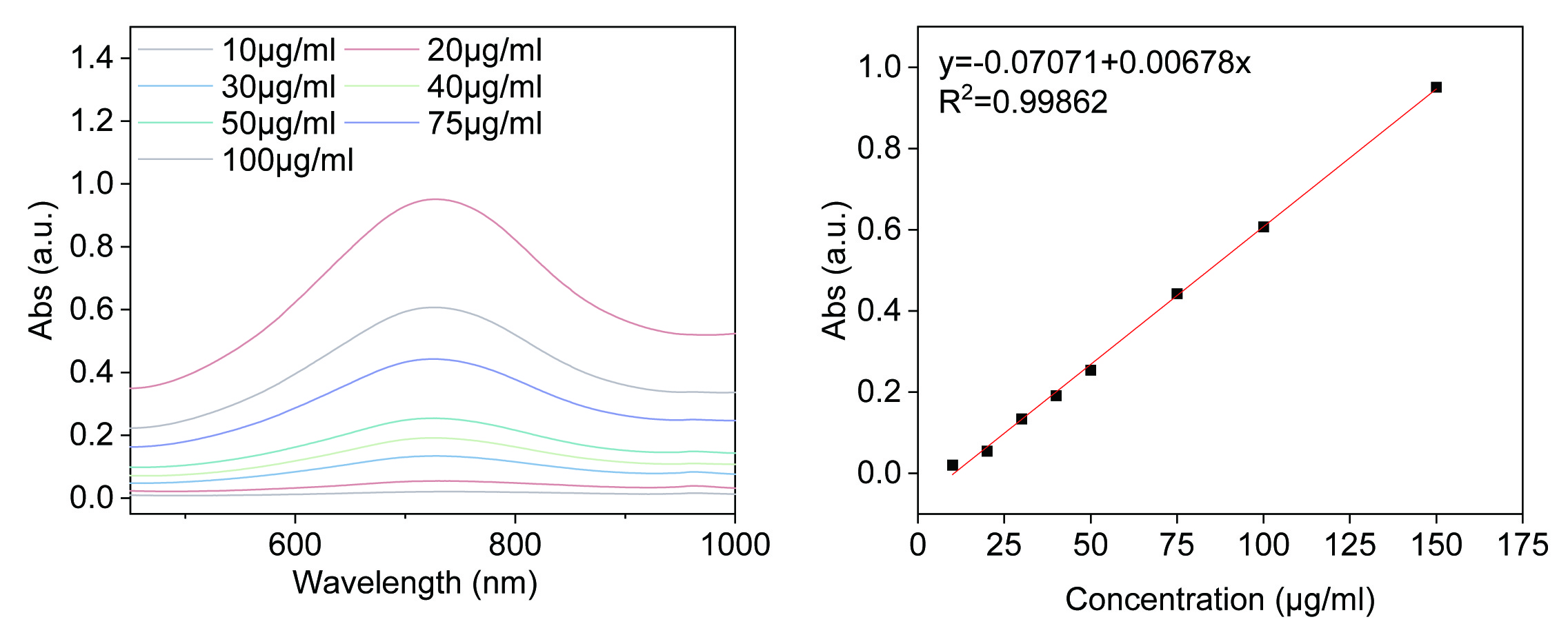


Figure S3: UV spectra of MnPOM at different concentrations and their standard curve.


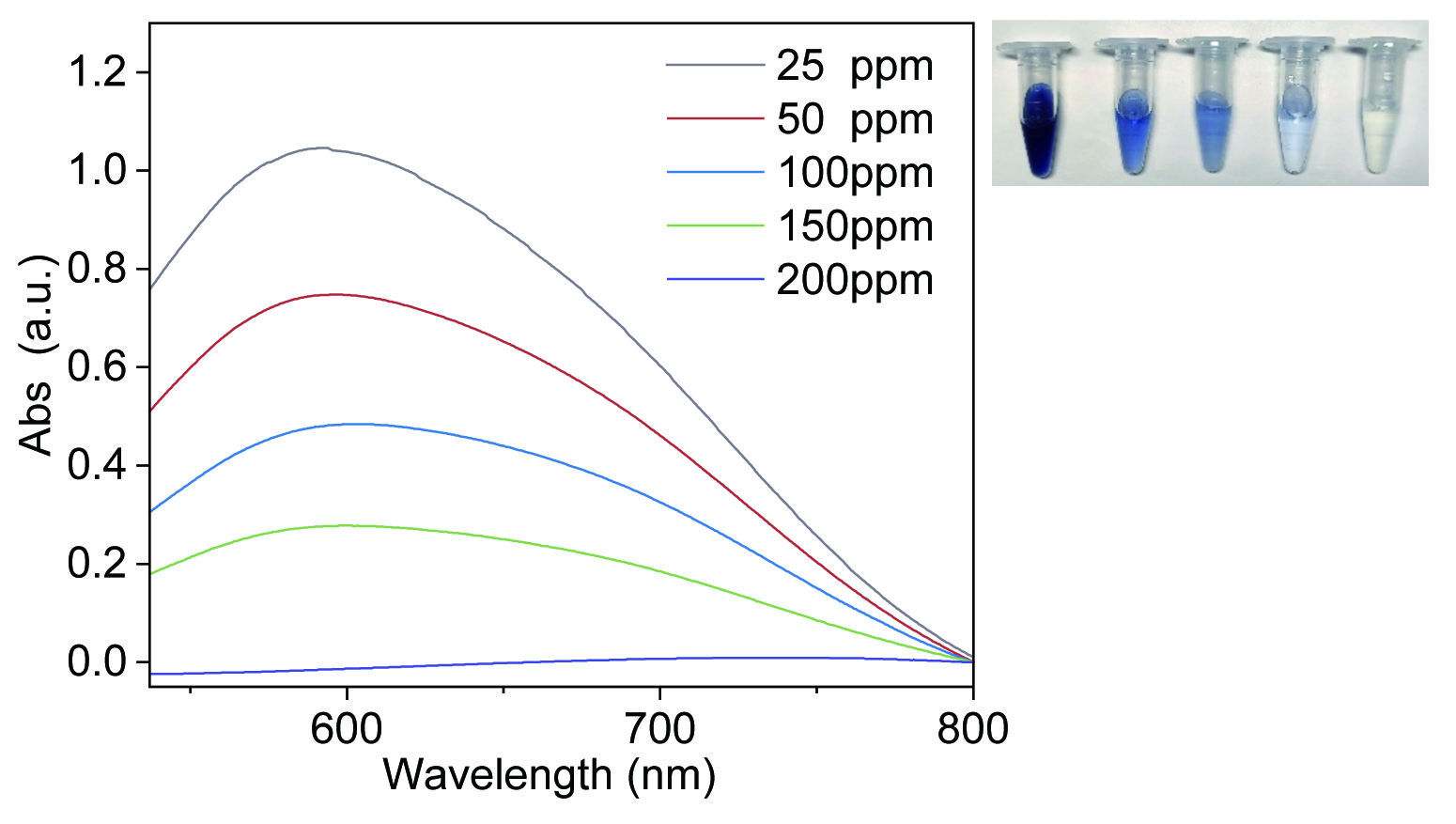


Figure S4: UV spectra of different concentrations of H_2_O_2_ by potassium iodide iodine blue spectrophotometric method.


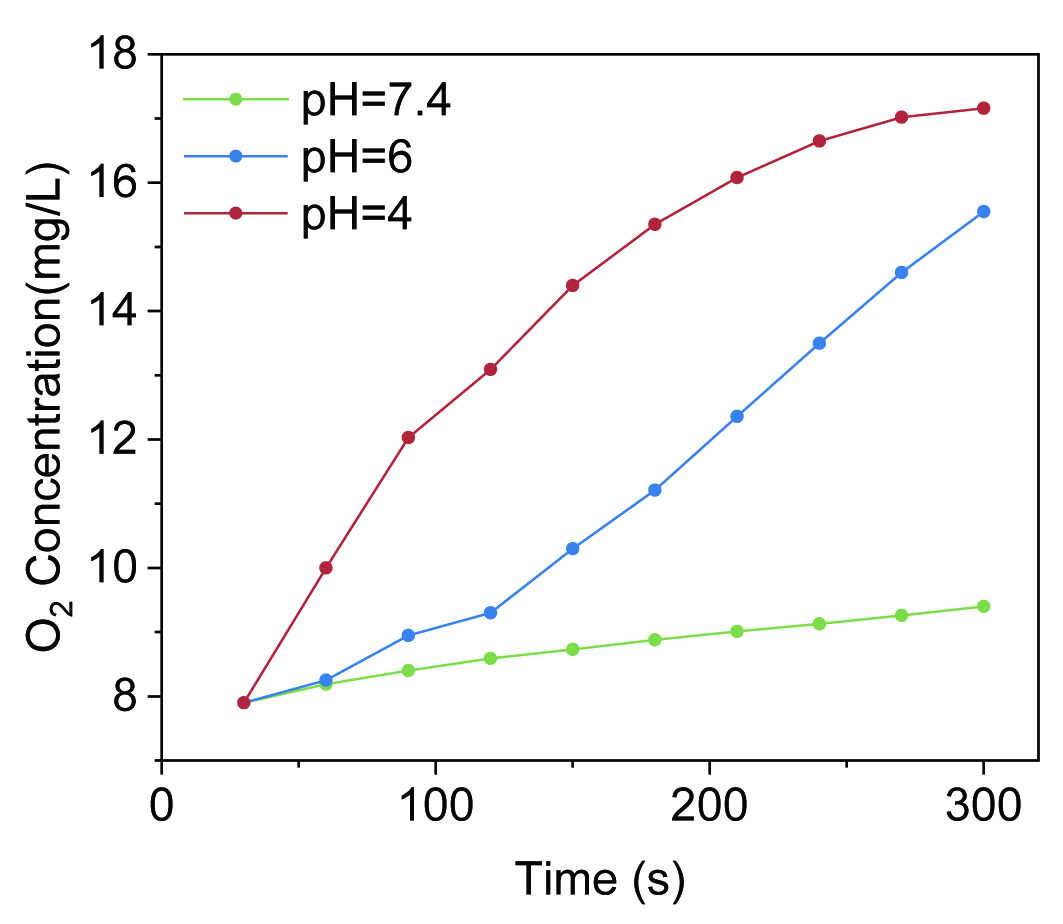


Figure S5: Oxygen evolution curves of MnPOM at different pH values.


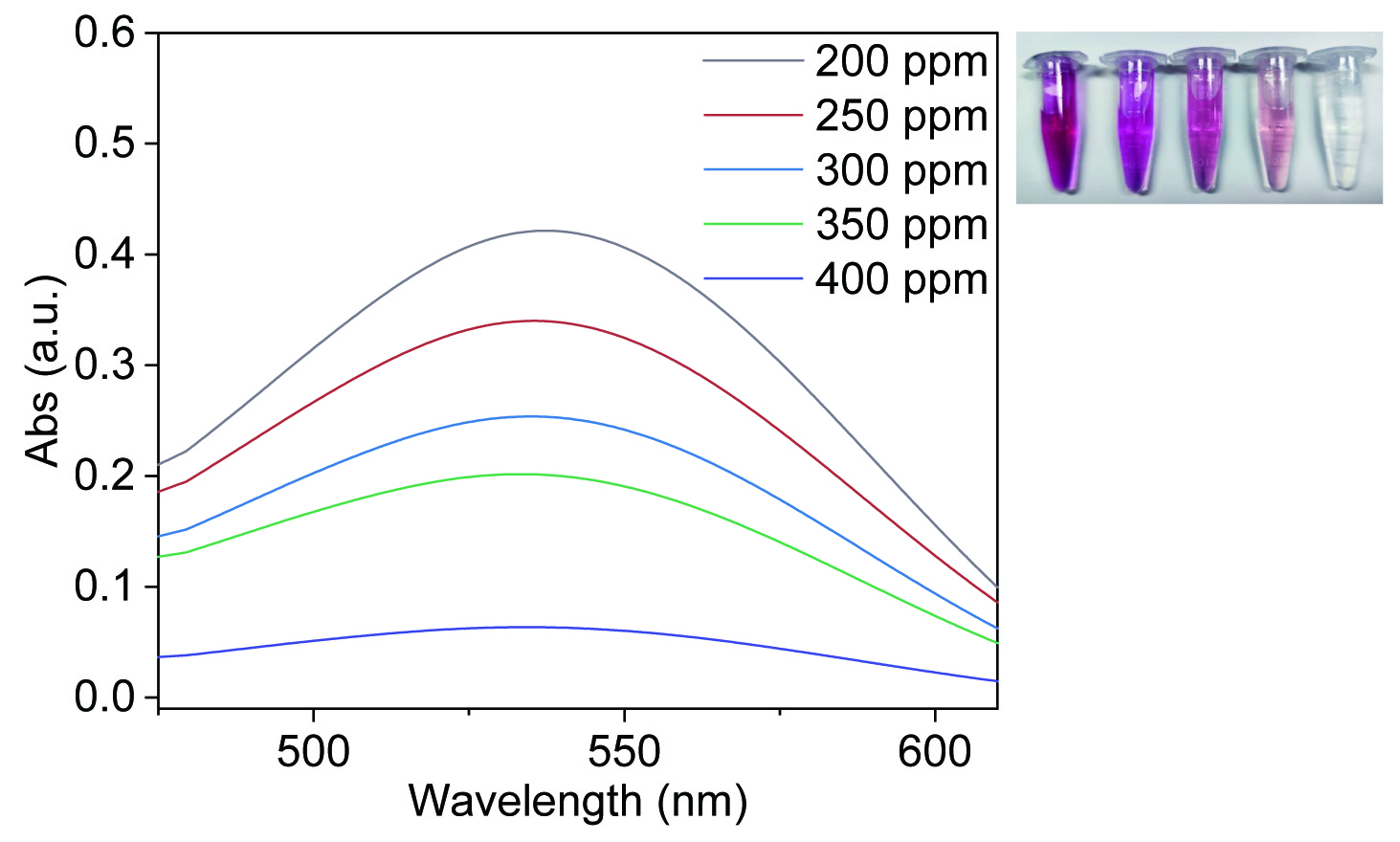


Figure S6: UV spectral curves of SOD activity in scavenging at varying MnPOM concentrations.


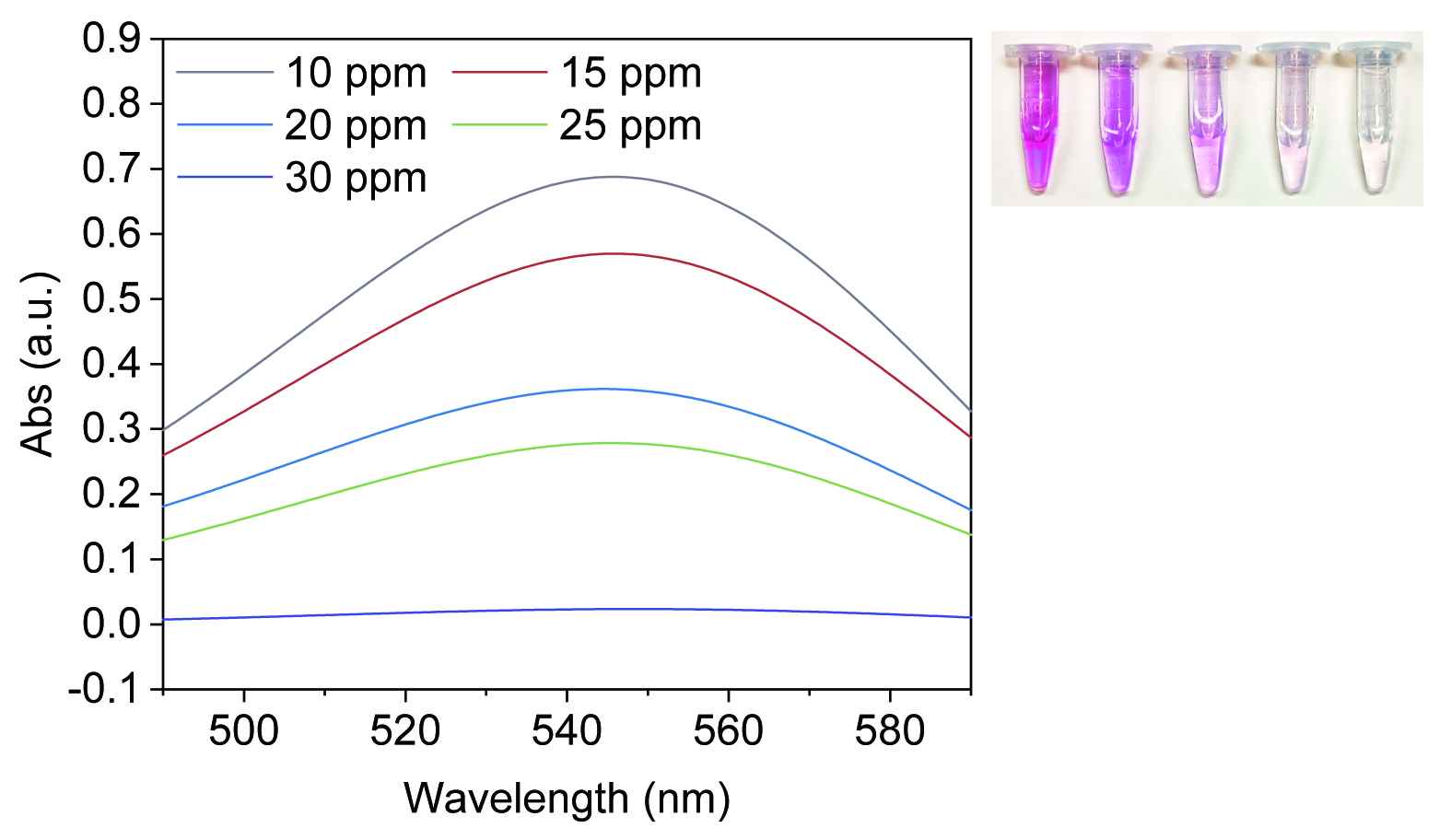


Figure S7: UV spectral curves of -OH scavenging activity at varying MnPOM concentrations.

Figure S8: Synthetic pathway for the preparation of DSPE-PEG2000-Dcpep.


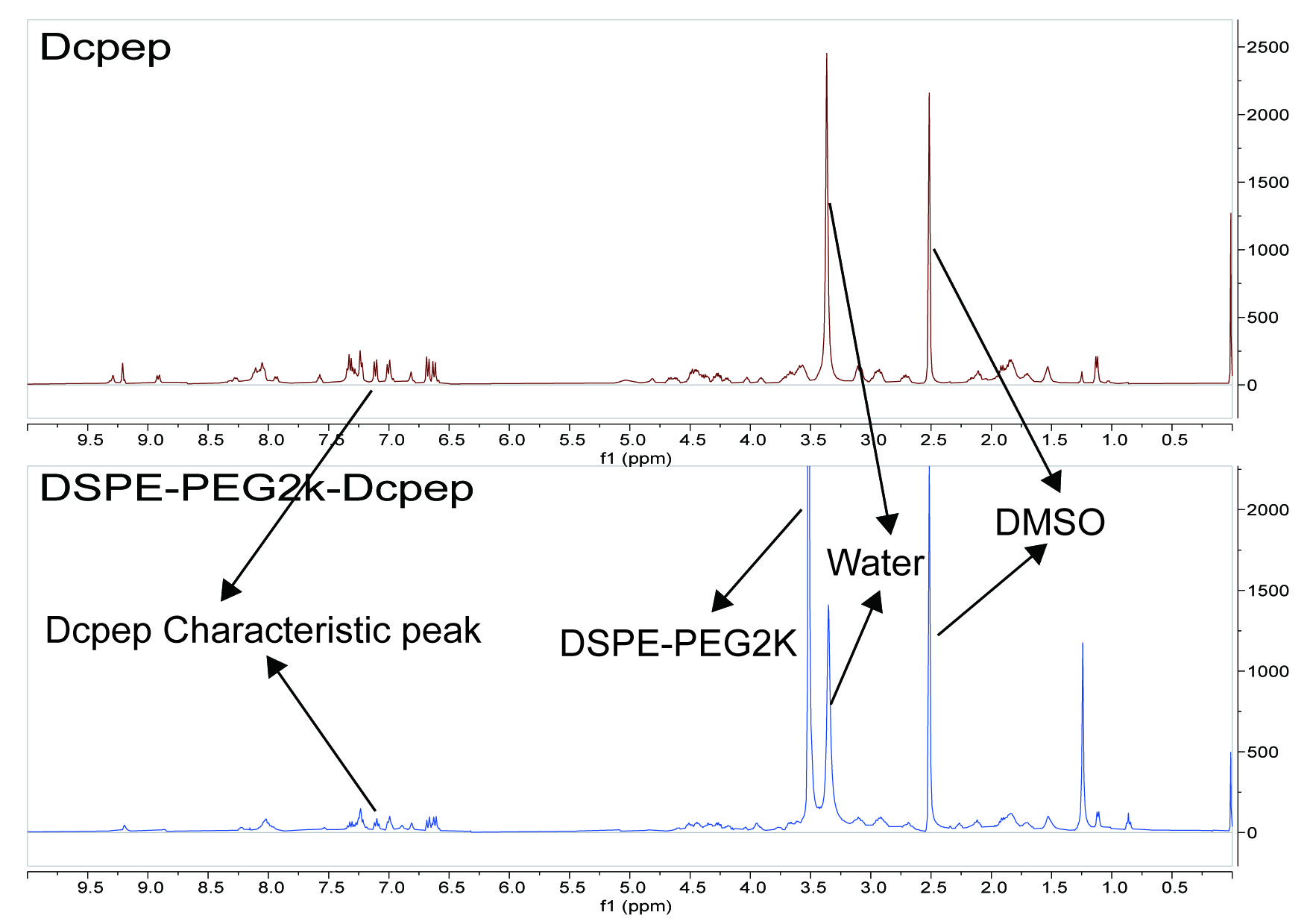


Figure S9: Proton NMR spectra of Dcpep and DSPE-PEG2000-Dcpep.

Figure S10: HPLC analysis of DSPE-PEG2000-Dcpep.


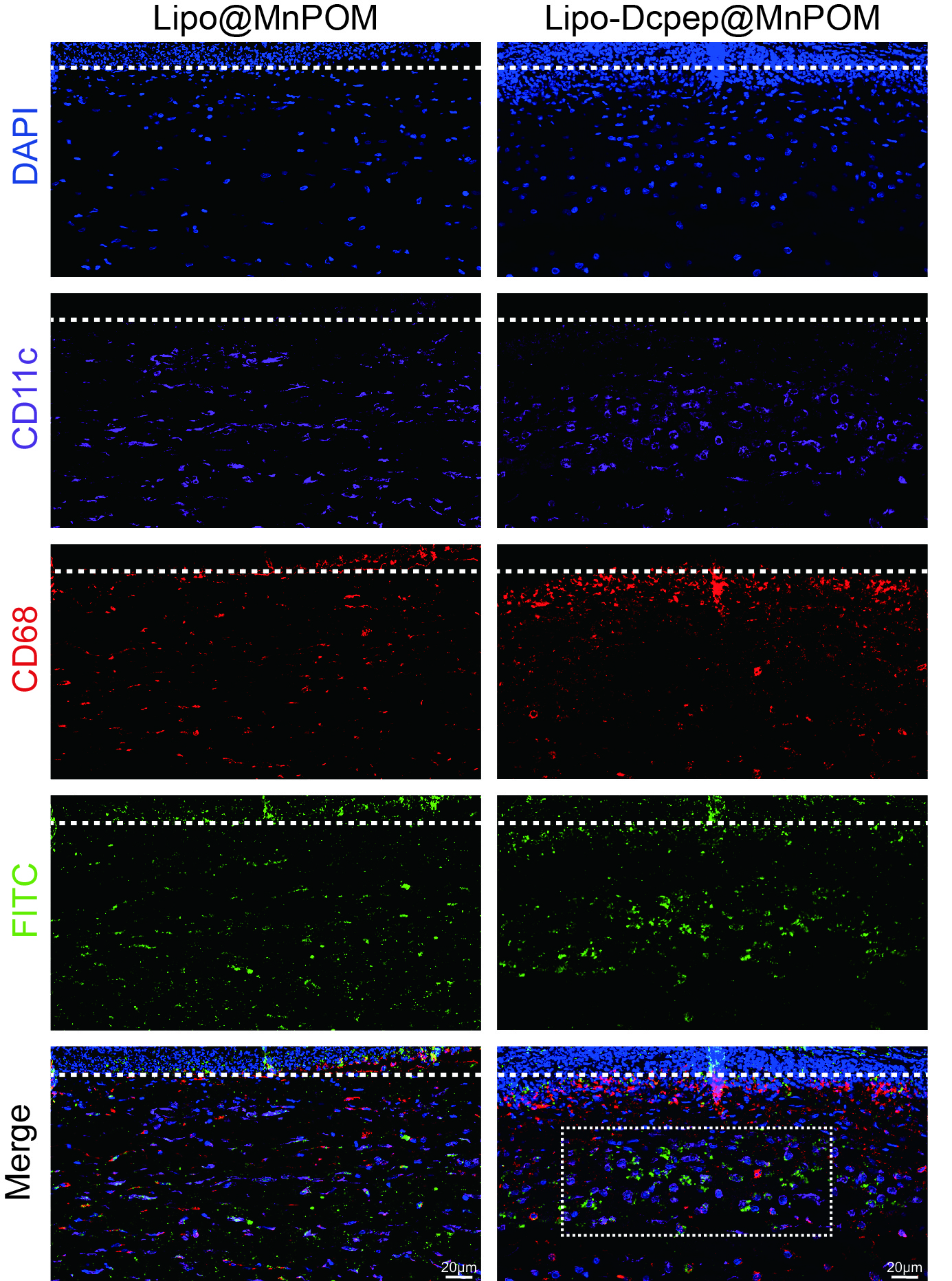


Figure S11. Immunofluorescence images of liposome distribution in different treatment groups. CD11c (purple), CD68 (red), FITC-labeled liposomes (green), DAPI (blue). Scale bar: 20 μm.


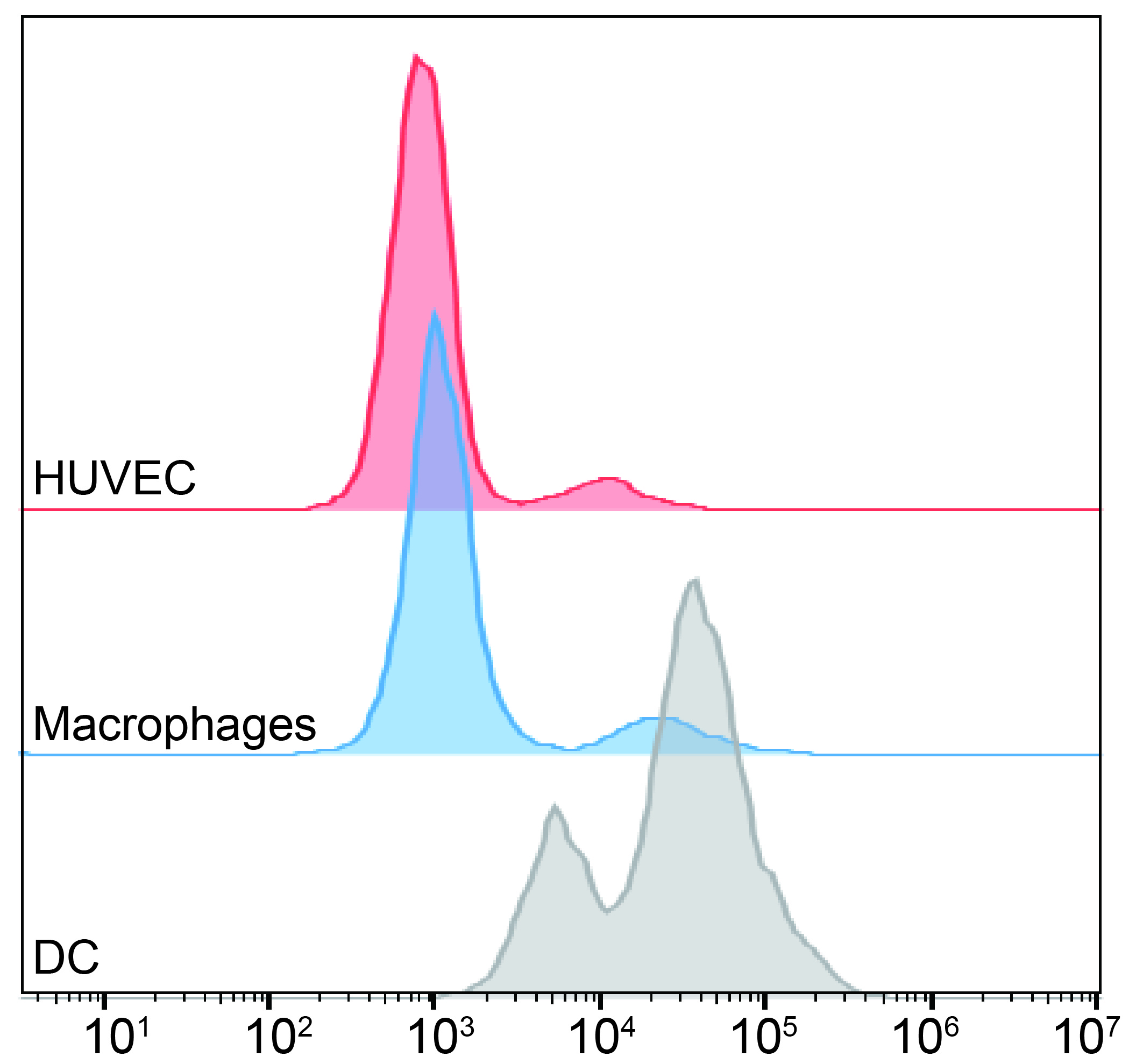


Figure S12. Flow cytometry analysis of intracellular uptake of Cy5.5-labeled liposomes.


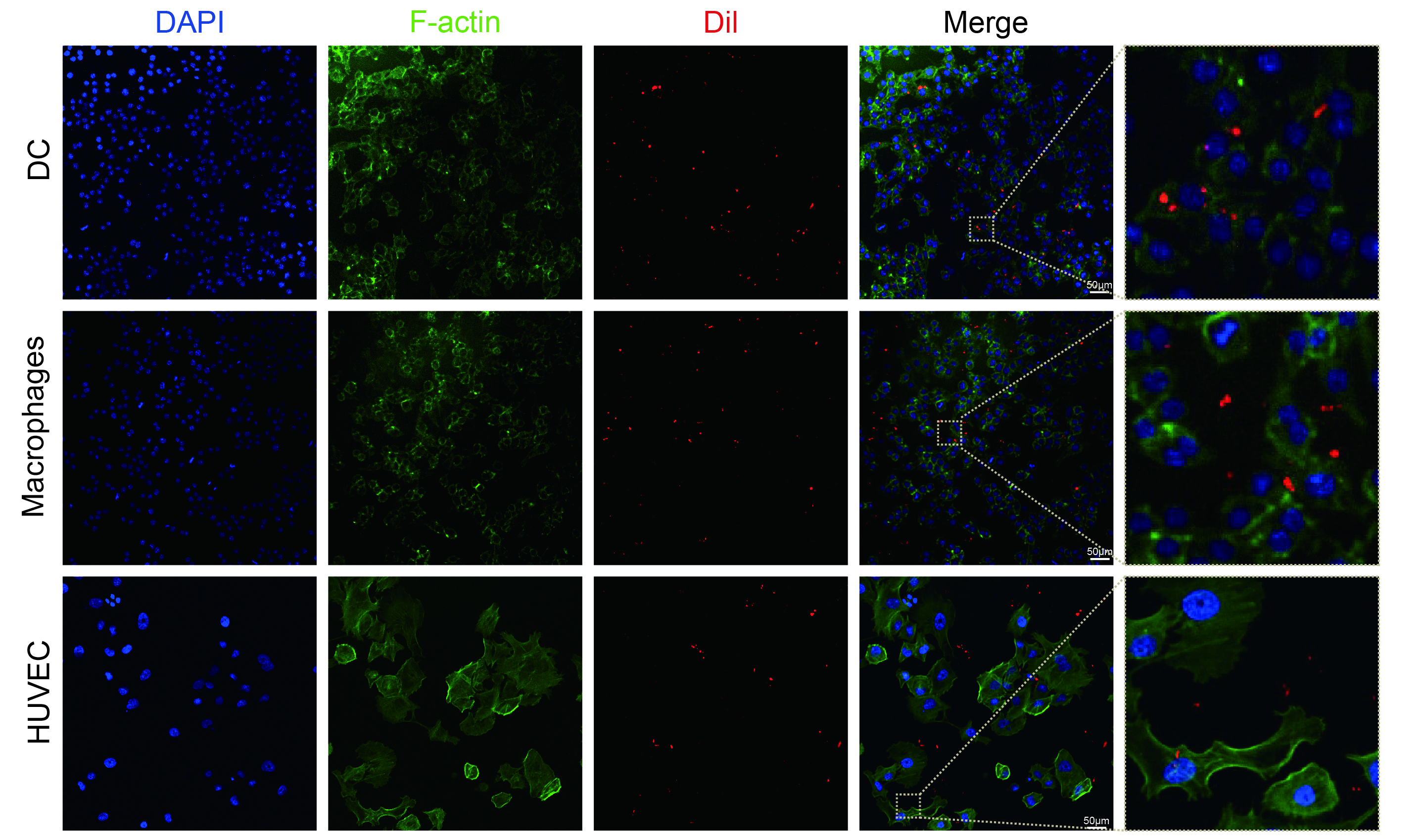


Figure S13. Immunofluorescence images of Lipo-Dcpep@MnPOM targeting different phagocytes. F-actin (green), Dil-labeled liposomes (red), DAPI (blue). Scale bar: 50 μm.


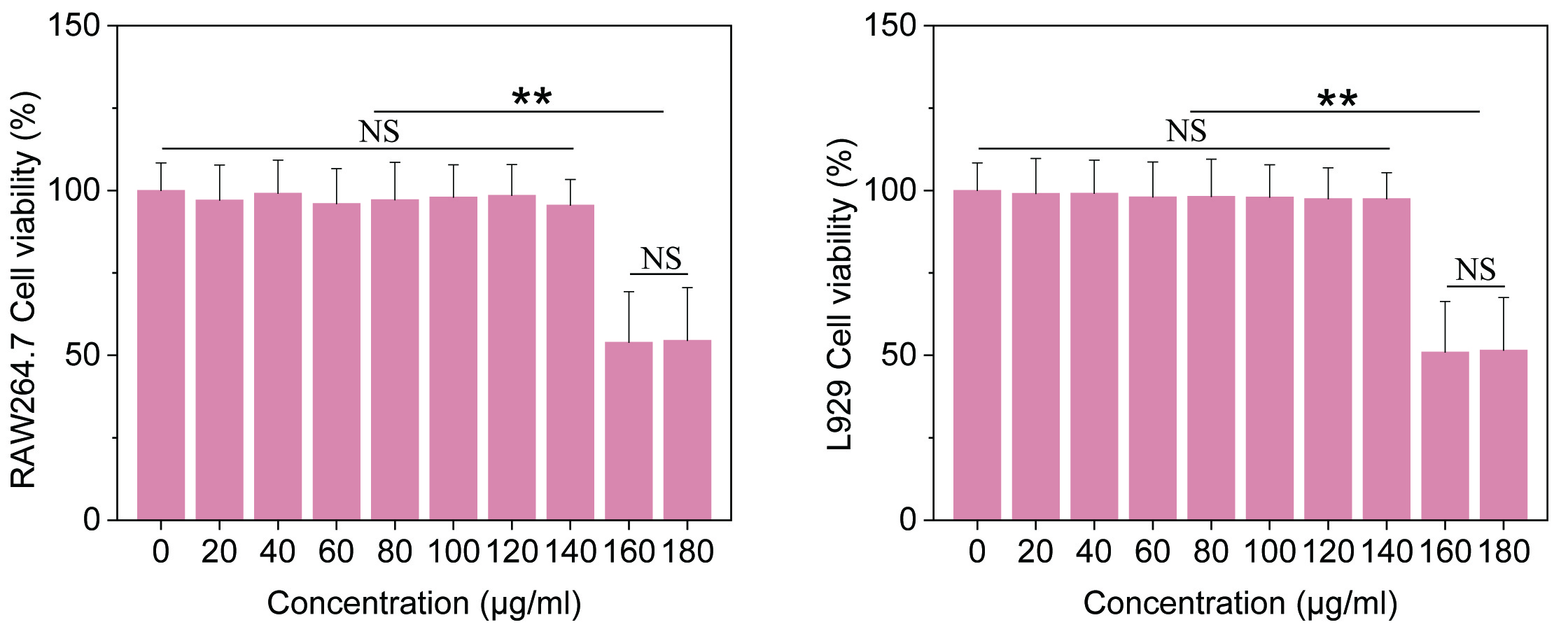


Figure S14: Cell viability of RAW264.7, and L929 cells at different concentrations of MnPOM. All values are presented as mean ± SD; *P ≤ 0.05; P ≥ 0.05 is considered non-significant (NS).


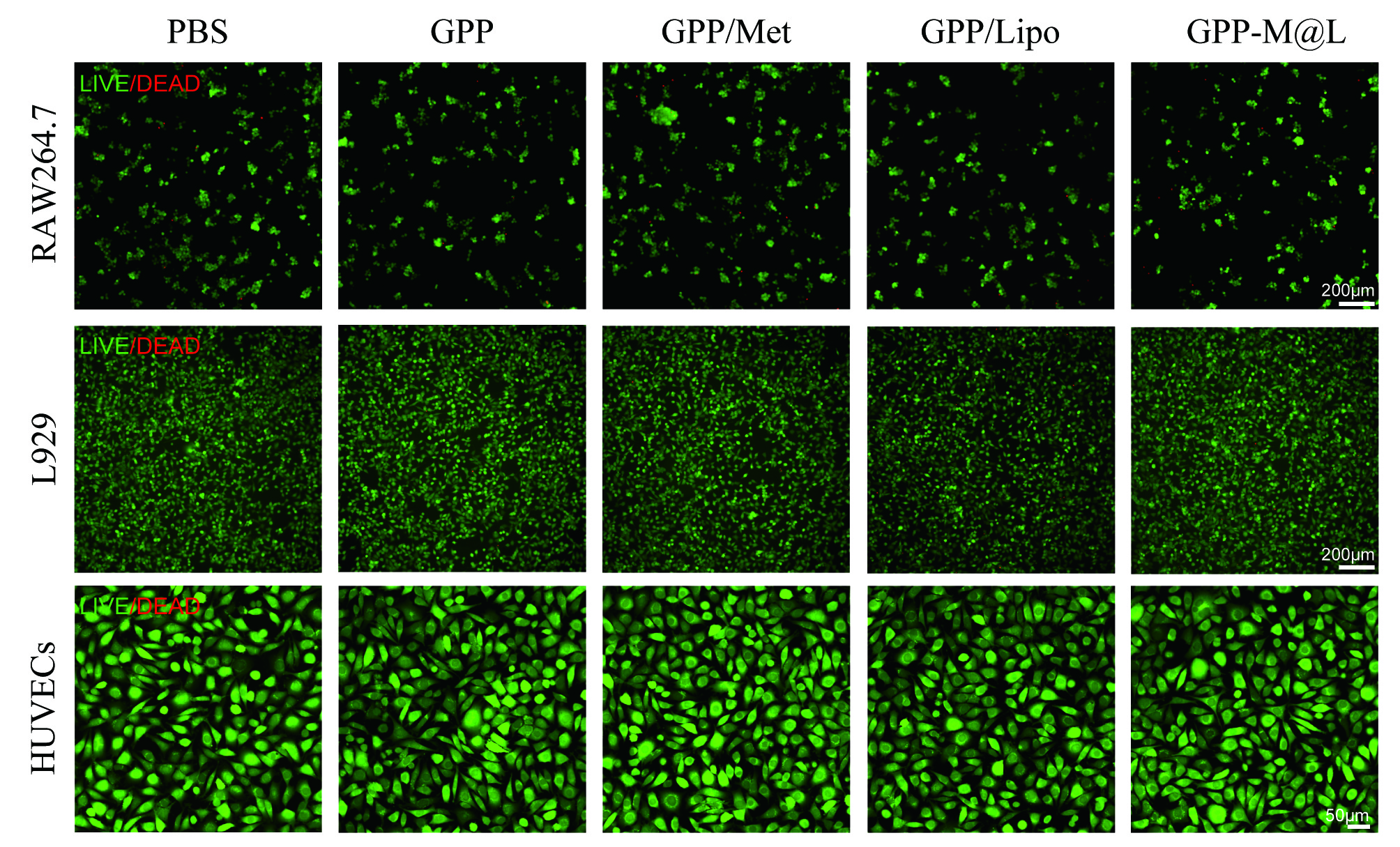


Figure S15: Live death staining of RAW264.7,HUVECs and L929 in different treatment groups. Scale bar:200 μm,50μm(PBS group represents normal glucose level)


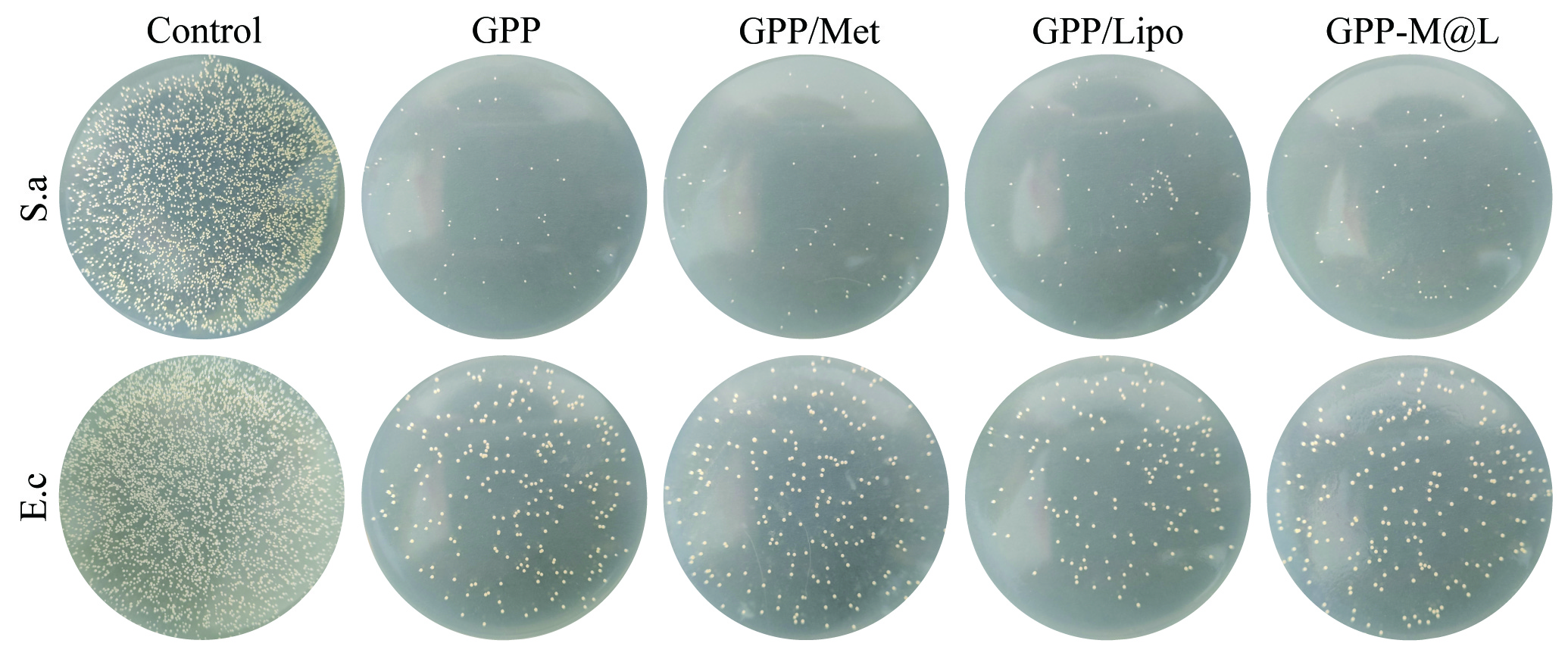


Figure S16: Co-culture of Staphylococcus aureus and Escherichia coli colonies in hydrogels from different groups.(Control group represents hyperglycemia)


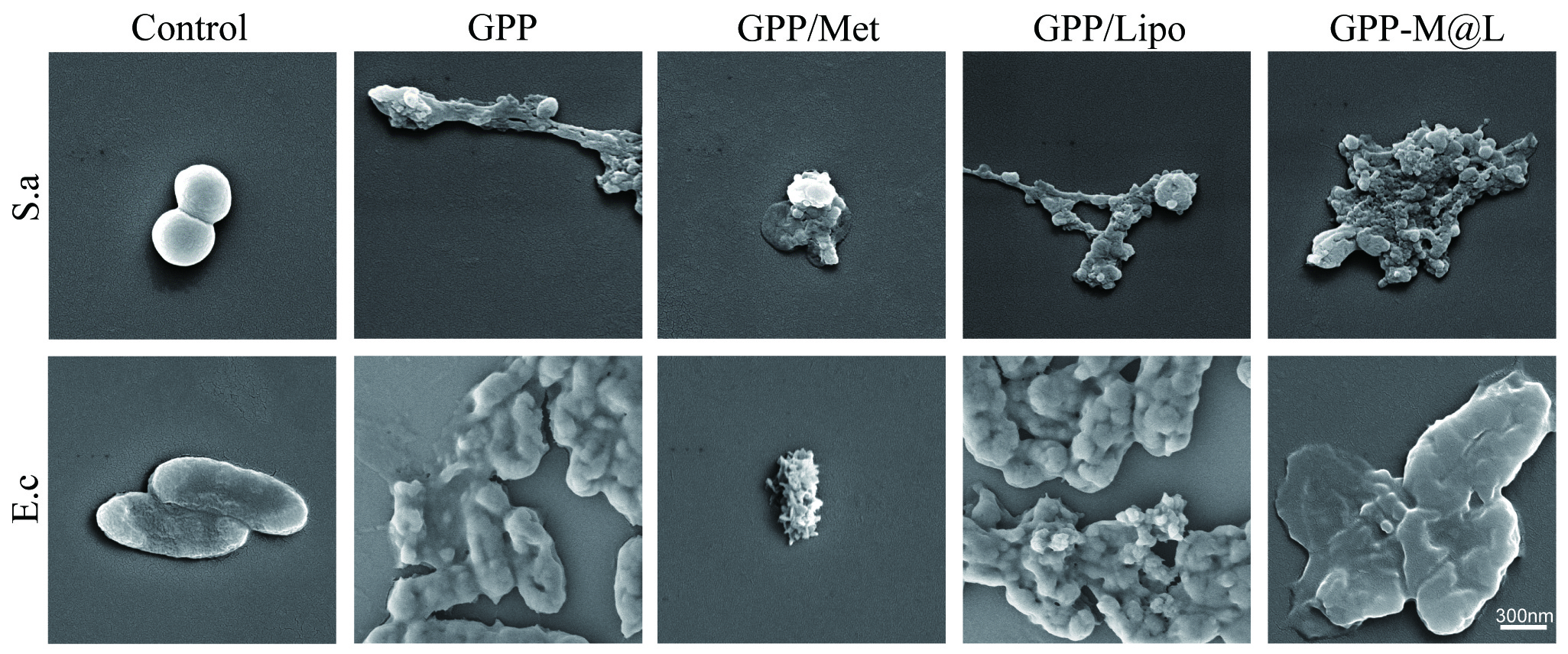


Figure S17: Scanning Electron Microscopy (SEM) images of Staphylococcus aureus and Escherichia coli co-cultured in hydrogels from various groups. Scale bar: 300 nm. (Control group represents hyperglycemia)


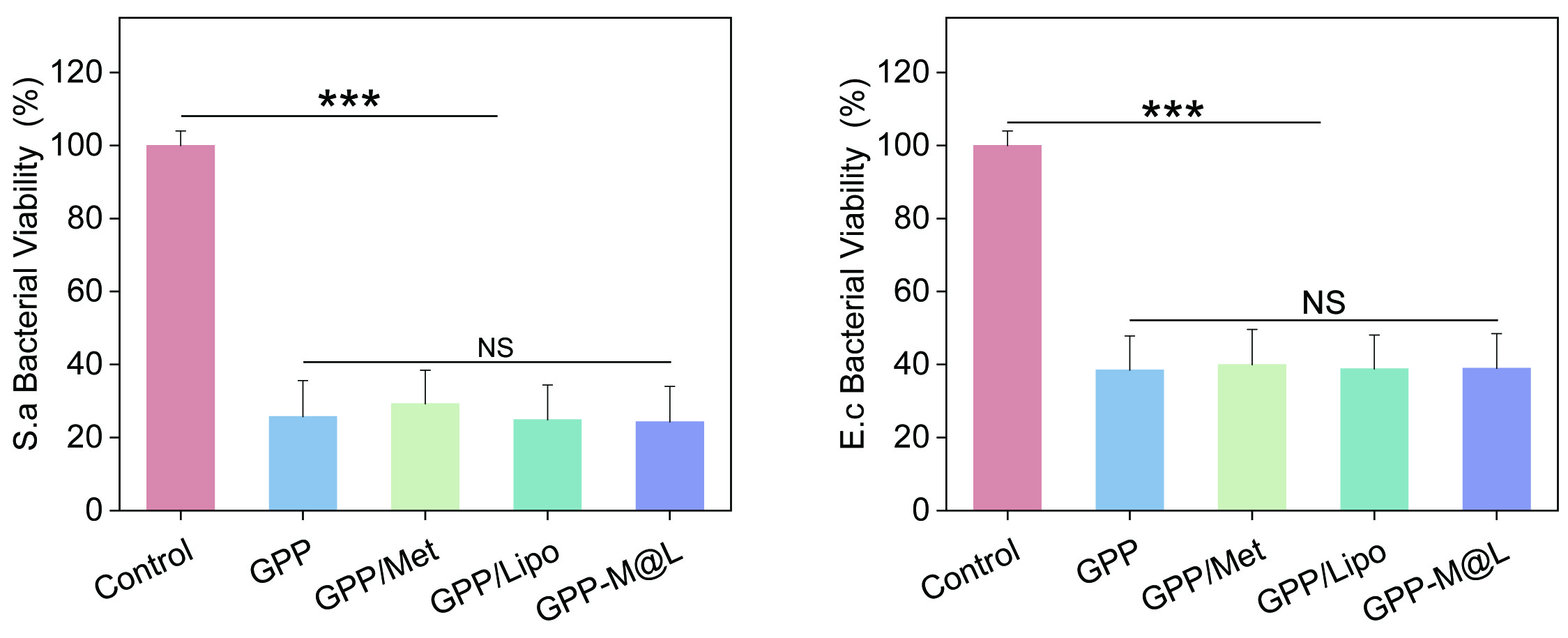


Figure S18: Bacterial viability of Staphylococcus aureus and Escherichia coli (quantified in Fig. S17). All values are presented as mean ± SD; *P ≤ 0.05; P ≥ 0.05 is considered non-significant (NS).


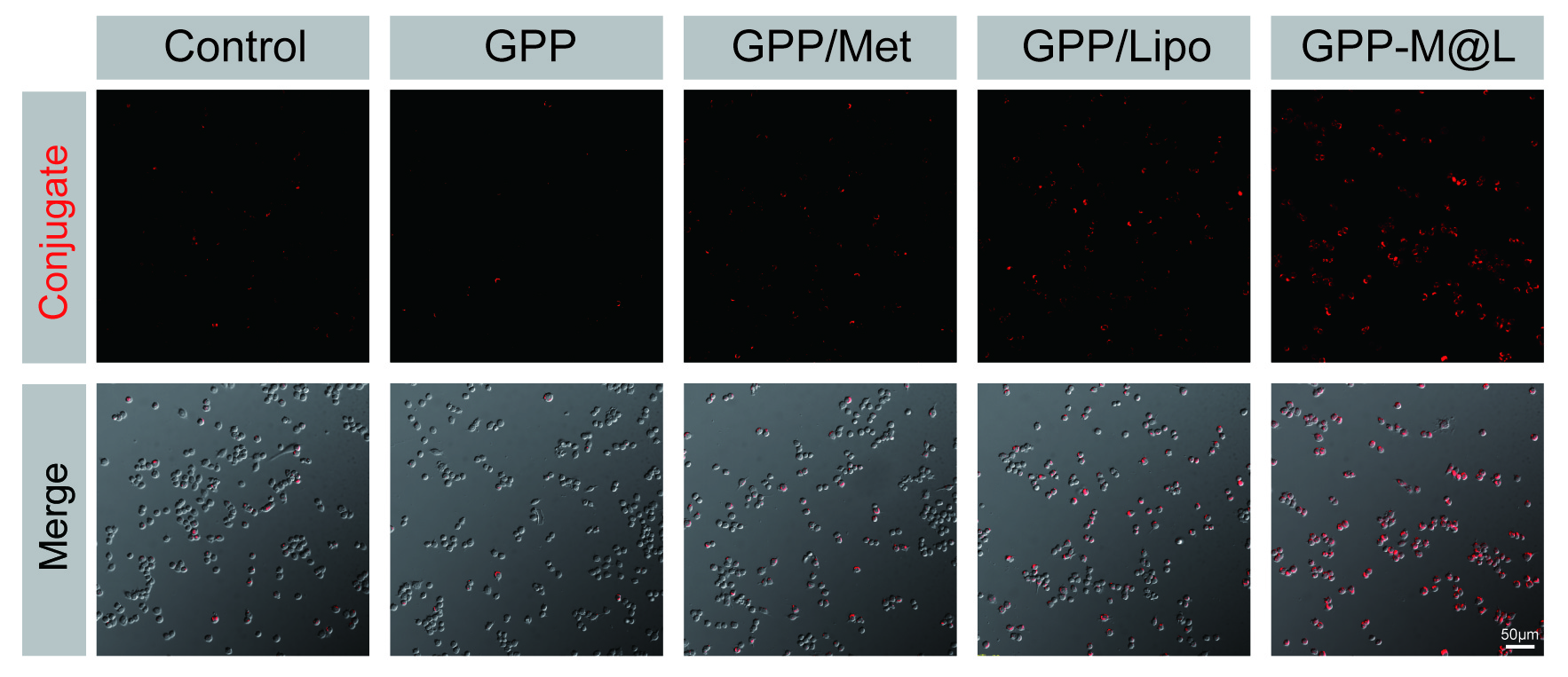


Figure S19: Efferocytosis of DCs following treatment with different hydrogel groups as detected by Conjugate product reaction. Scale bar: 50 μm.(Control group represents hyperglycemia)


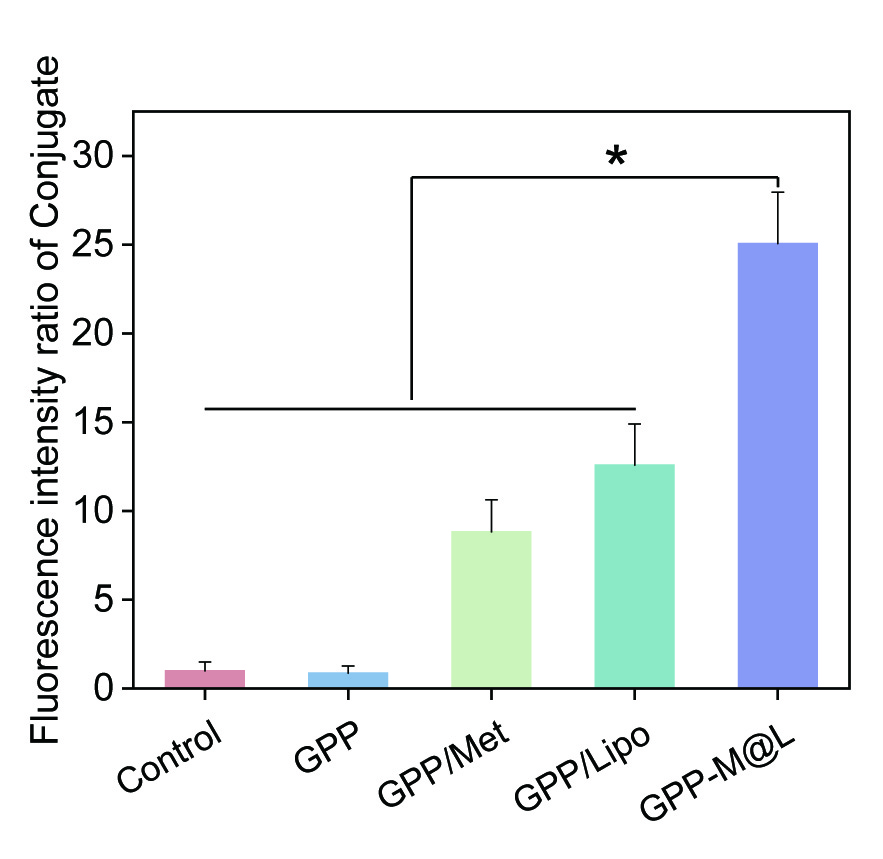


Figure S20: Quantitative analysis of Conjugate expression from Figure S19. All values are presented as mean ± SD; *P ≤ 0.05; P ≥ 0.05 is considered non-significant (NS)


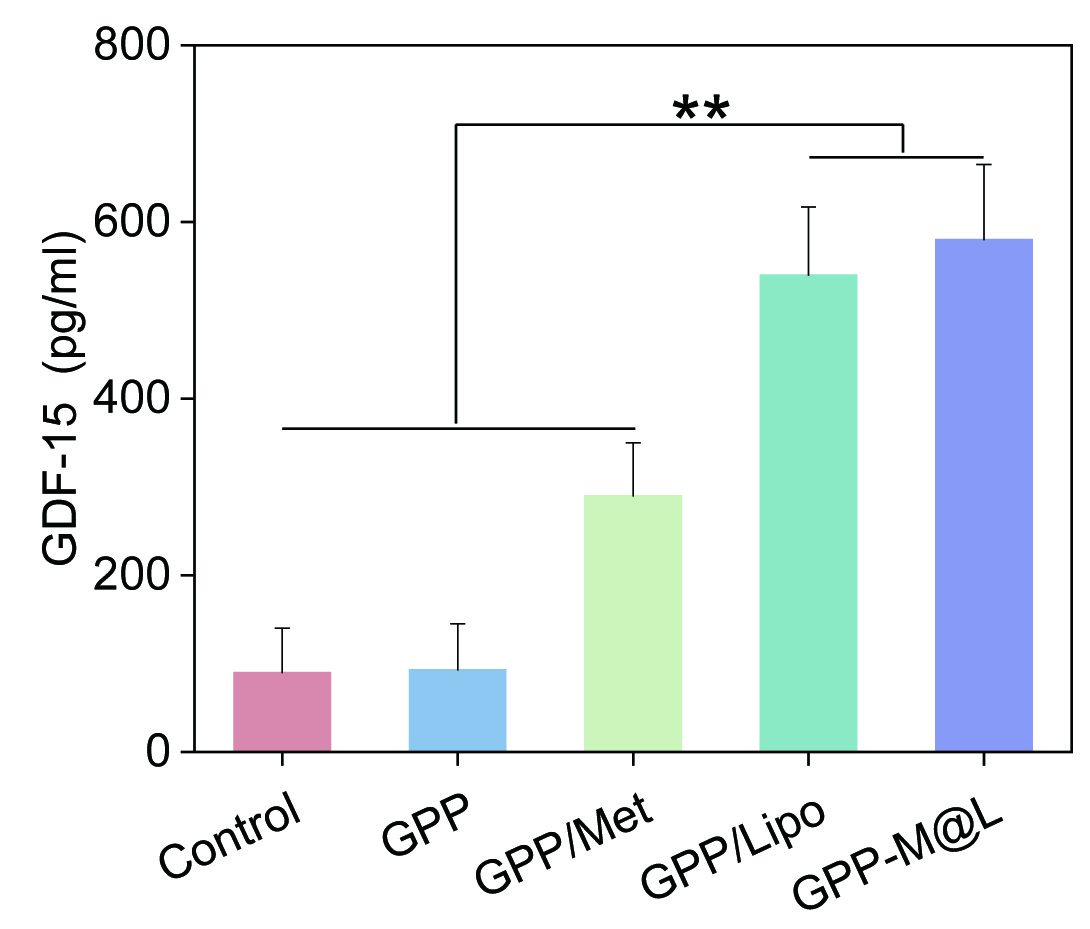


Figure S21: GDF15 levels in different groups. All values are presented as mean ± SD; *P ≤ 0.05; P ≥ 0.05 is considered non-significant (NS). (Control group represents hyperglycemia)


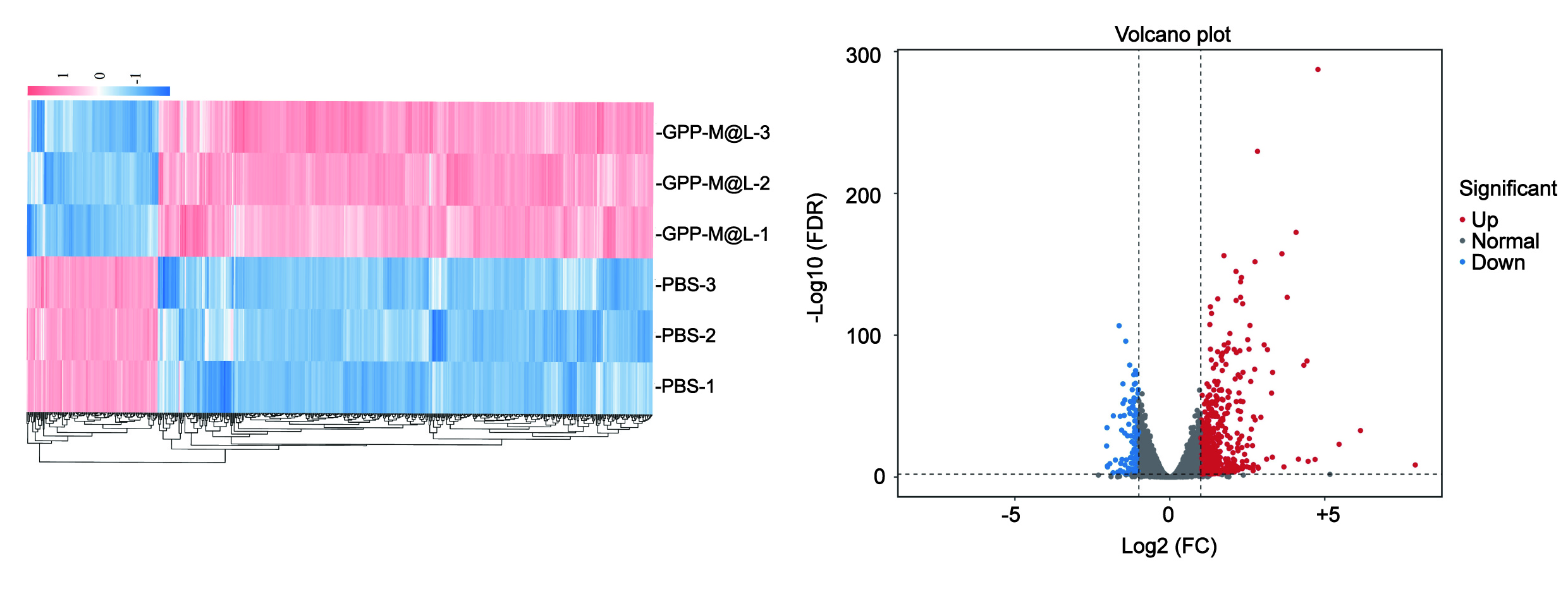


Figure S22: Heatmap of differential gene expression in the first stage (red indicates genes with relatively high expression levels; blue indicates genes with low expression levels) and volcano plot of differential gene expression (gray represents genes with no significant difference; red represents upregulated genes; blue represents downregulated genes).


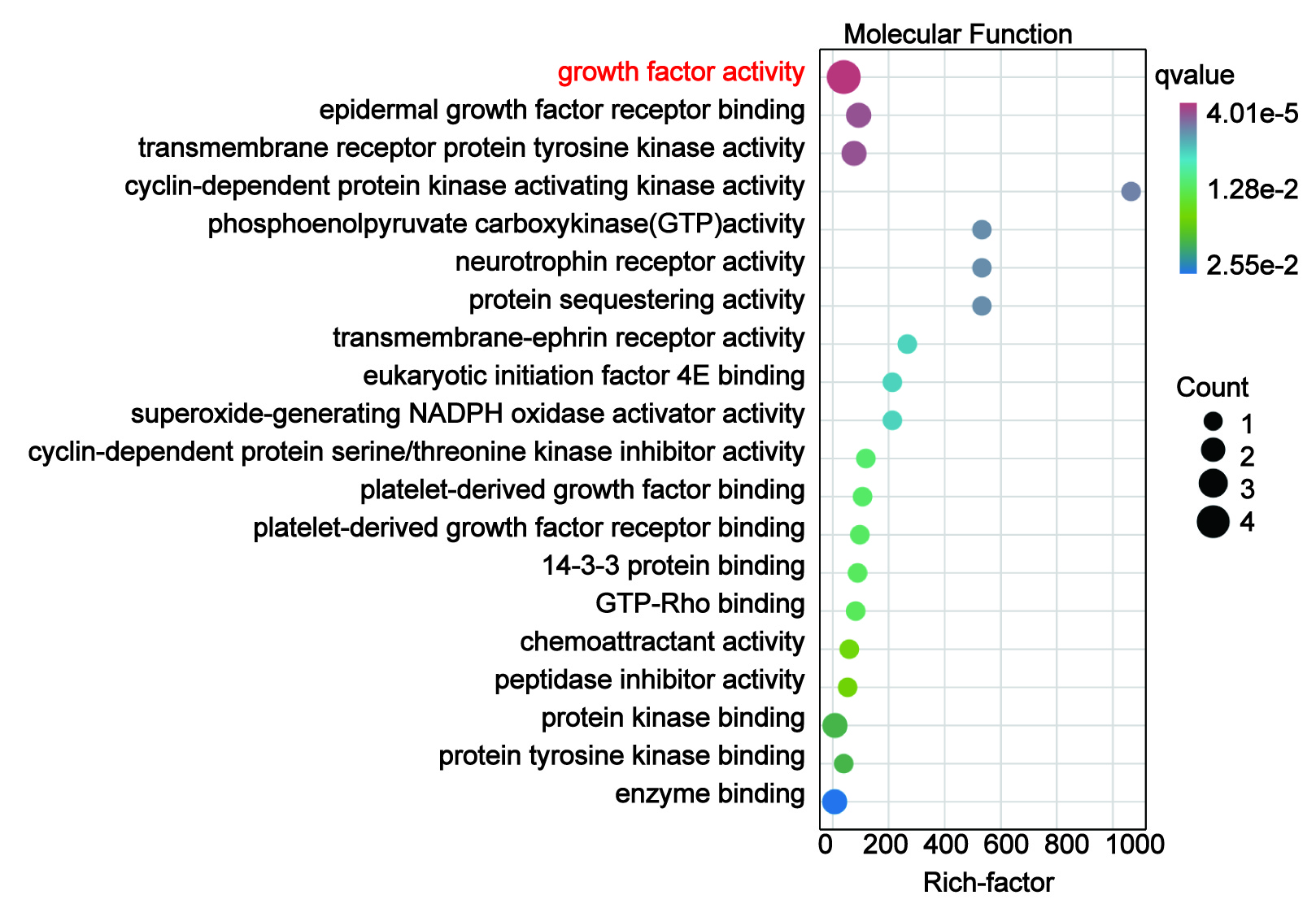


Figure S23: Molecular function analysis of GO enrichment for upregulated differential genes in the first section.


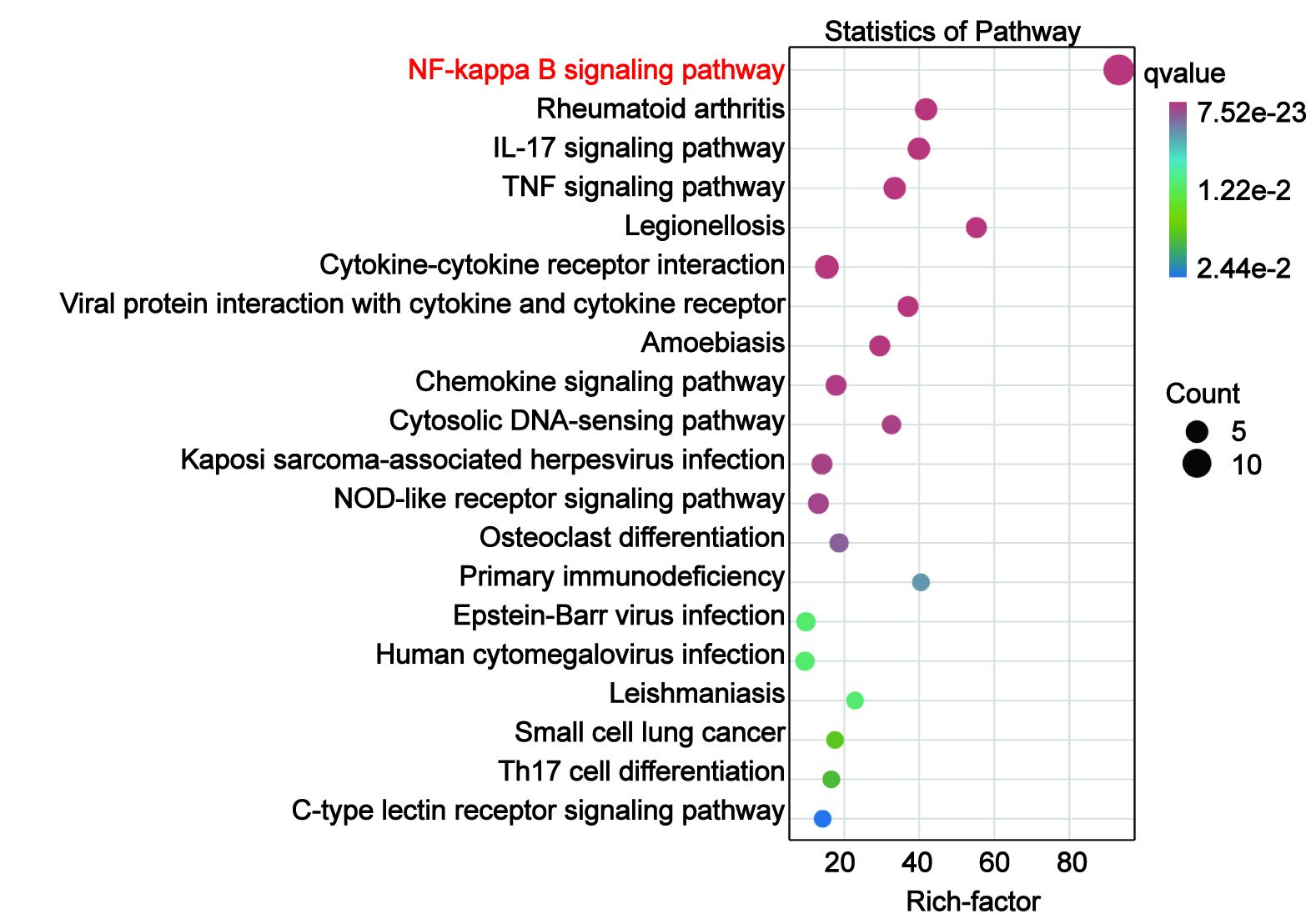


Figure S24: KEGG pathway enrichment analysis of downregulated differential genes in the first stage.


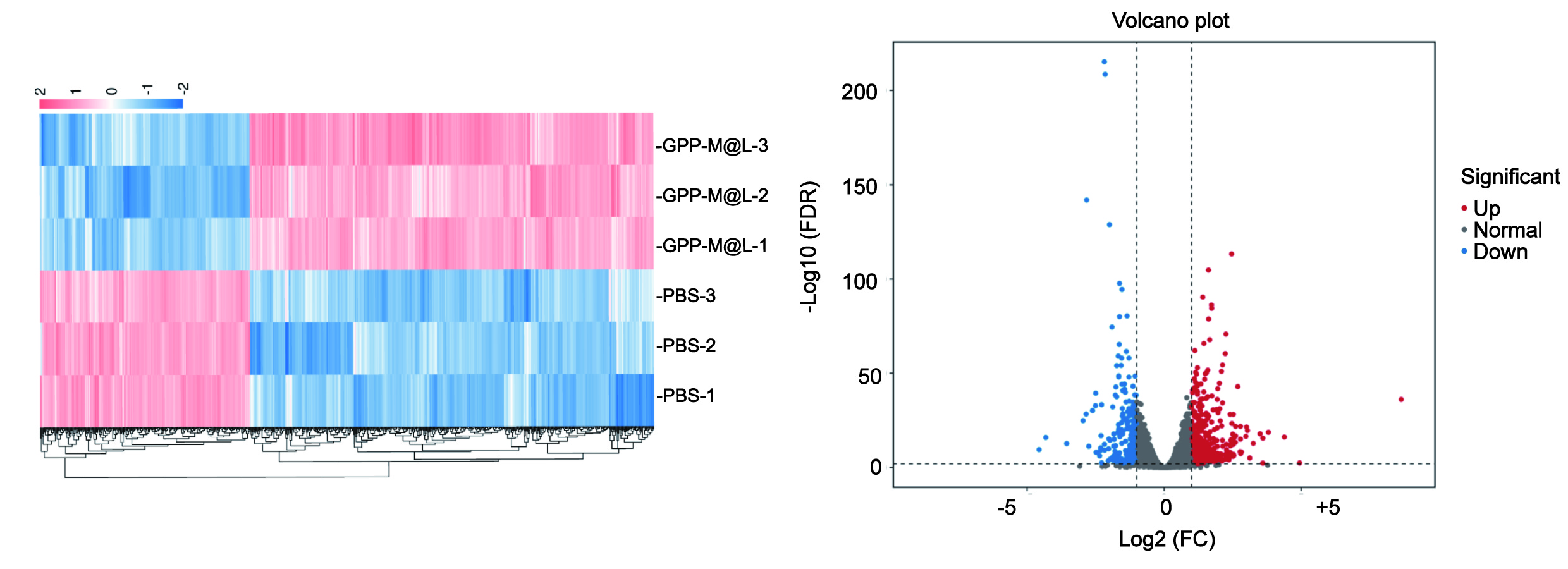


Figure S25: Heatmap of differential gene expression in the second stage (red: genes with relatively high expression levels; blue: genes with relatively low expression levels) and volcano plot of differential gene expression (gray: genes with no significant difference; red: upregulated genes; blue: downregulated genes).


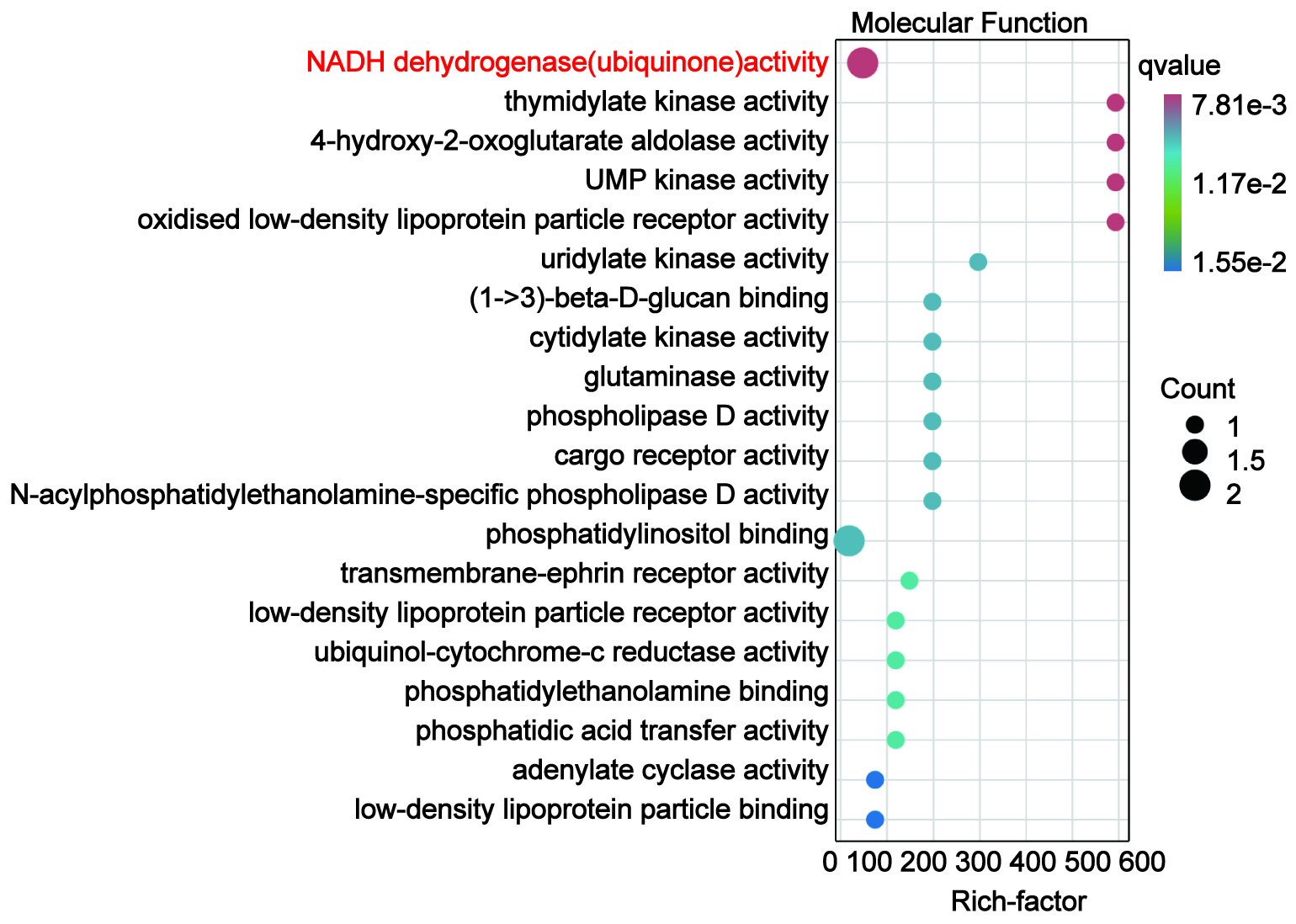


Figure S26: Molecular function analysis of GO enrichment for upregulated differential genes in the second stage.


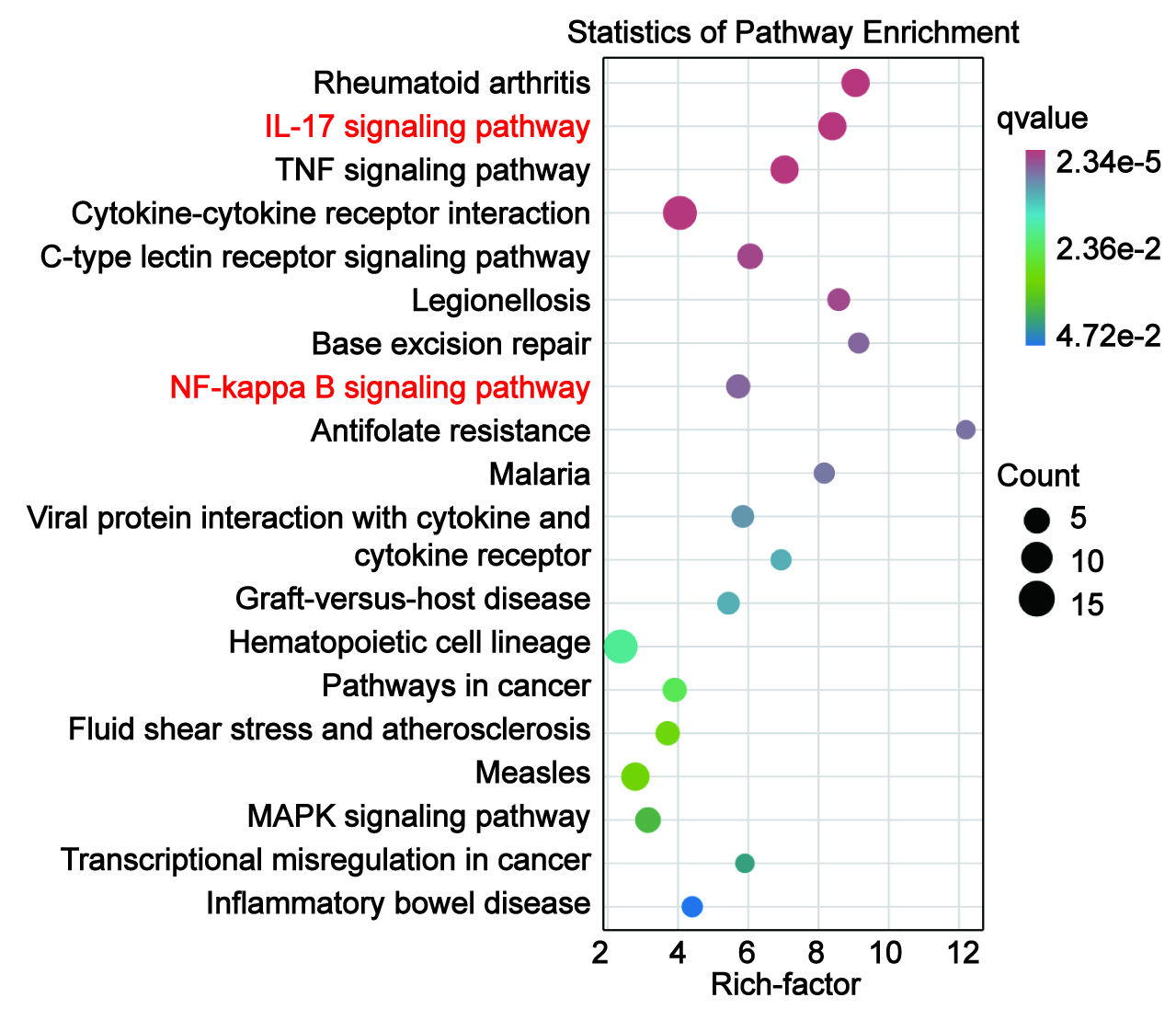


Figure S27: KEGG pathway enrichment analysis of downregulated differential genes in the second stage.


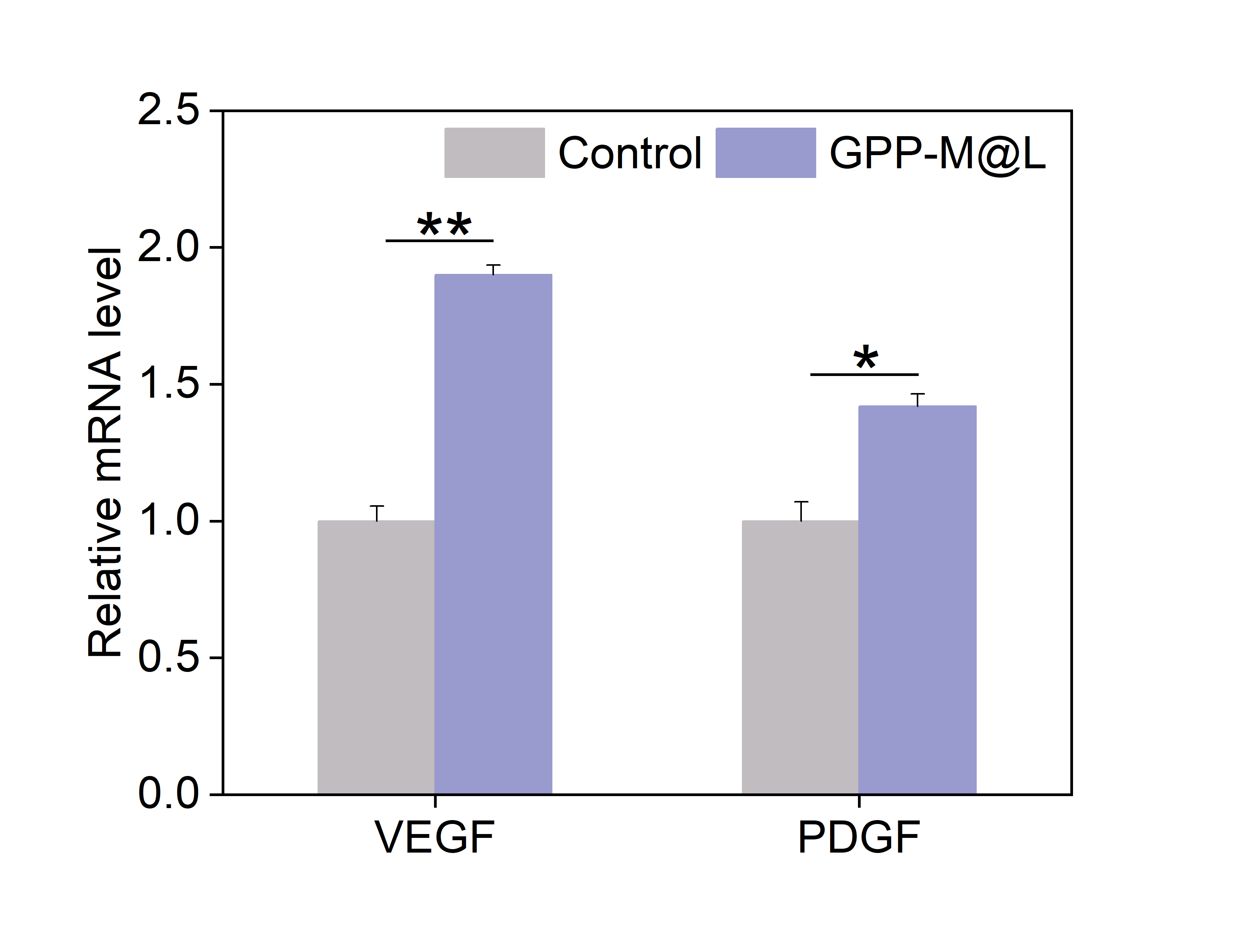


Figure S28: The relative mRNA expression level of VEGF and PDGF after treatment with GPP-M@L. All values are presented as mean ± SD; *P ≤ 0.05; P ≥ 0.05 is considered non-significant (NS).


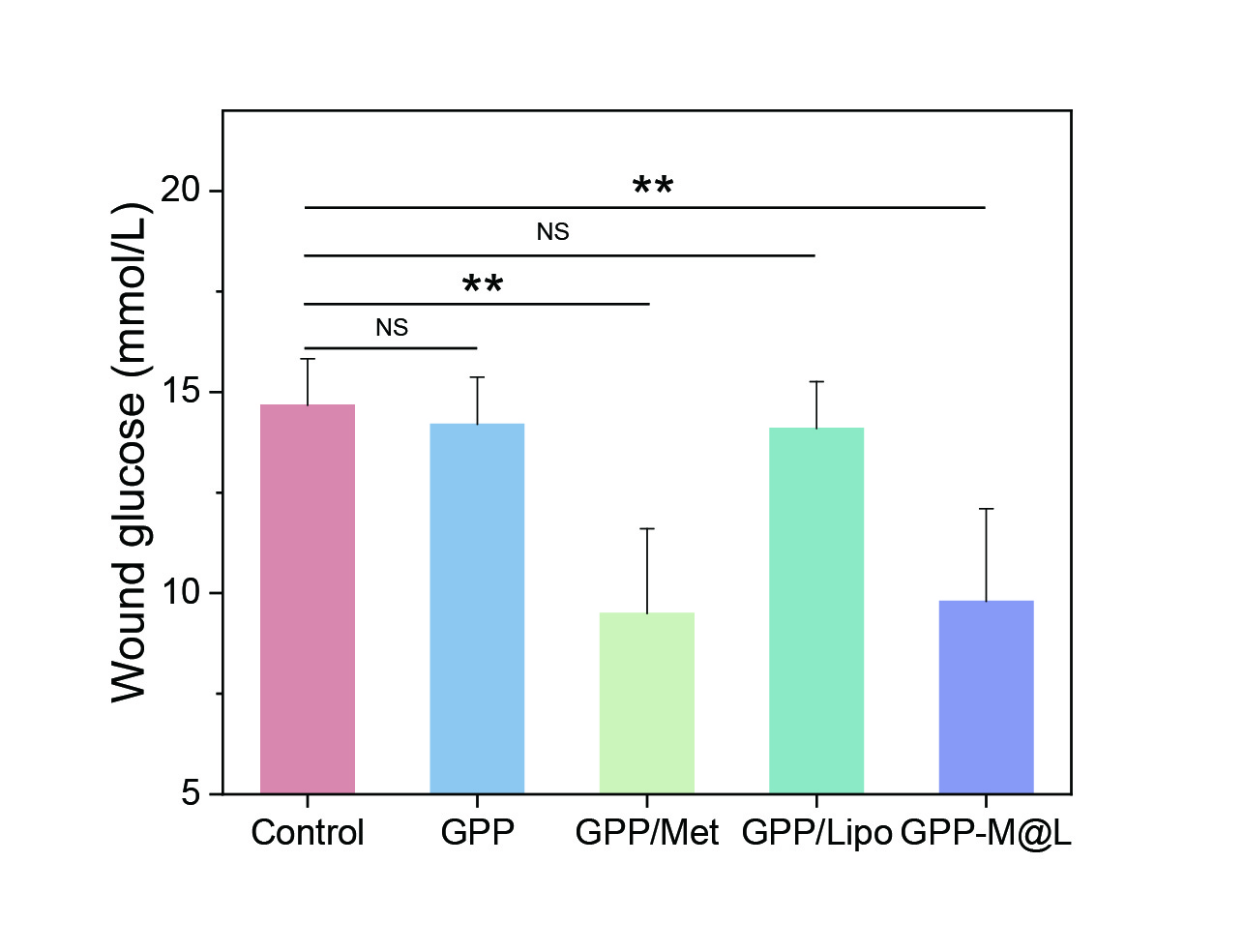


Figure S29. Glucose content in wounds at day 3 after different treatments. All values are presented as mean ± SD; *P ≤ 0.05; P ≥ 0.05 is considered non-significant (NS).


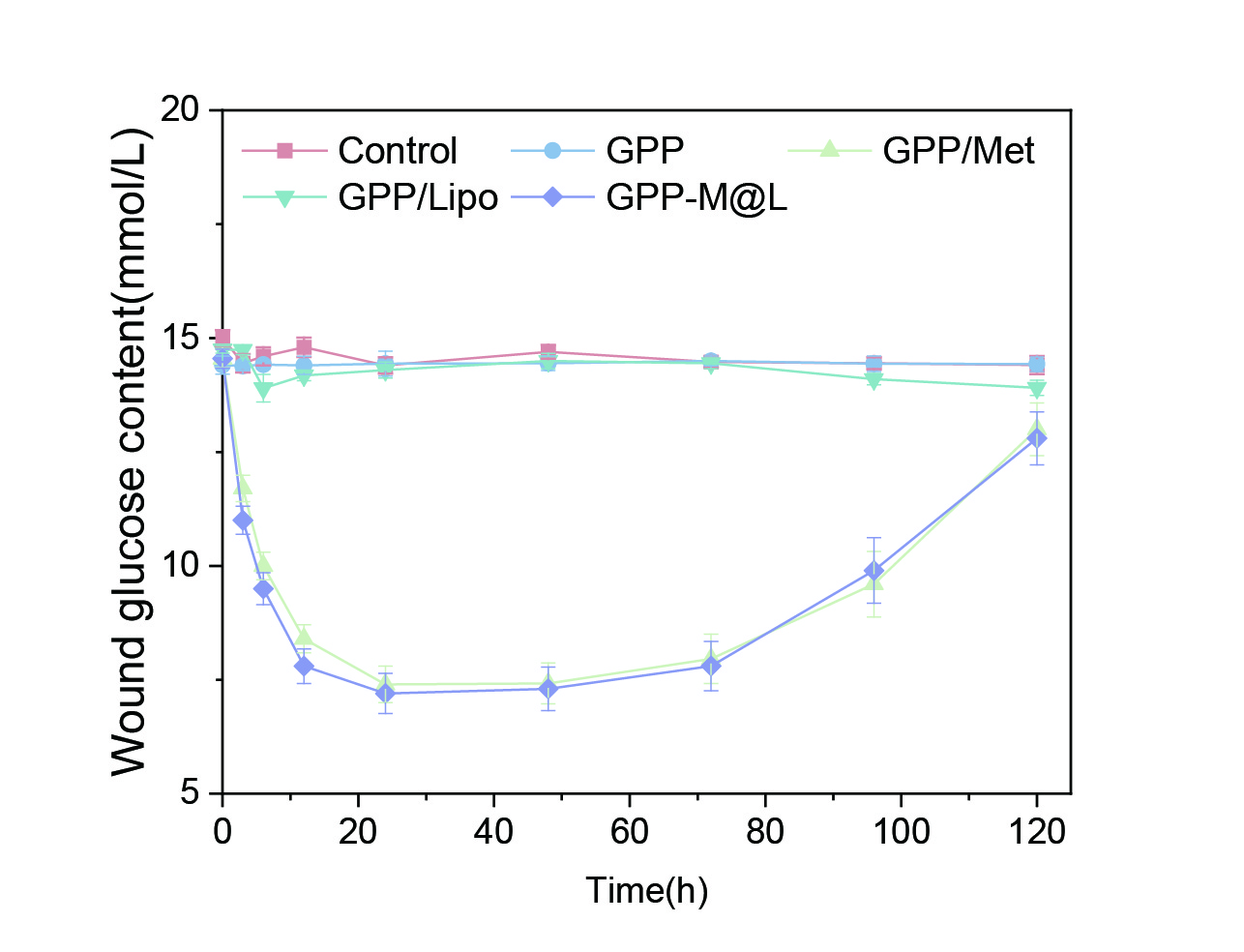


Figure S30. The changing trends in glucose concentration in wounds of mice with different treatments. All values are presented as mean ± SD; *P ≤ 0.05; P ≥ 0.05 is considered non-significant (NS).


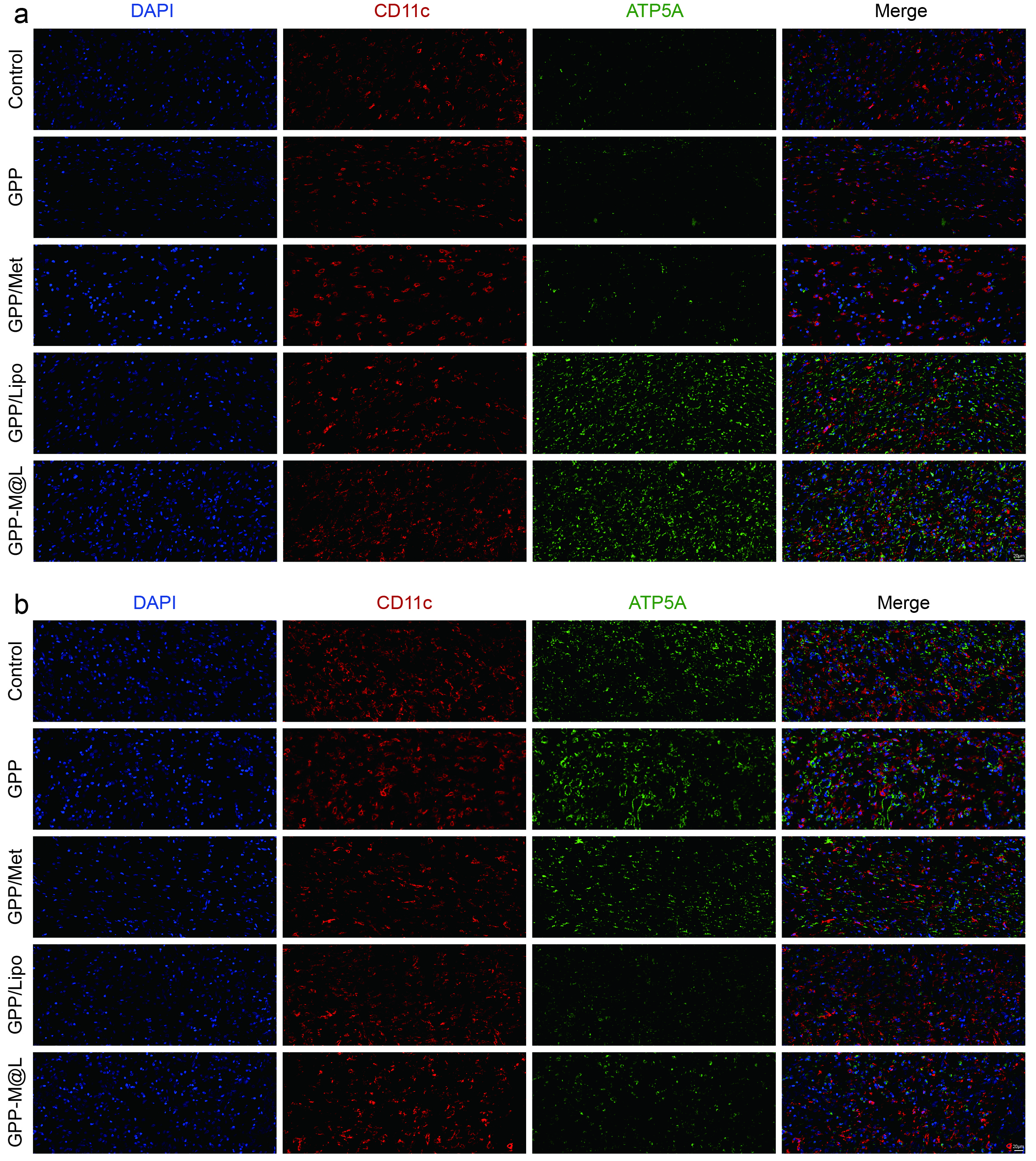


Figure S31. Histological analysis of diabetic wound tissues. Immunohistochemical staining was performed for CD11c (red), ATP5A (green), and cell nuclei (blue) on day 7 (a) and day 14 (b) (Scale bar: 20 μm).


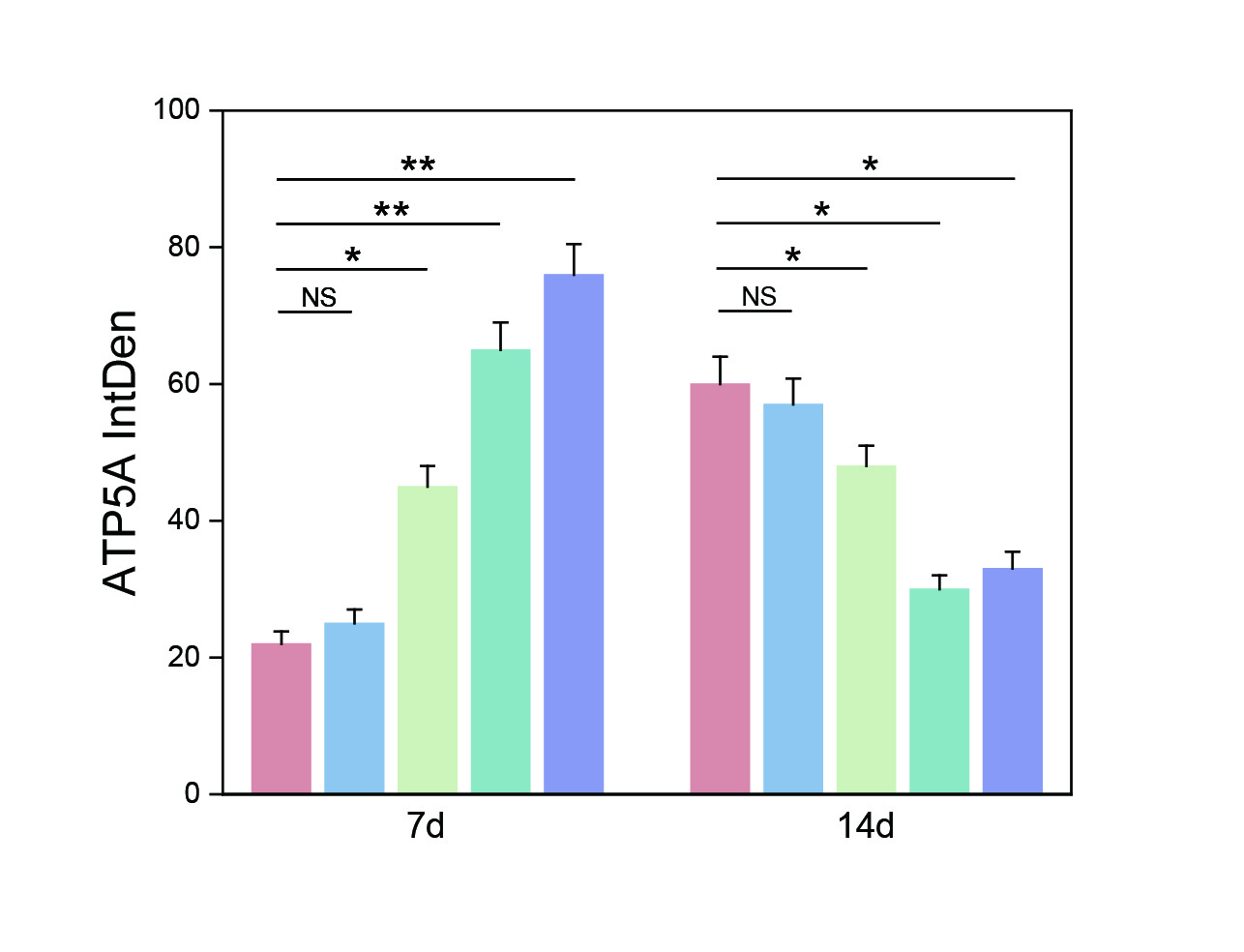


Figure S32. Quantitative analysis of the marker ATP5A expression in CD11c. Data are presented as mean ± SD; *P < 0.05, **P < 0.01; NS, not significant. The control group was under hyperglycemic conditions.


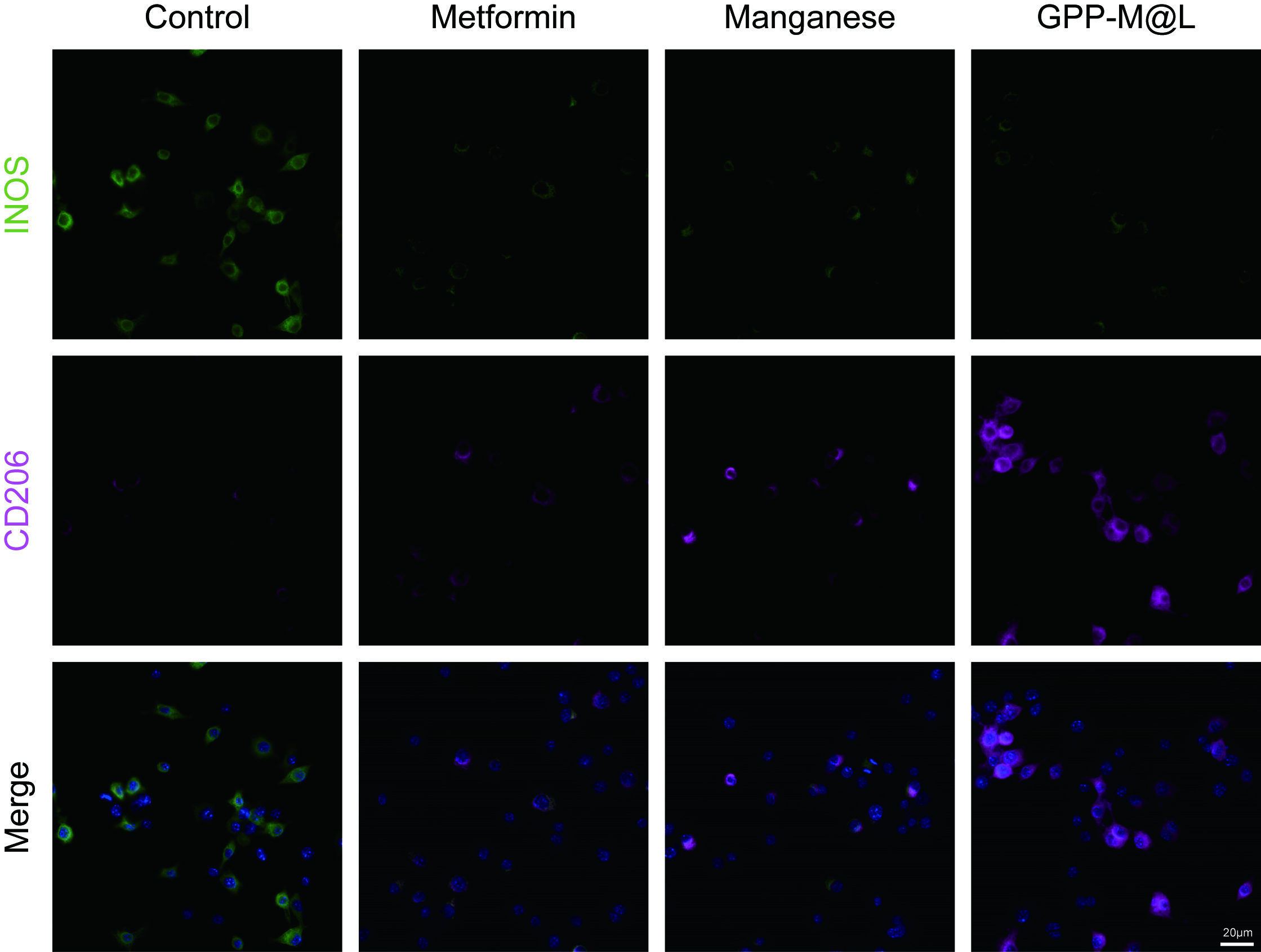


Figure S33: Immunofluorescence images of INOS and CD206 expression in different treatment groups: INOS (green), CD206 (red), DAPI (blue). Scale bar: 20 μm.


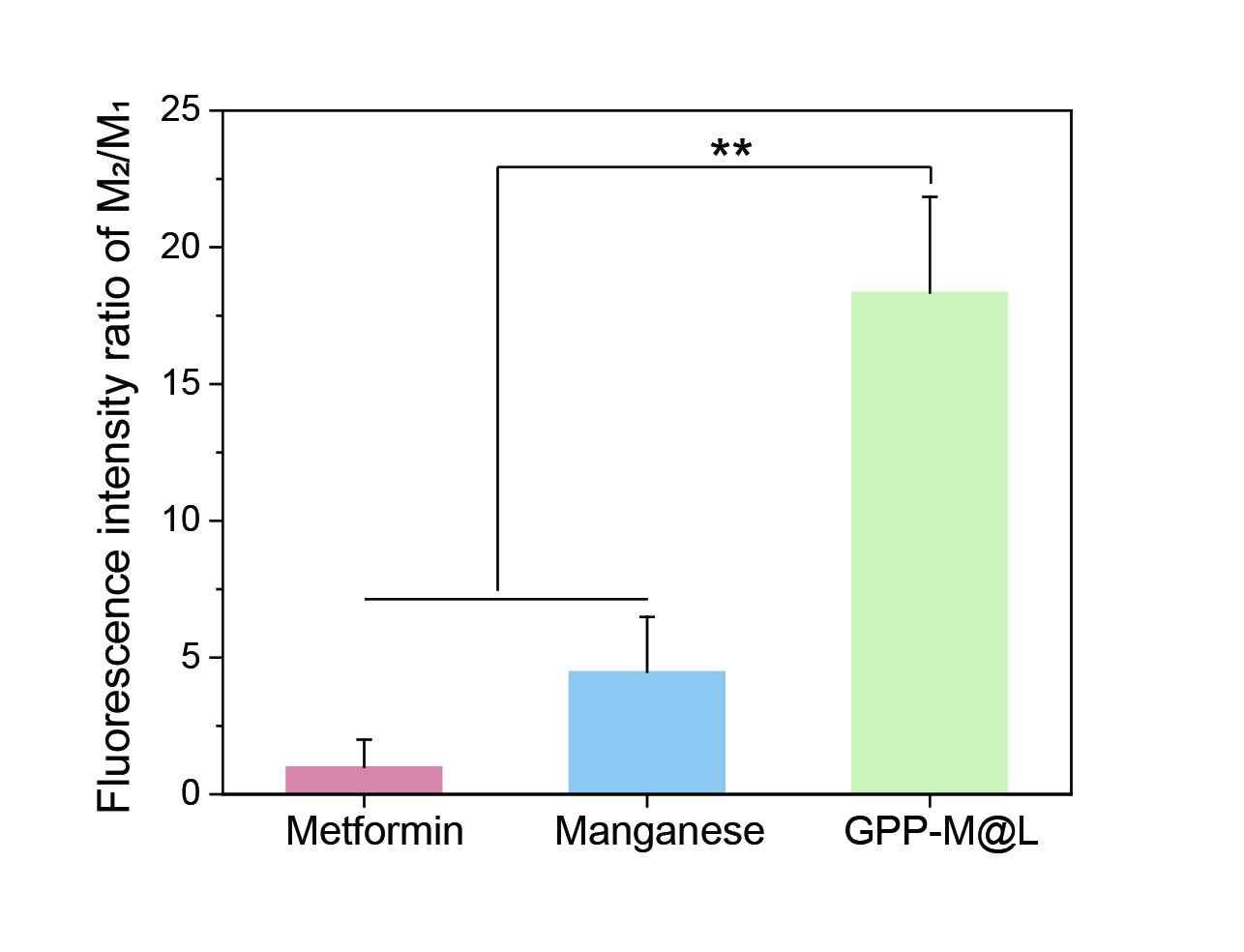


Figure S34: Quantitative analysis of M2/M1 expression. All values are presented as mean ± SD; *P ≤ 0.05; P ≥ 0.05 is considered non-significant (NS).
