# Supplementary figures and images for: Manganese-Based Polyoxometalate Nanozyme-Metformin Co-functionalized Hydrogel Promotes Diabetic Wound Regeneration by Enhancing Phagocyte Efferocytosis
Source: Research (Wash D C). 2025 Nov 27;8:0964. doi: 10.34133/research.0964 (PMC12697061; doi:10.34133/research.0964)

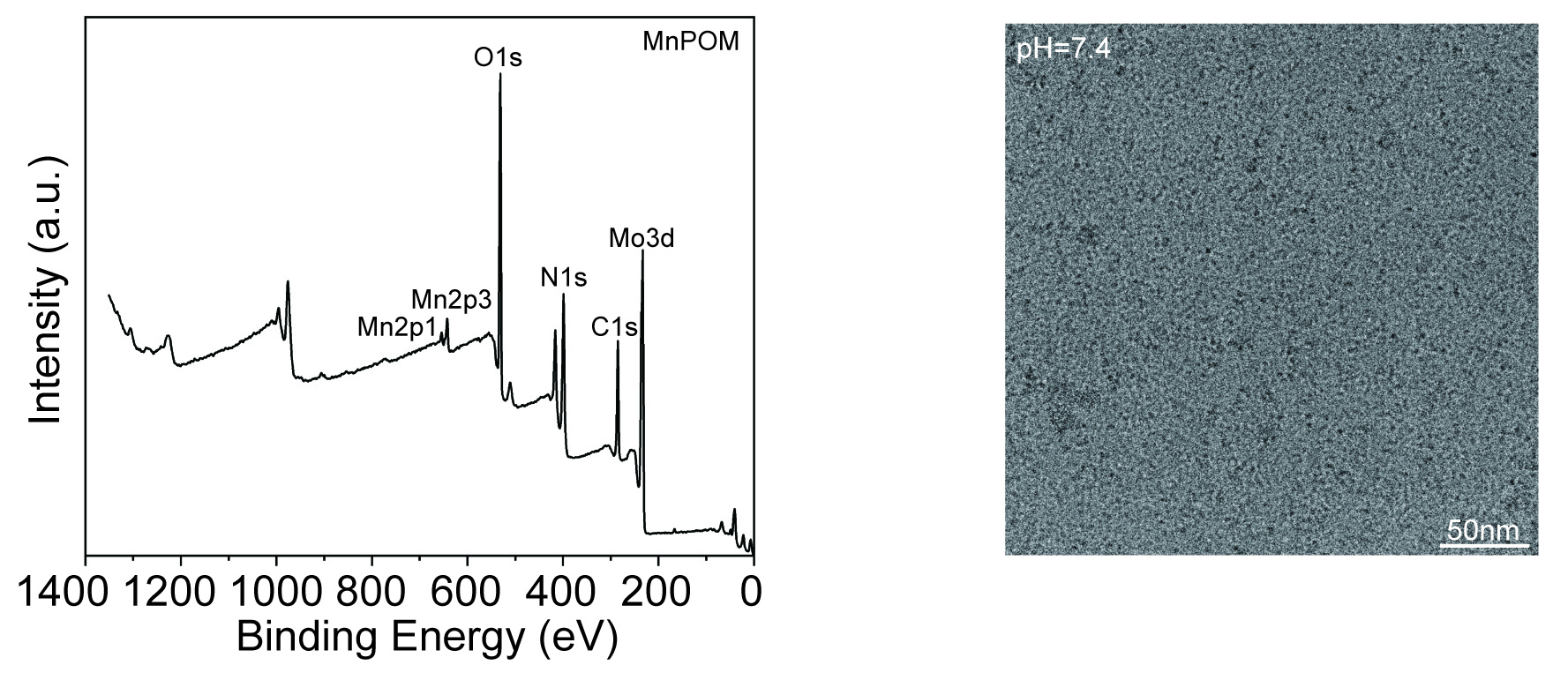

Supplement: Supplementary 1 — Figs. S1 to S34 [file research.0964.f1.zip › SI Figures/01. Figure S1.jpg]

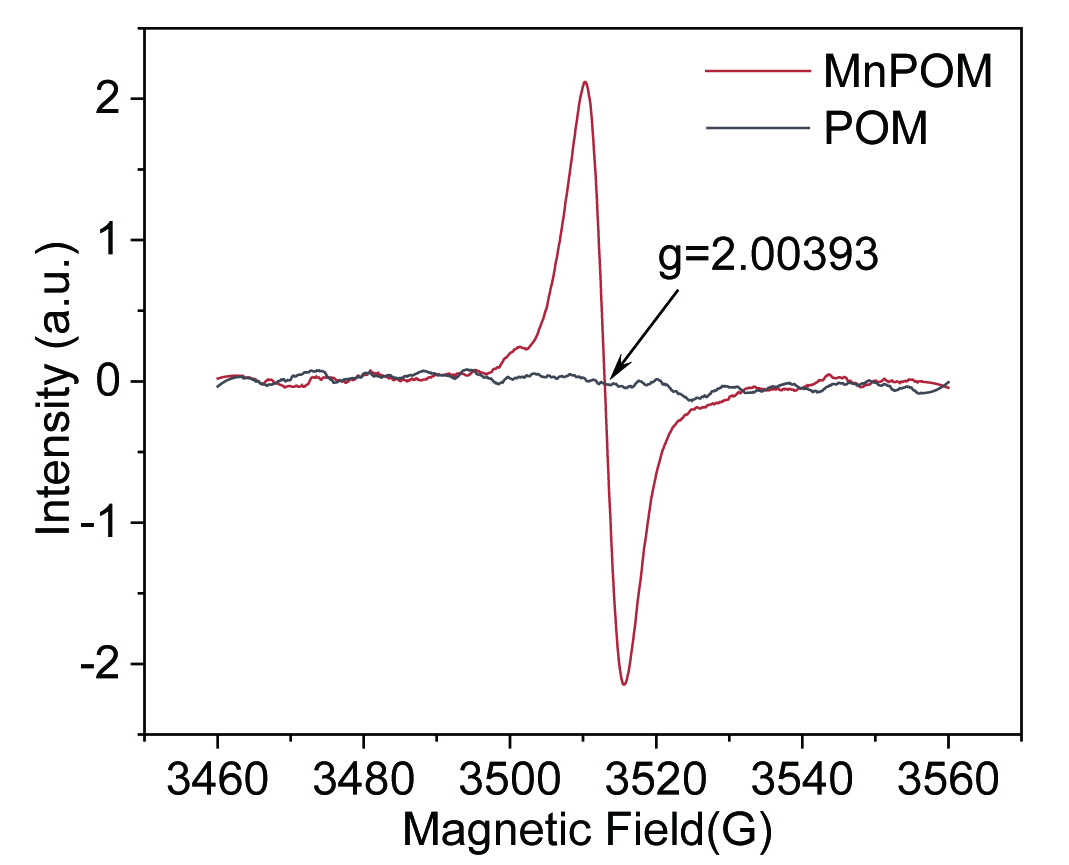

Supplement: Supplementary 1 — Figs. S1 to S34 [file research.0964.f1.zip › SI Figures/02. Figure S2.jpg]

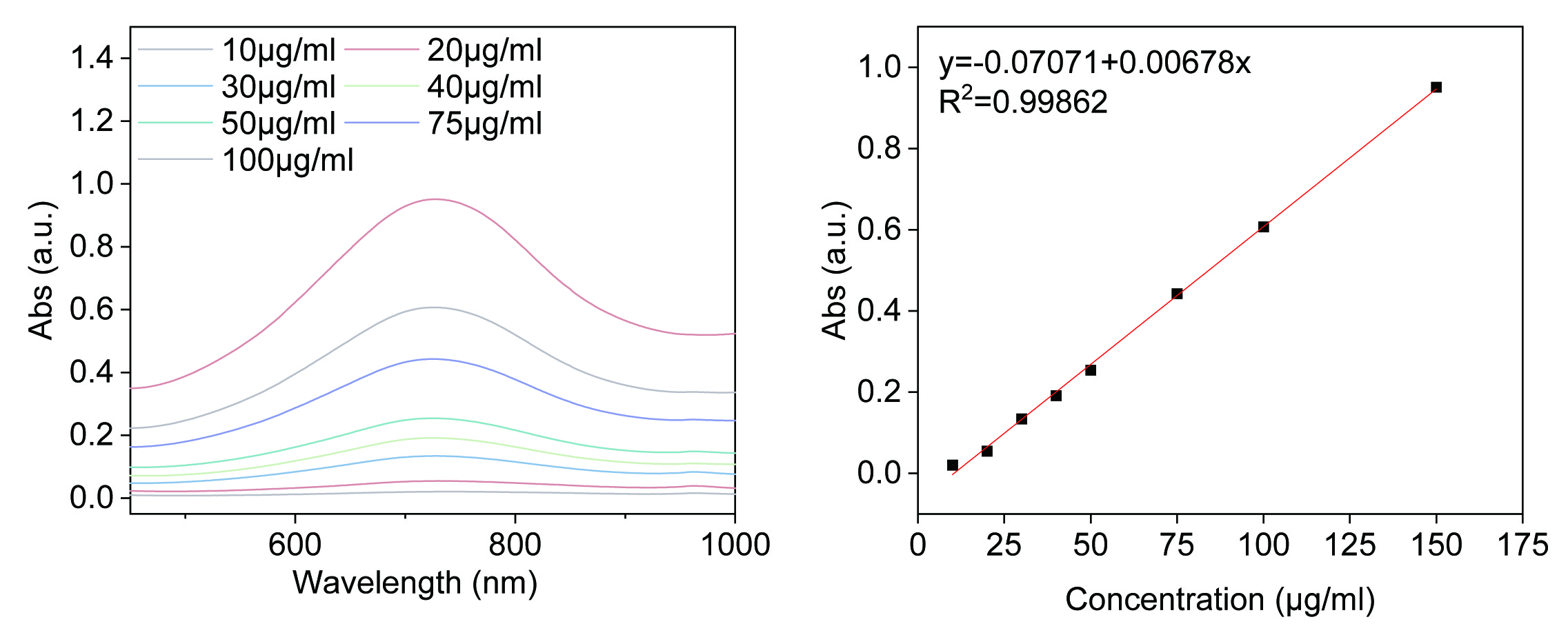

Supplement: Supplementary 1 — Figs. S1 to S34 [file research.0964.f1.zip › SI Figures/03. Figure S3.jpg]

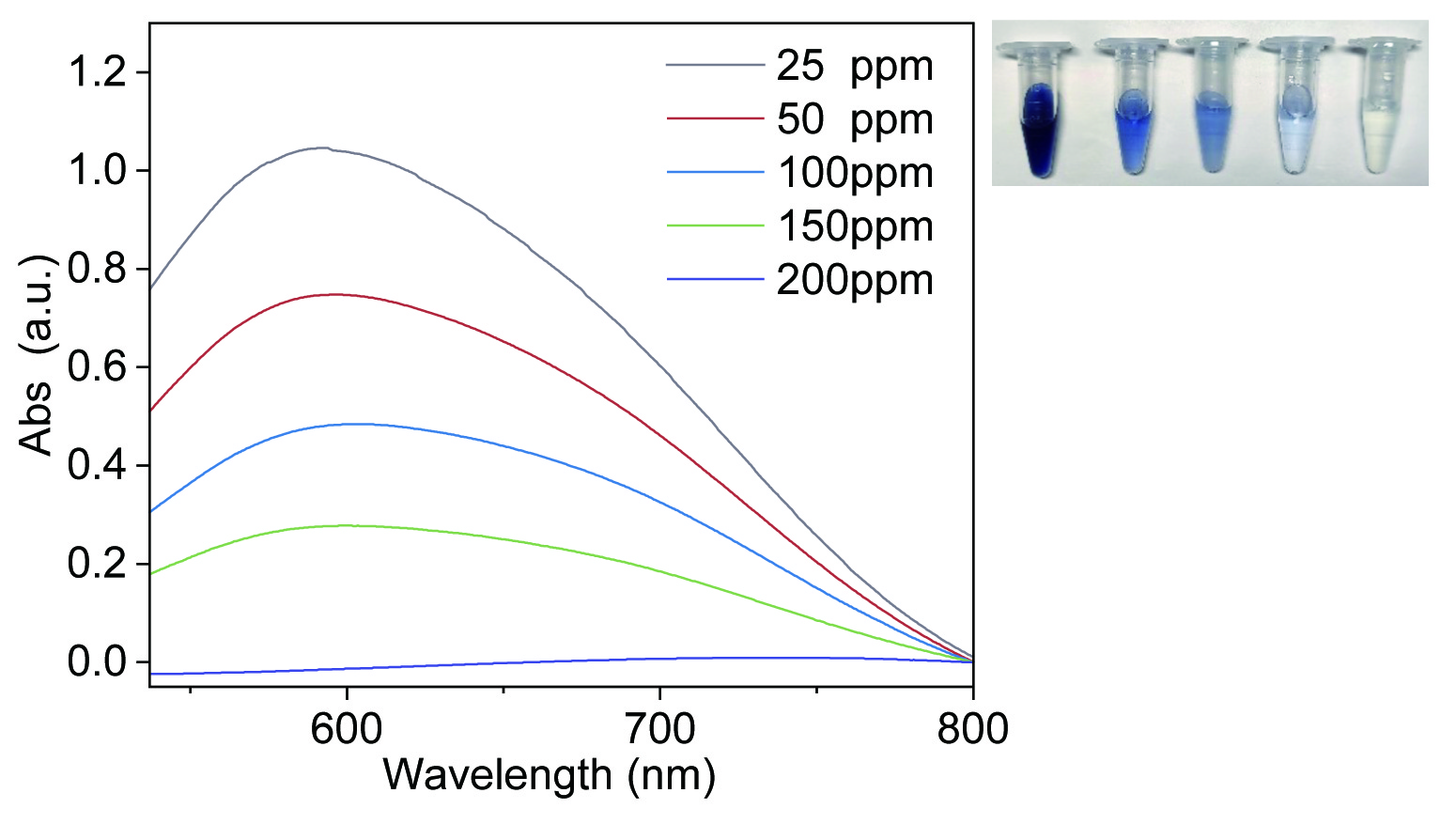

Supplement: Supplementary 1 — Figs. S1 to S34 [file research.0964.f1.zip › SI Figures/04. Figure S4.jpg]

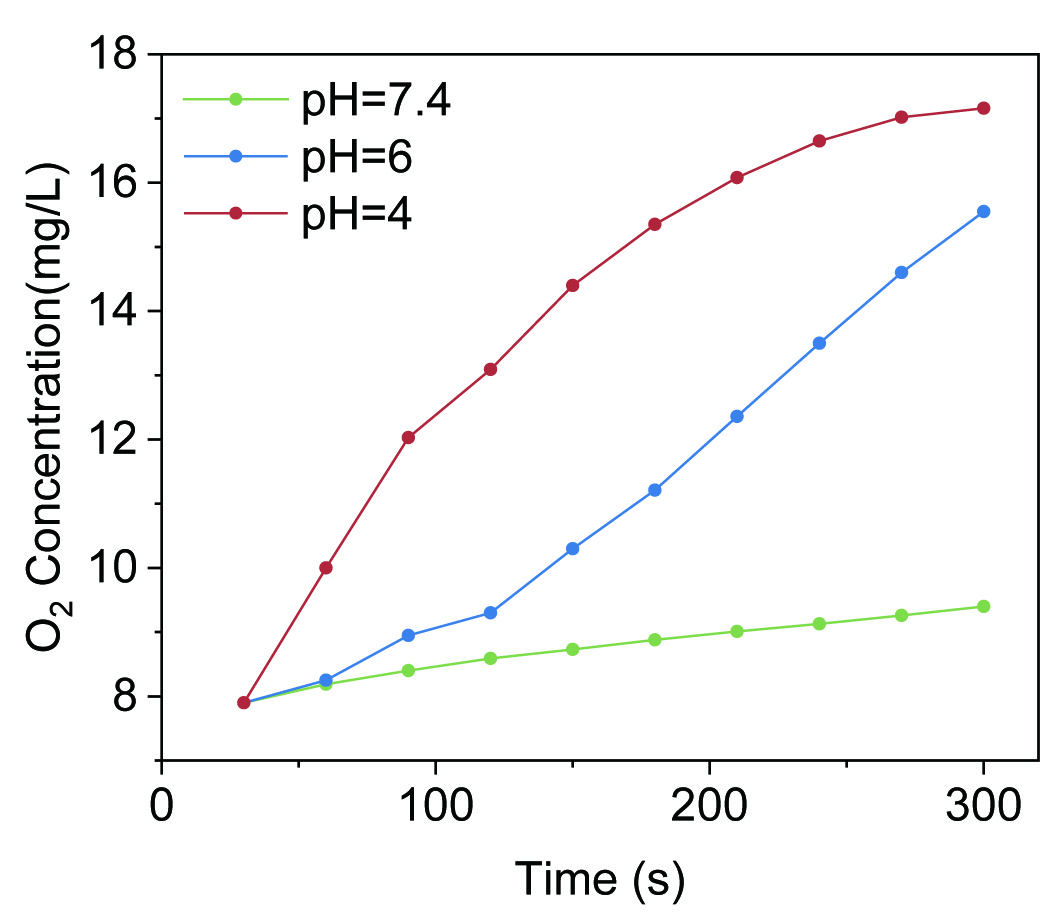

Supplement: Supplementary 1 — Figs. S1 to S34 [file research.0964.f1.zip › SI Figures/05. Figure S5.jpg]

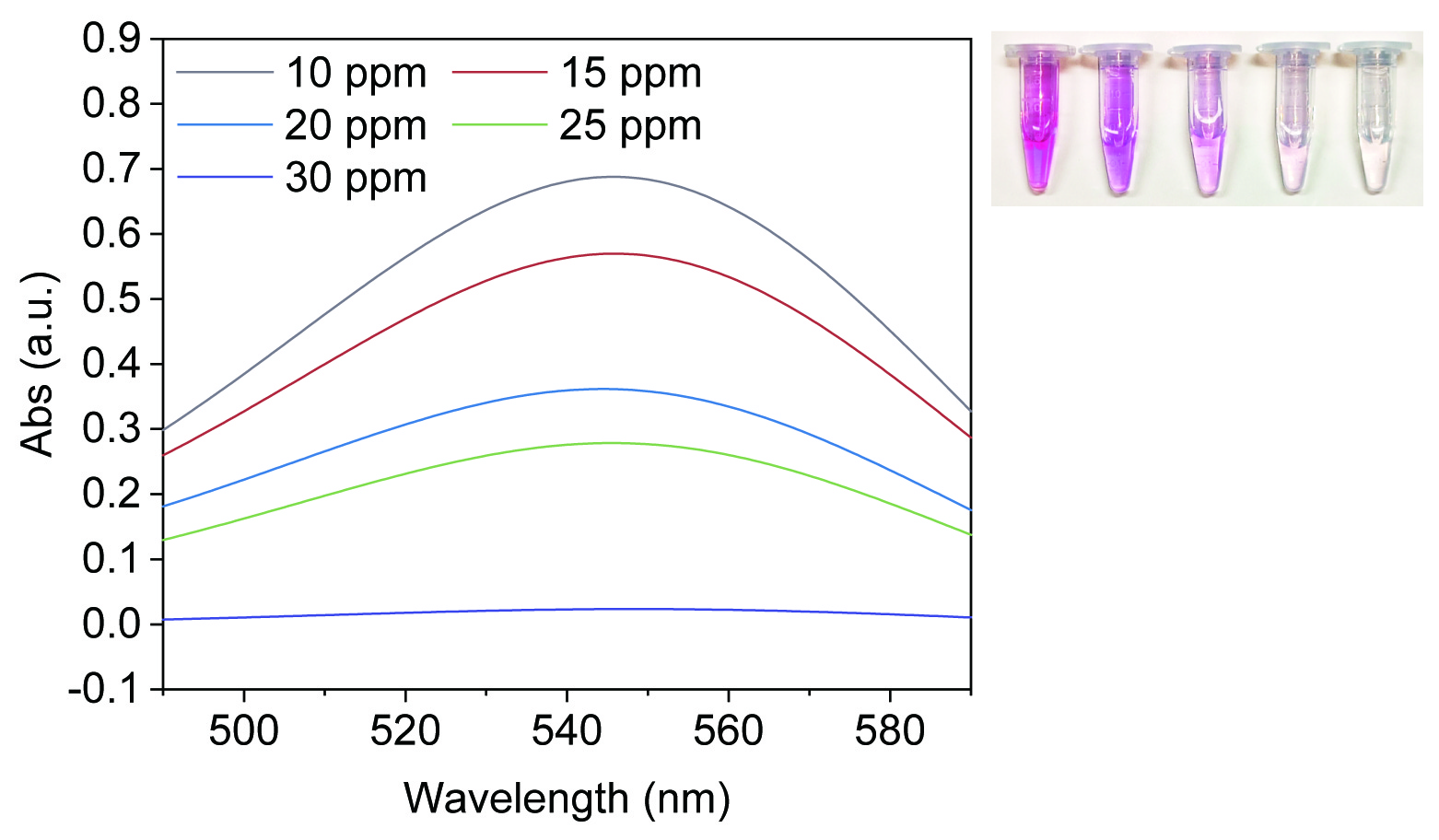

Supplement: Supplementary 1 — Figs. S1 to S34 [file research.0964.f1.zip › SI Figures/06. Figure S6.jpg]

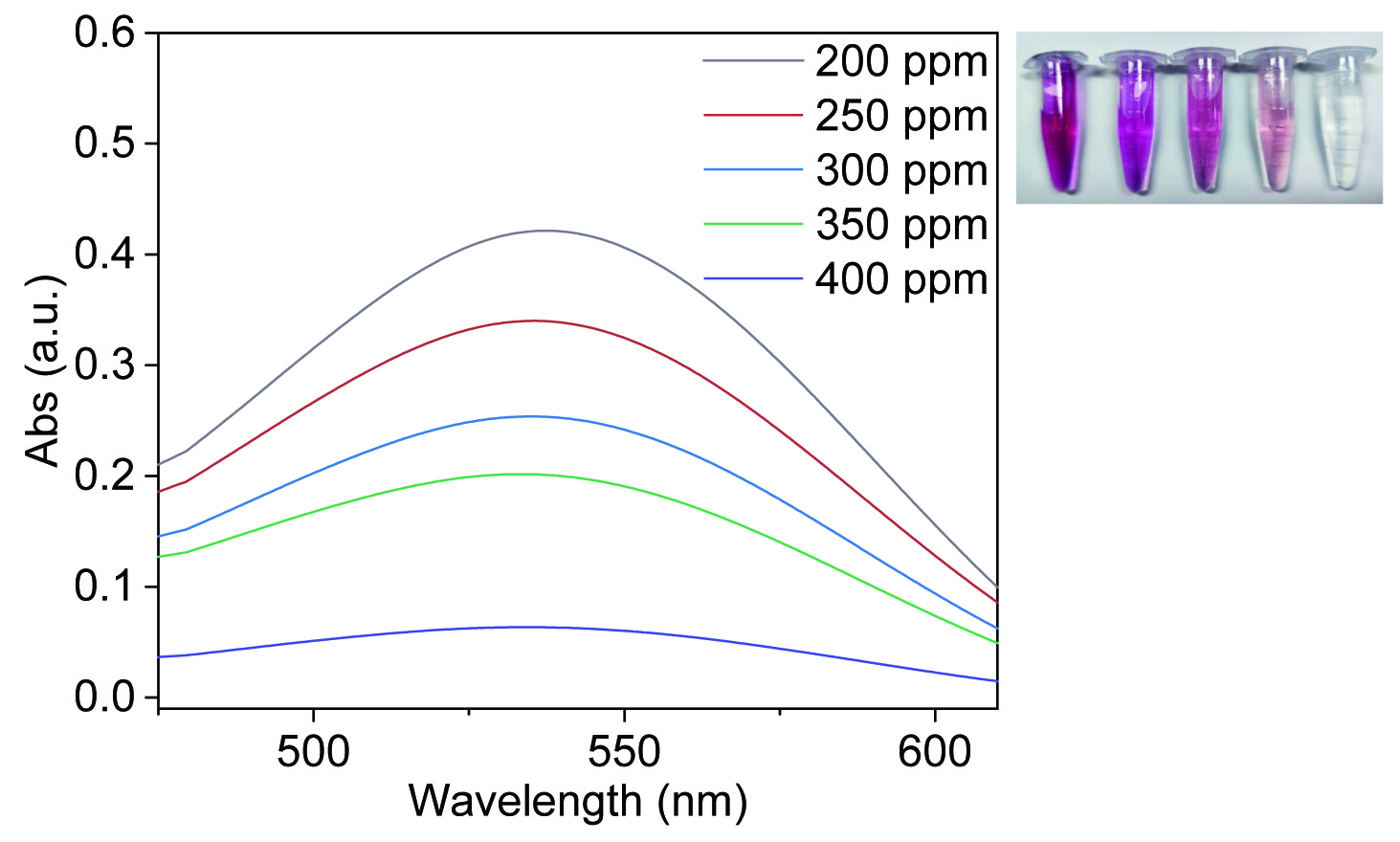

Supplement: Supplementary 1 — Figs. S1 to S34 [file research.0964.f1.zip › SI Figures/07. Figure S7.jpg]

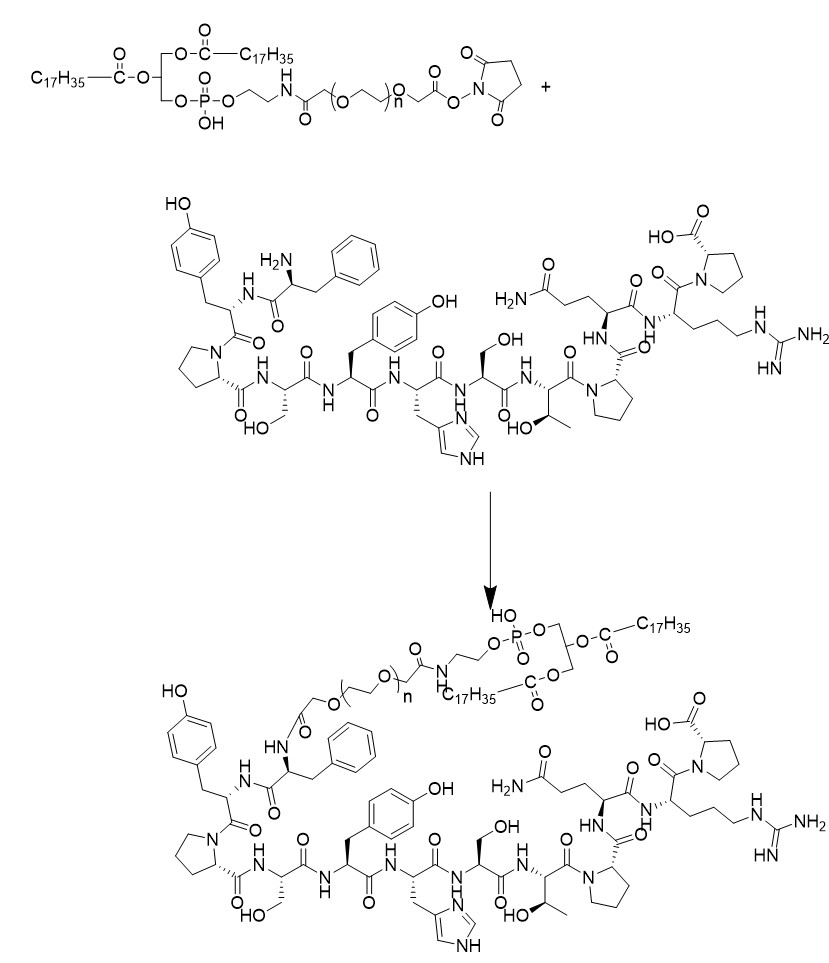

Supplement: Supplementary 1 — Figs. S1 to S34 [file research.0964.f1.zip › SI Figures/08. Figure S8.jpg]

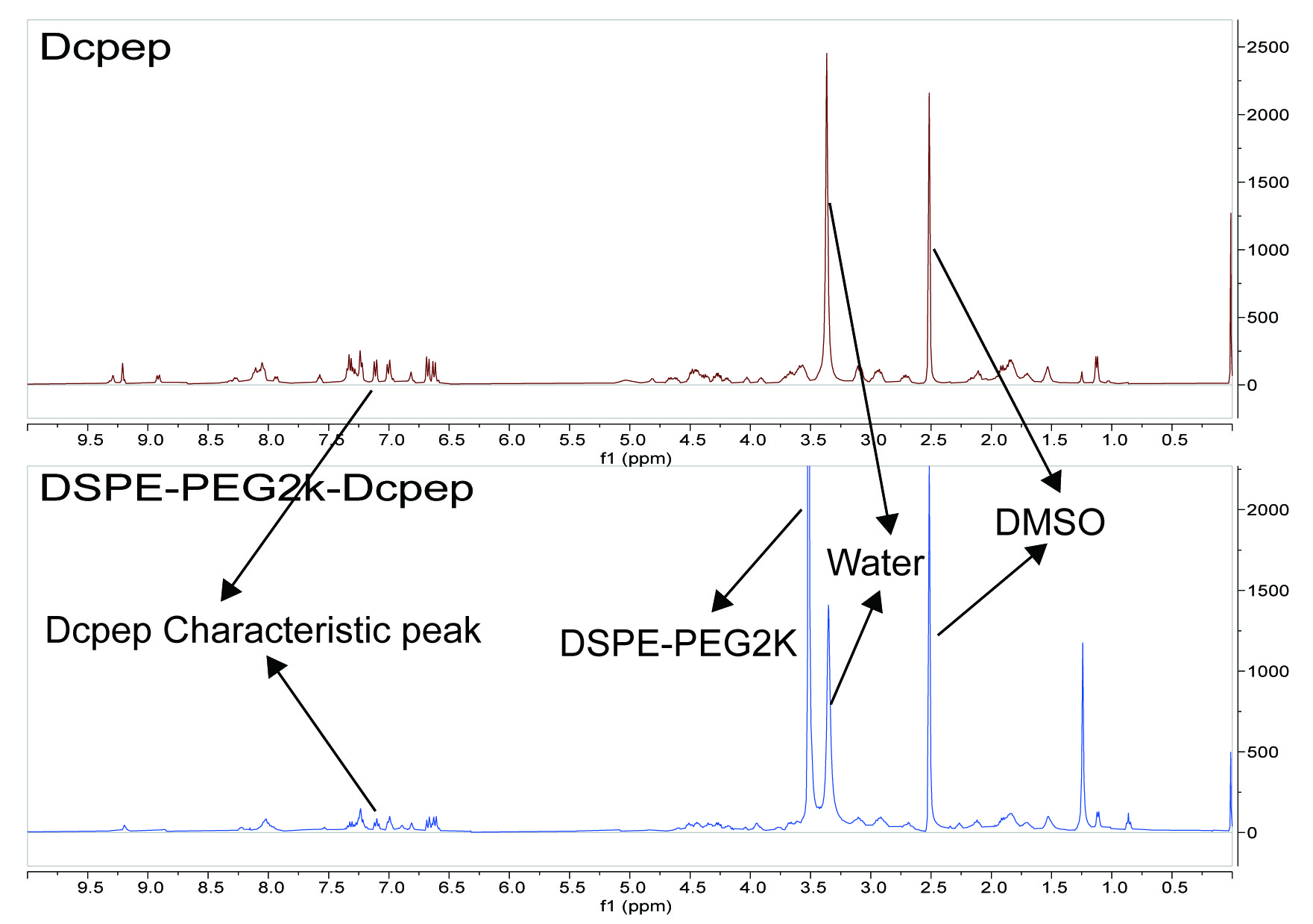

Supplement: Supplementary 1 — Figs. S1 to S34 [file research.0964.f1.zip › SI Figures/09. Figure S9.jpg]

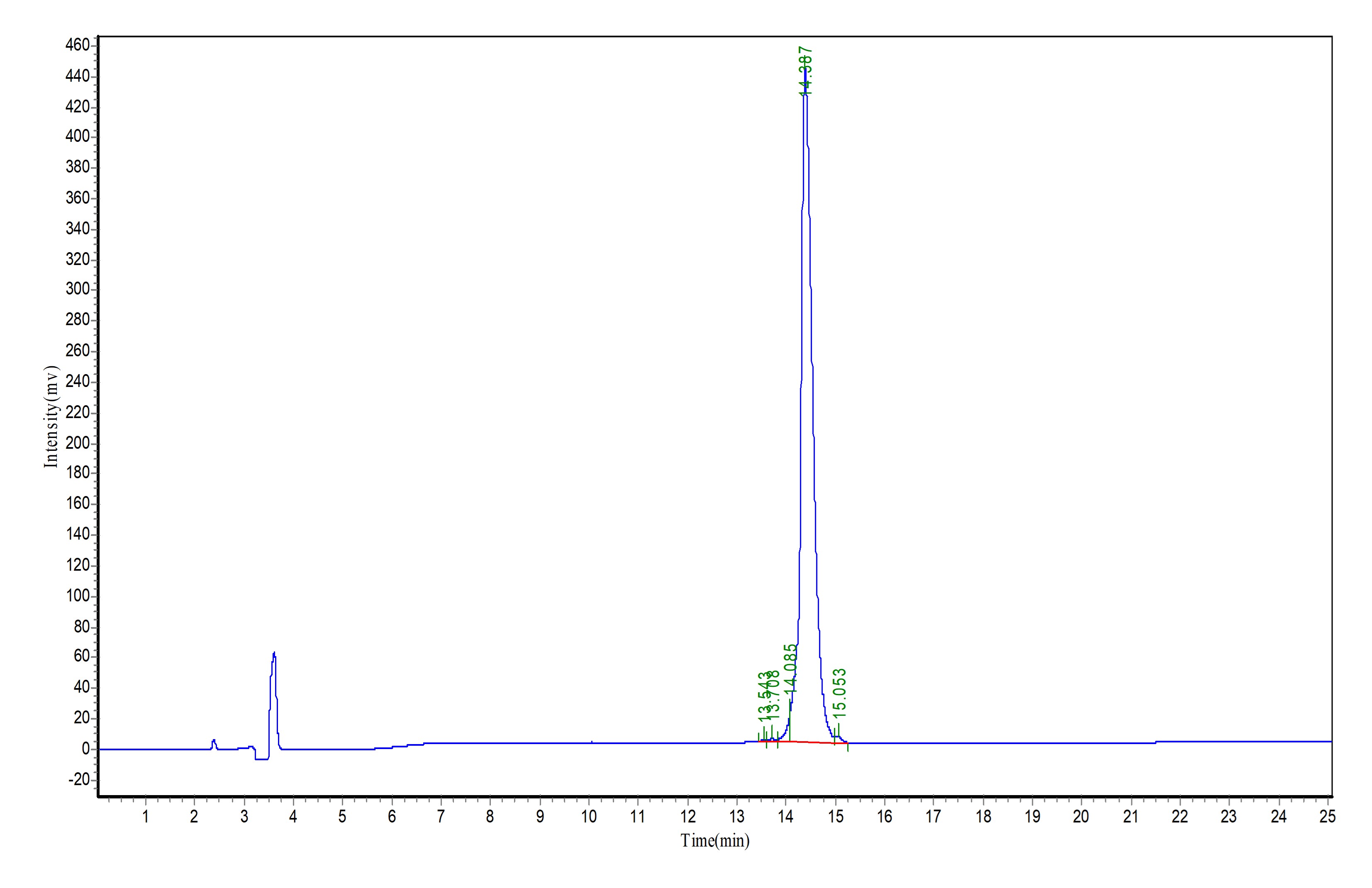

Supplement: Supplementary 1 — Figs. S1 to S34 [file research.0964.f1.zip › SI Figures/10. Figure S10.jpg]

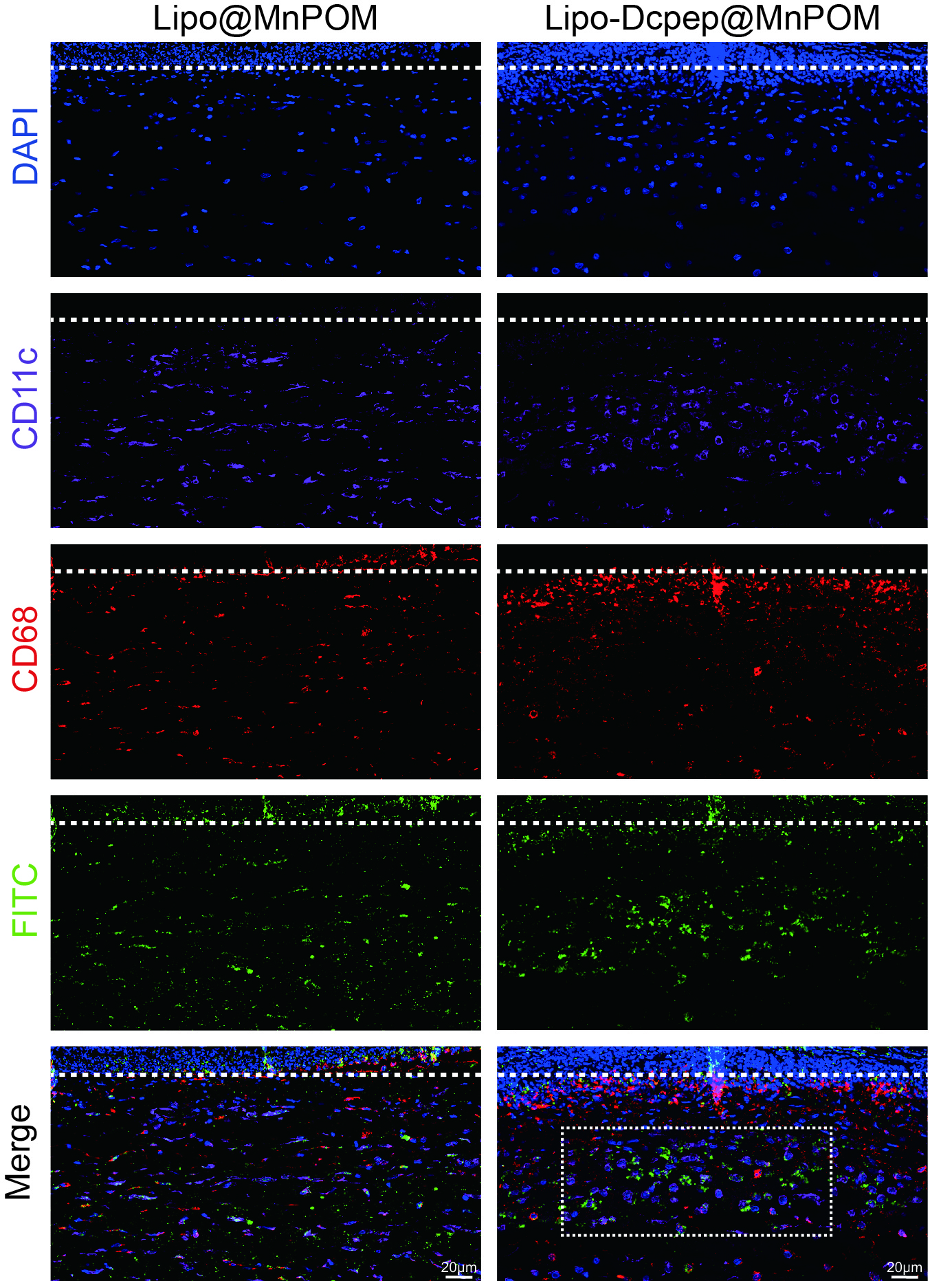

Supplement: Supplementary 1 — Figs. S1 to S34 [file research.0964.f1.zip › SI Figures/11. Figure S11.jpg]

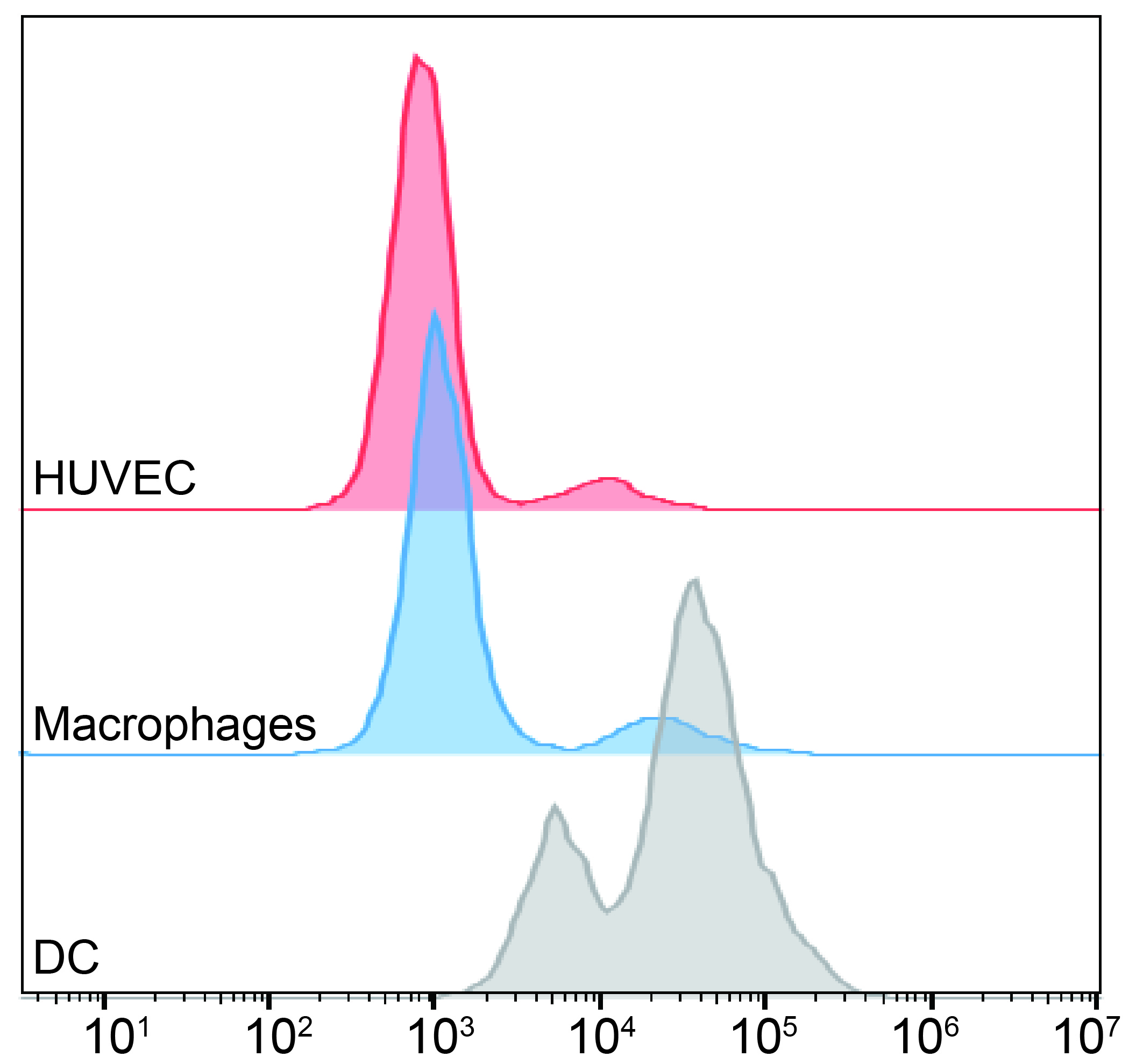

Supplement: Supplementary 1 — Figs. S1 to S34 [file research.0964.f1.zip › SI Figures/12. Figure S12.jpg]

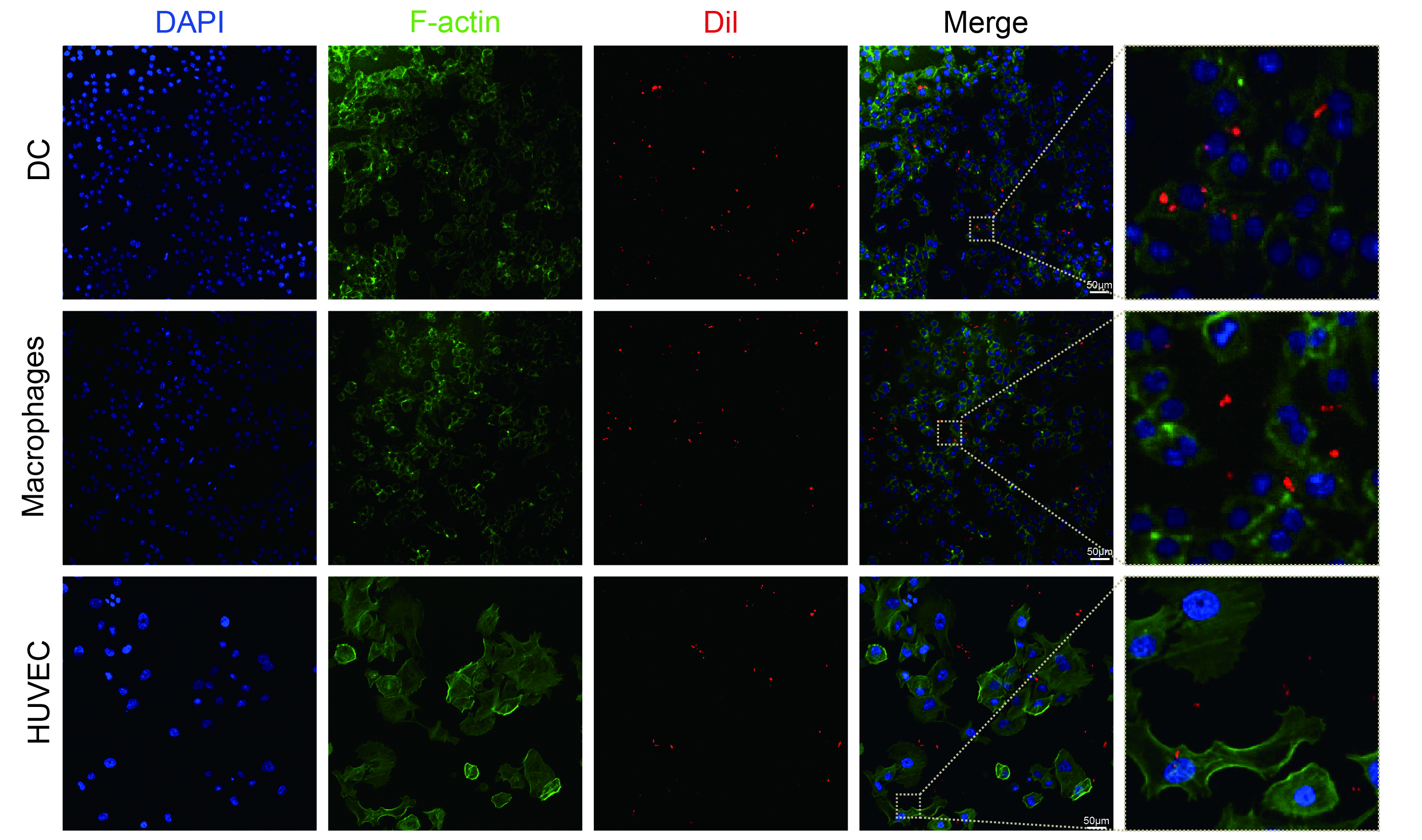

Supplement: Supplementary 1 — Figs. S1 to S34 [file research.0964.f1.zip › SI Figures/13. Figure S13.jpg]

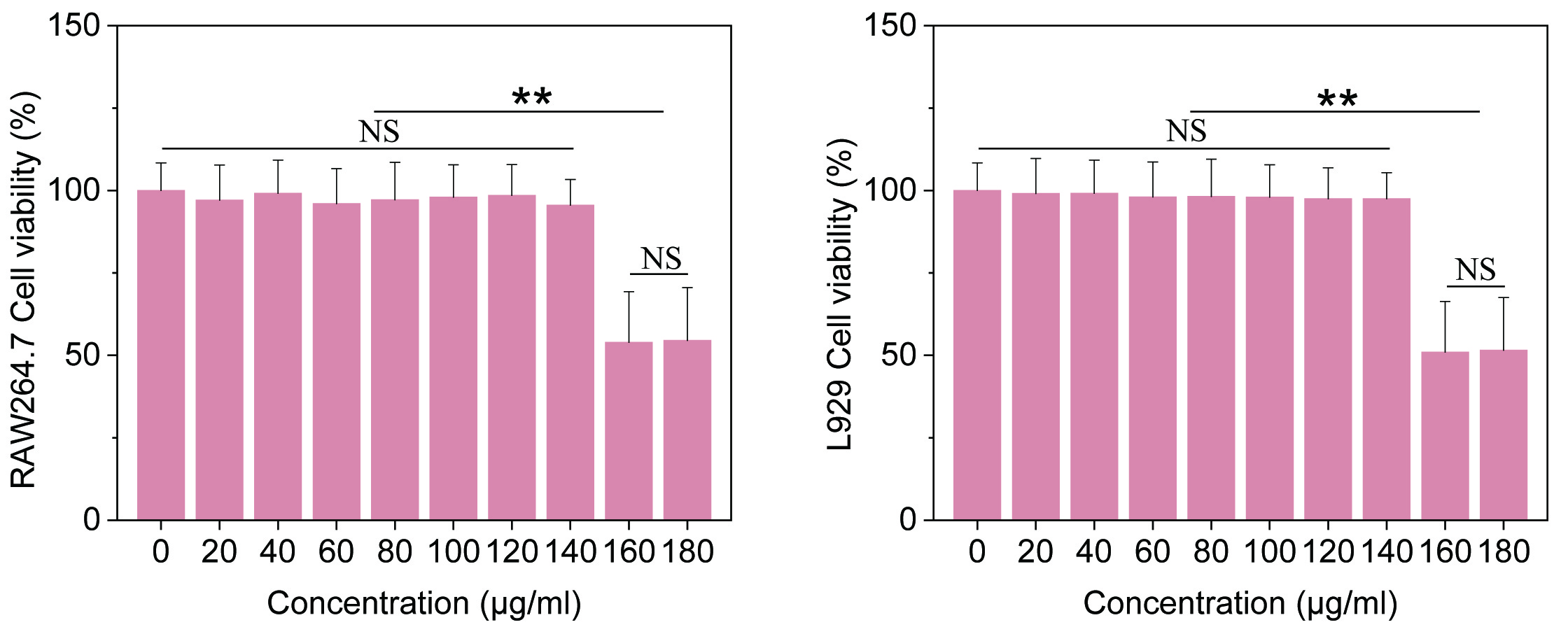

Supplement: Supplementary 1 — Figs. S1 to S34 [file research.0964.f1.zip › SI Figures/14. Figure S14.jpg]

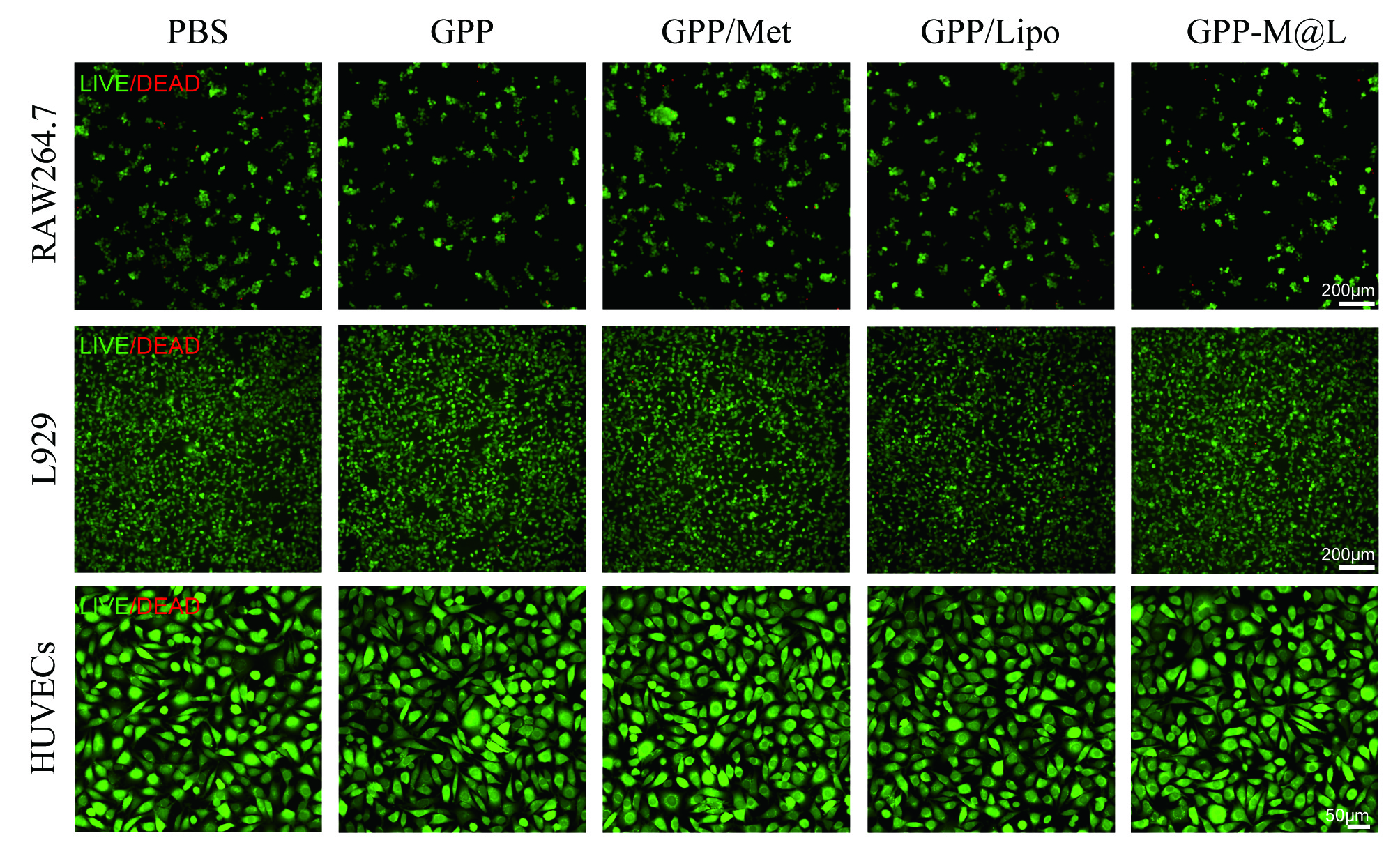

Supplement: Supplementary 1 — Figs. S1 to S34 [file research.0964.f1.zip › SI Figures/15. Figure S15.jpg]

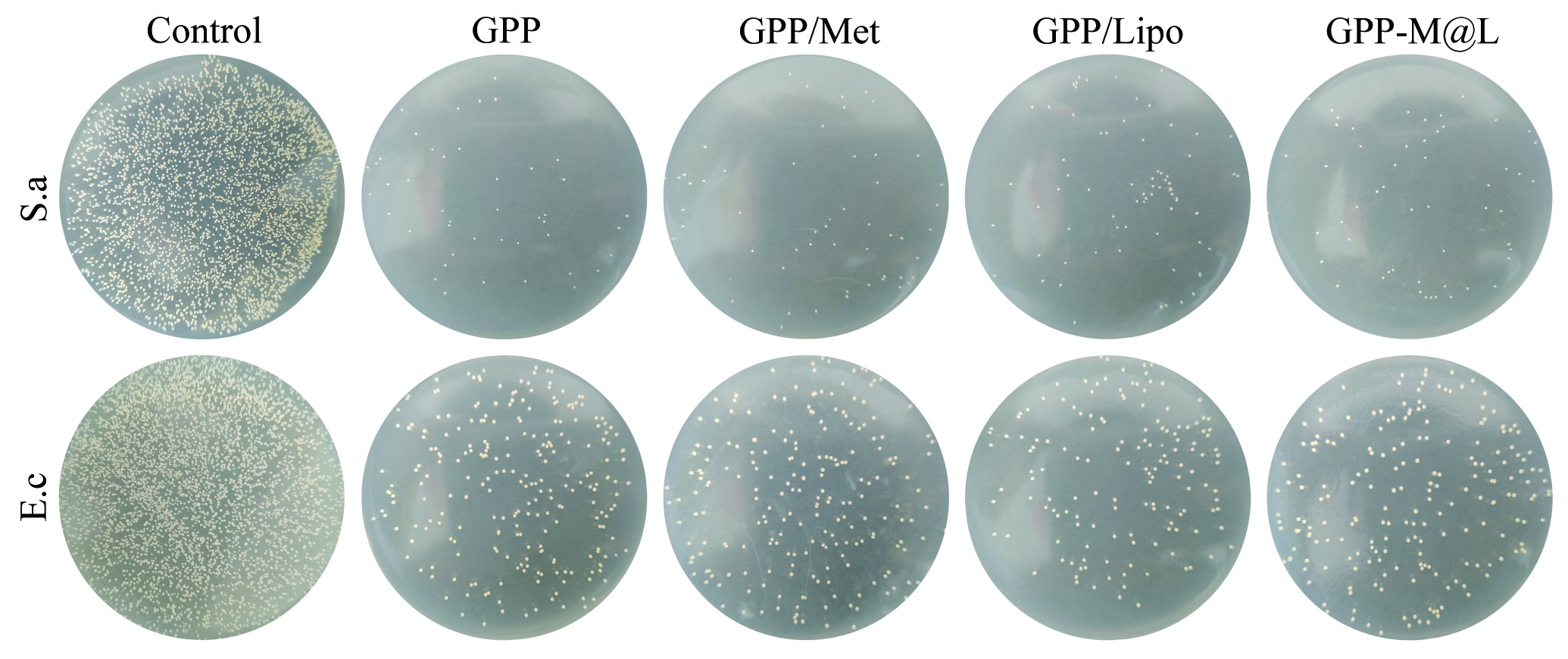

Supplement: Supplementary 1 — Figs. S1 to S34 [file research.0964.f1.zip › SI Figures/16. Figure S16.jpg]

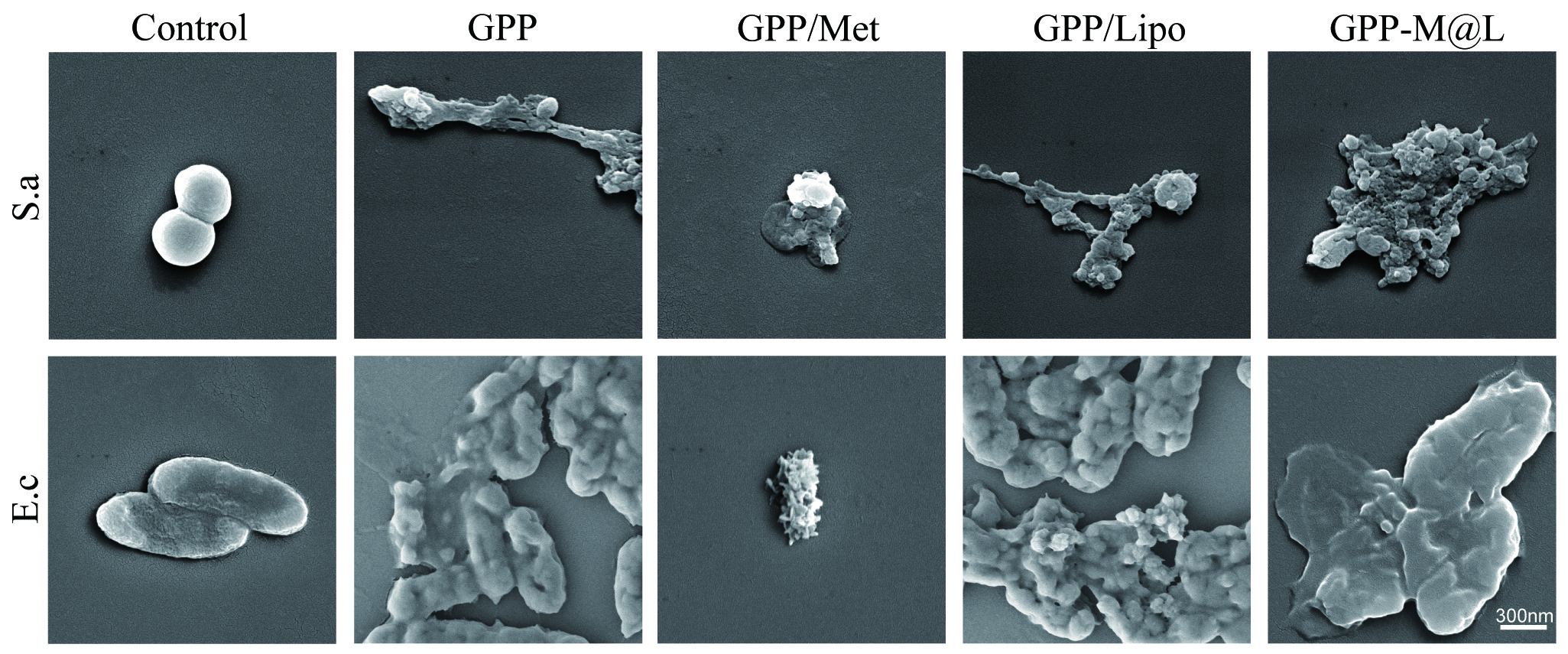

Supplement: Supplementary 1 — Figs. S1 to S34 [file research.0964.f1.zip › SI Figures/17. Figure S17.jpg]

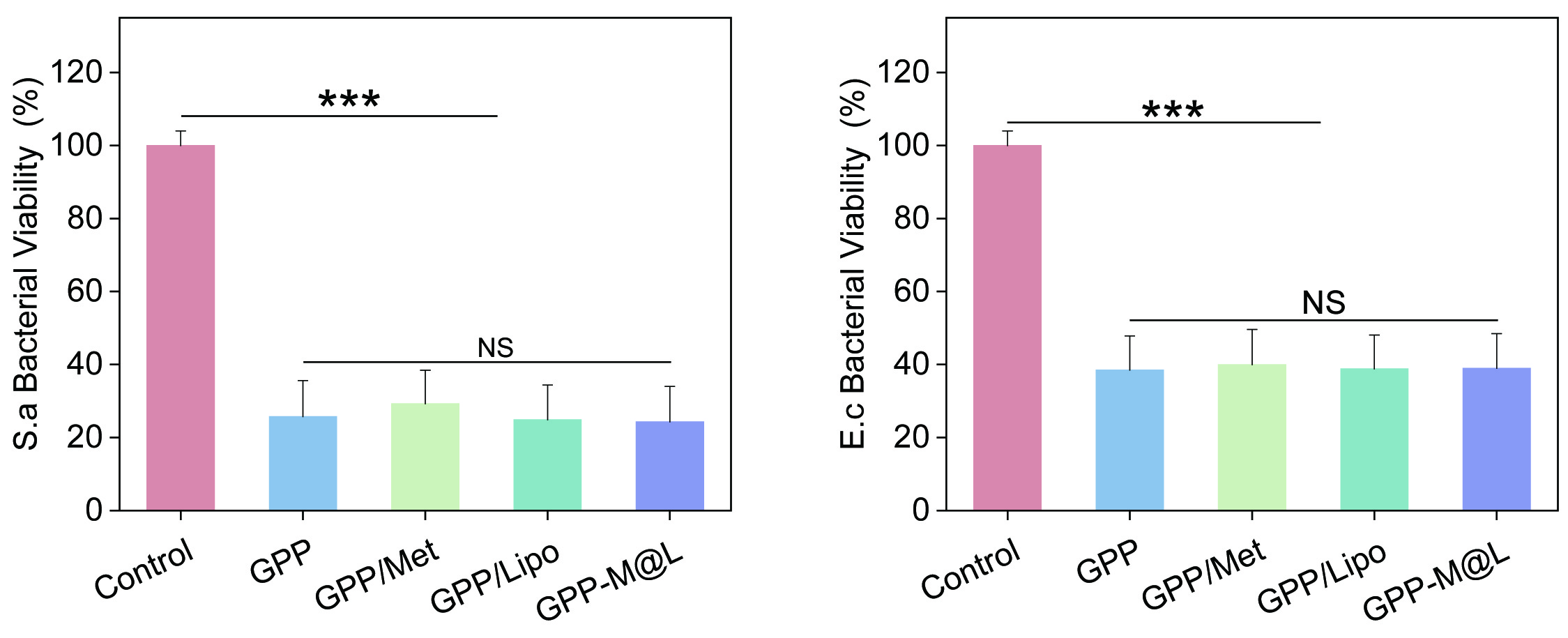

Supplement: Supplementary 1 — Figs. S1 to S34 [file research.0964.f1.zip › SI Figures/18. Figure S18.jpg]

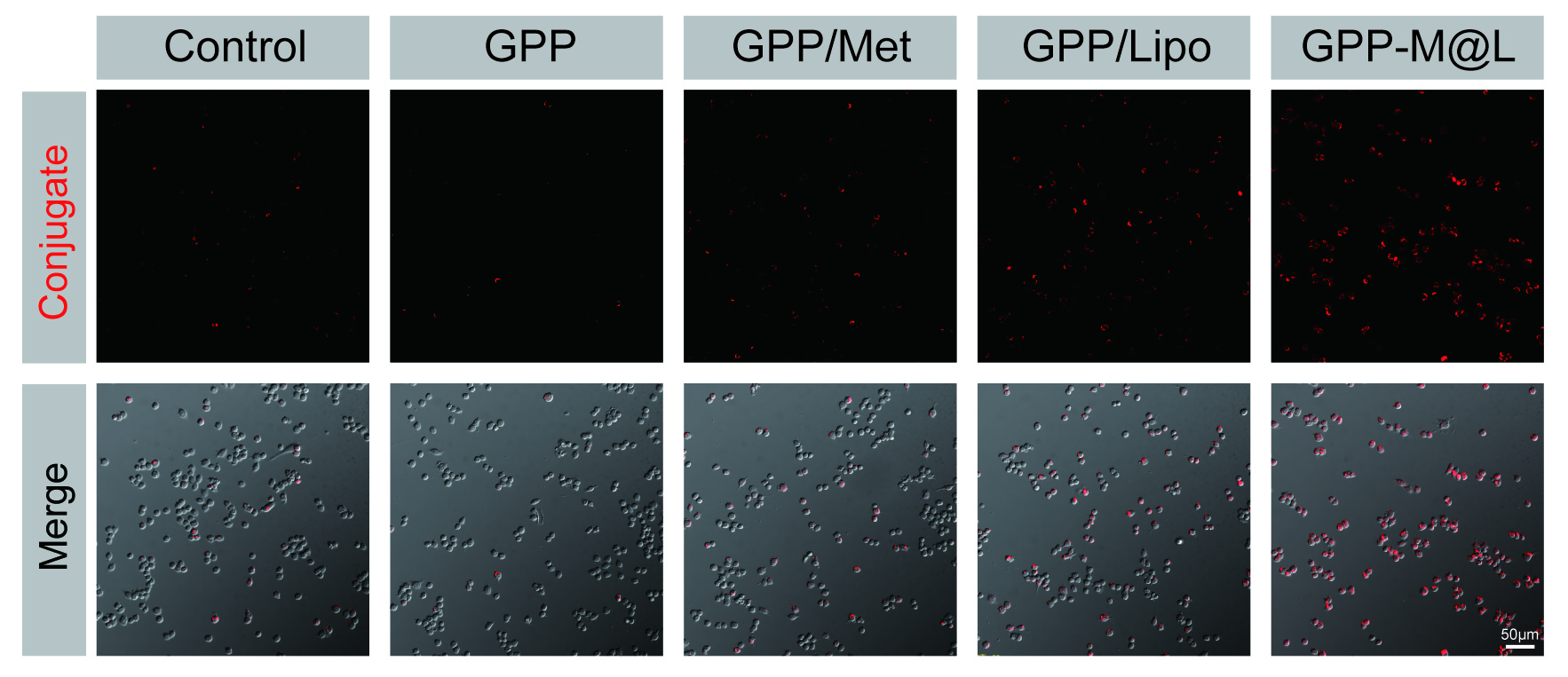

Supplement: Supplementary 1 — Figs. S1 to S34 [file research.0964.f1.zip › SI Figures/19. Figure S19.jpg]

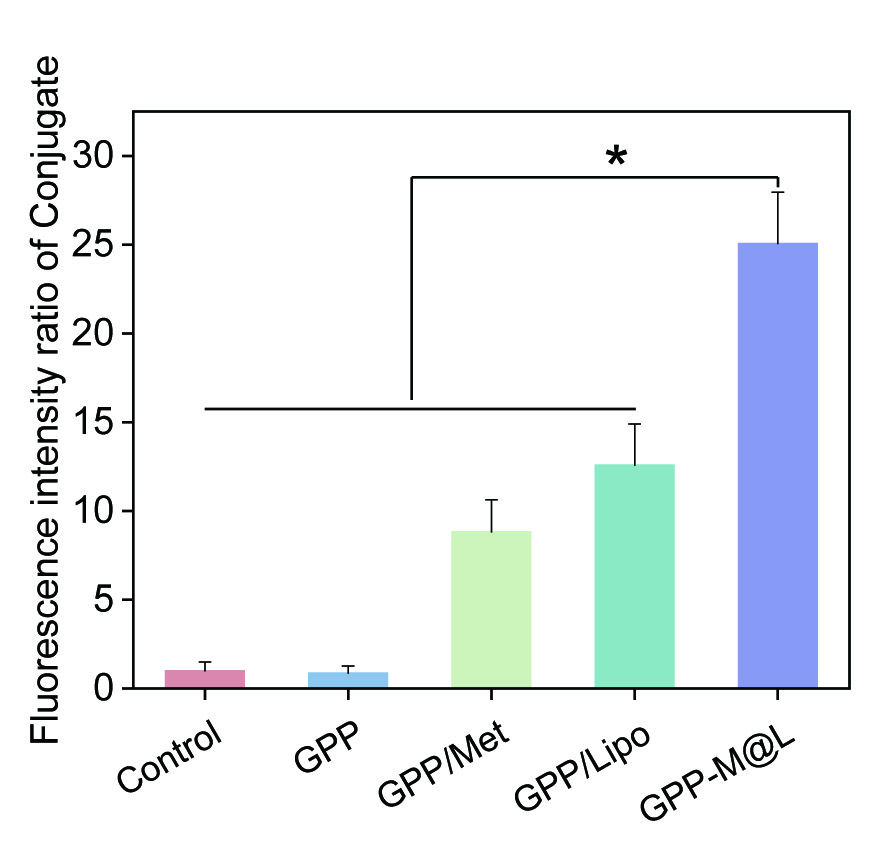

Supplement: Supplementary 1 — Figs. S1 to S34 [file research.0964.f1.zip › SI Figures/20. Figure S20.jpg]

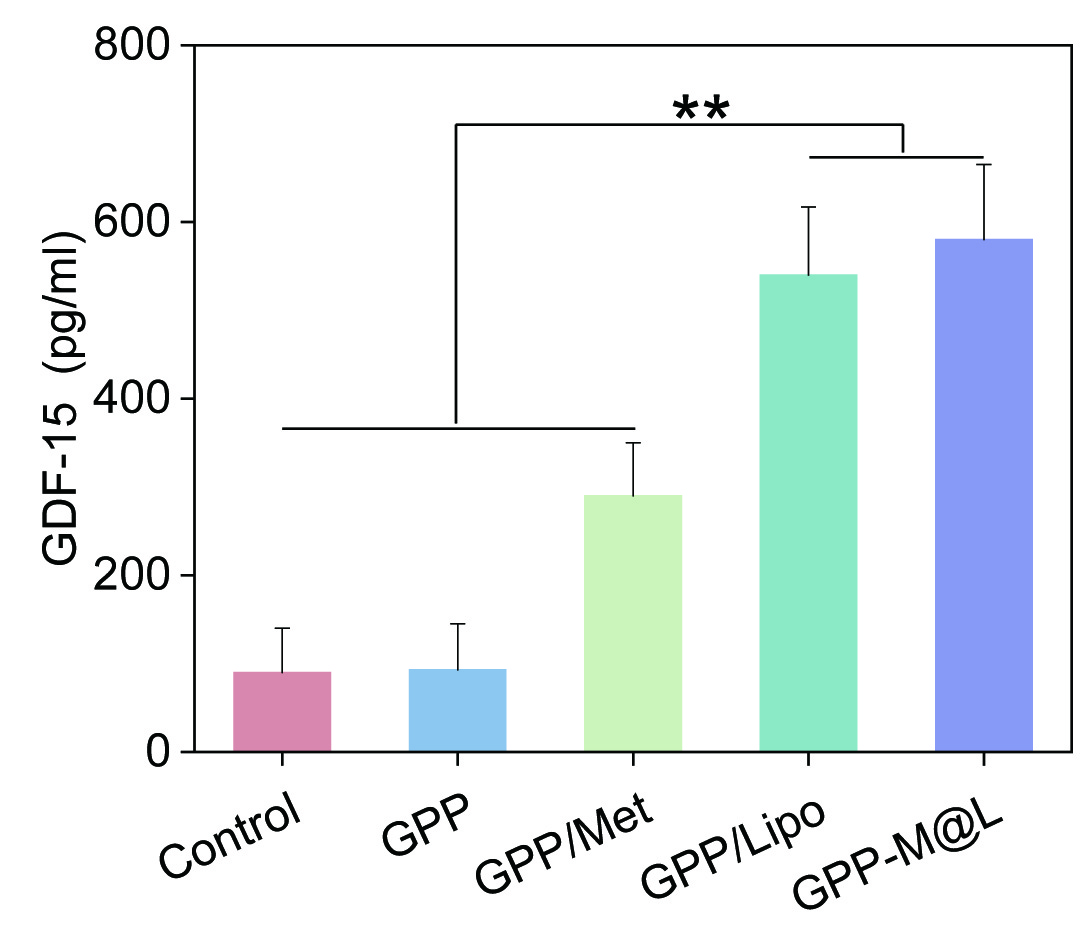

Supplement: Supplementary 1 — Figs. S1 to S34 [file research.0964.f1.zip › SI Figures/21. Figure S21.jpg]

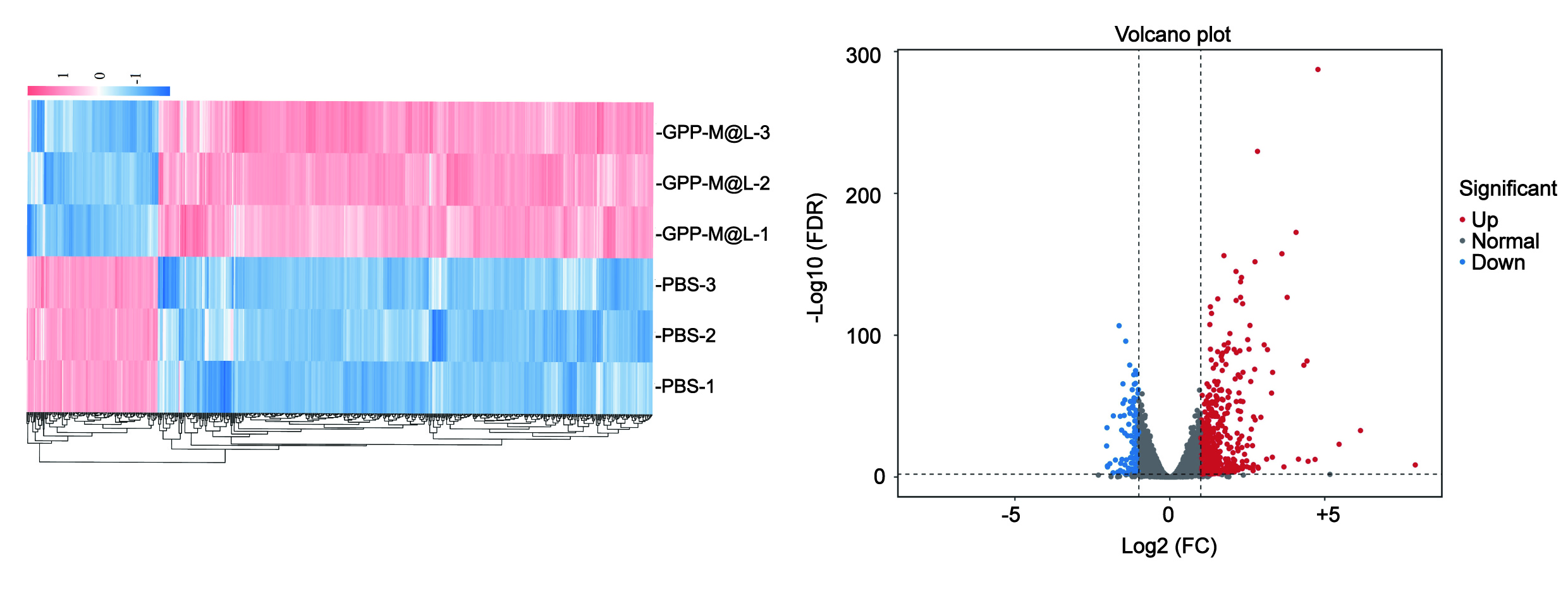

Supplement: Supplementary 1 — Figs. S1 to S34 [file research.0964.f1.zip › SI Figures/22. Figure S22.jpg]

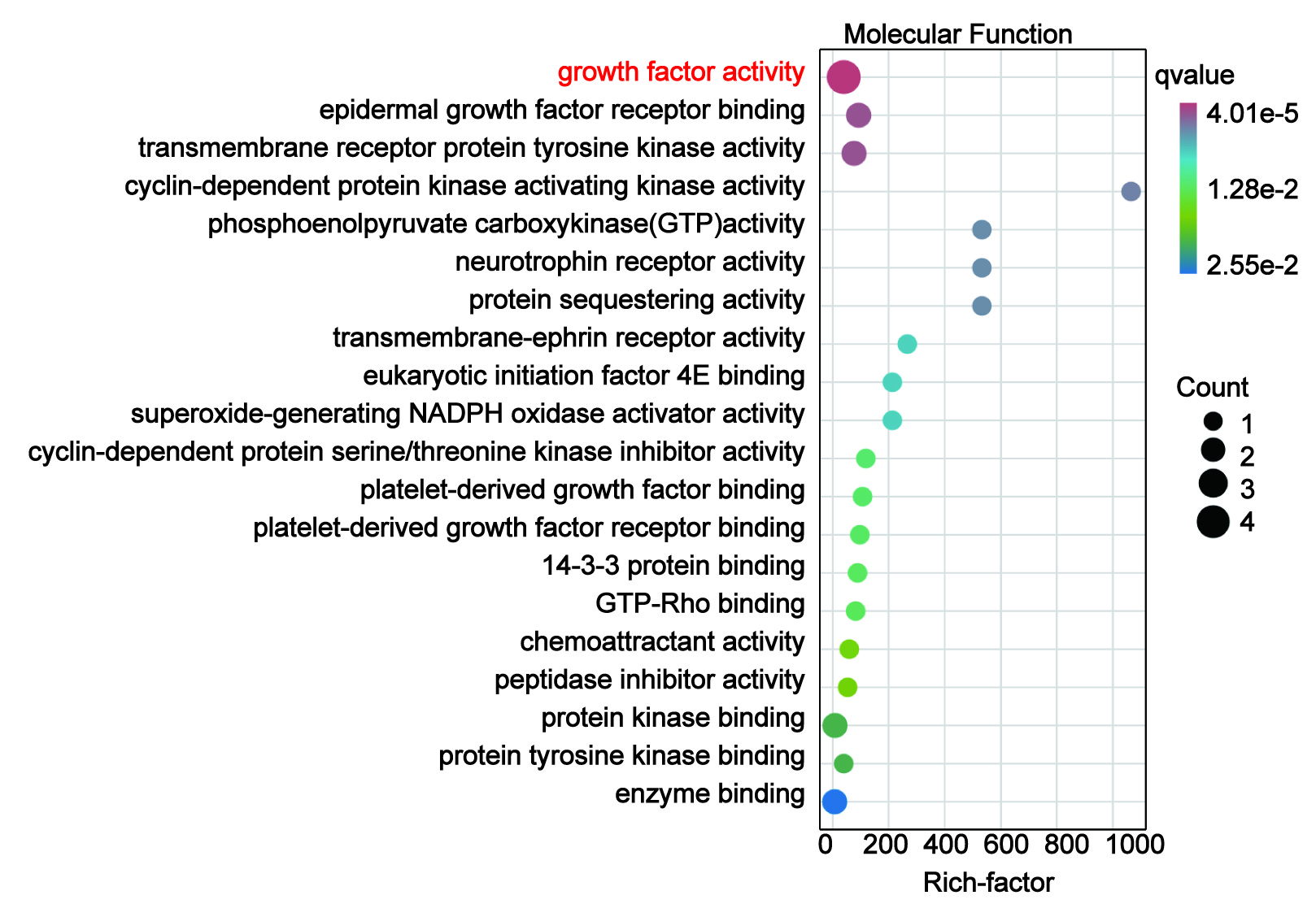

Supplement: Supplementary 1 — Figs. S1 to S34 [file research.0964.f1.zip › SI Figures/23. Figure S23.jpg]

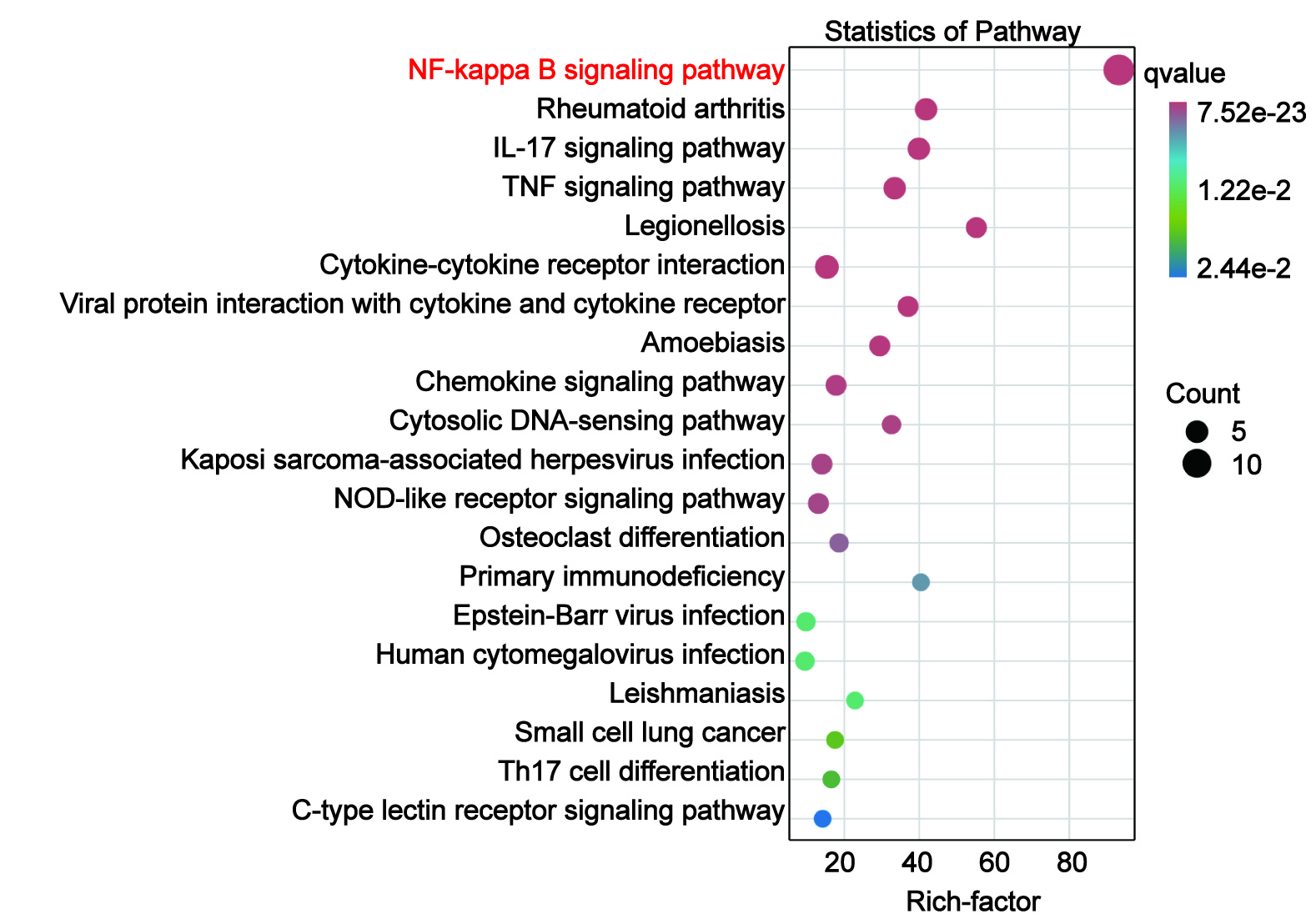

Supplement: Supplementary 1 — Figs. S1 to S34 [file research.0964.f1.zip › SI Figures/24. Figure S24.jpg]

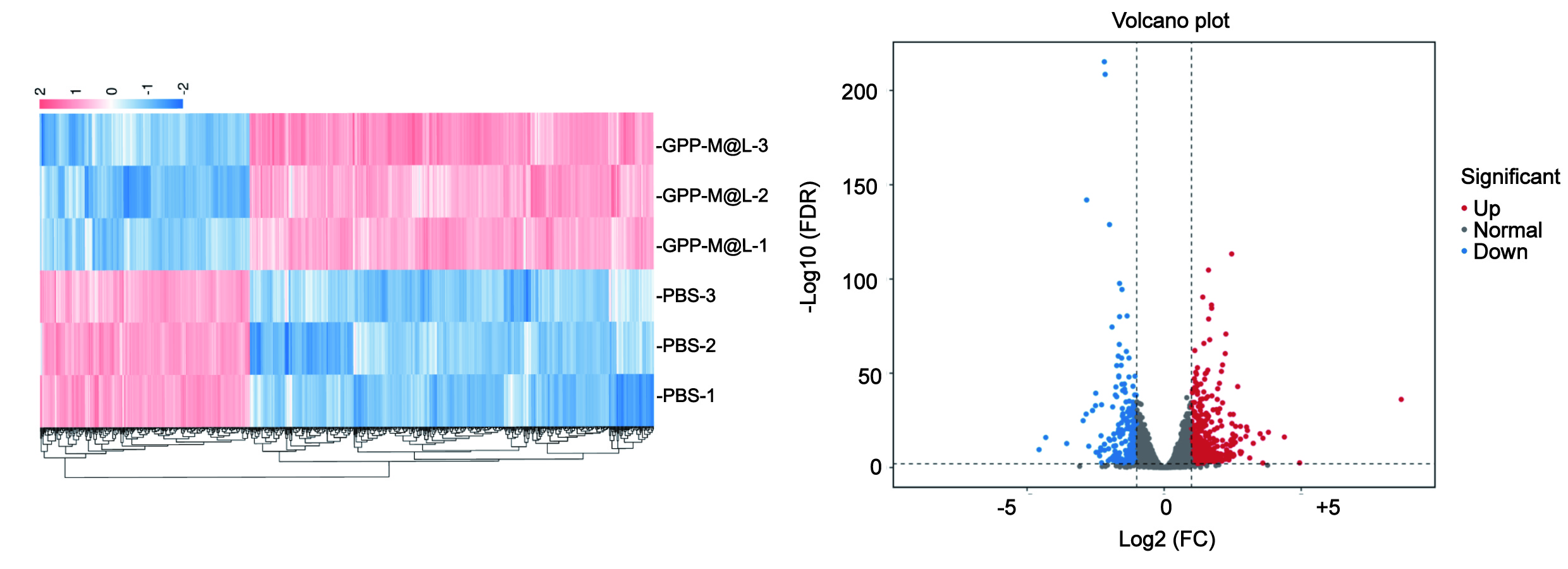

Supplement: Supplementary 1 — Figs. S1 to S34 [file research.0964.f1.zip › SI Figures/25. Figure S25.jpg]

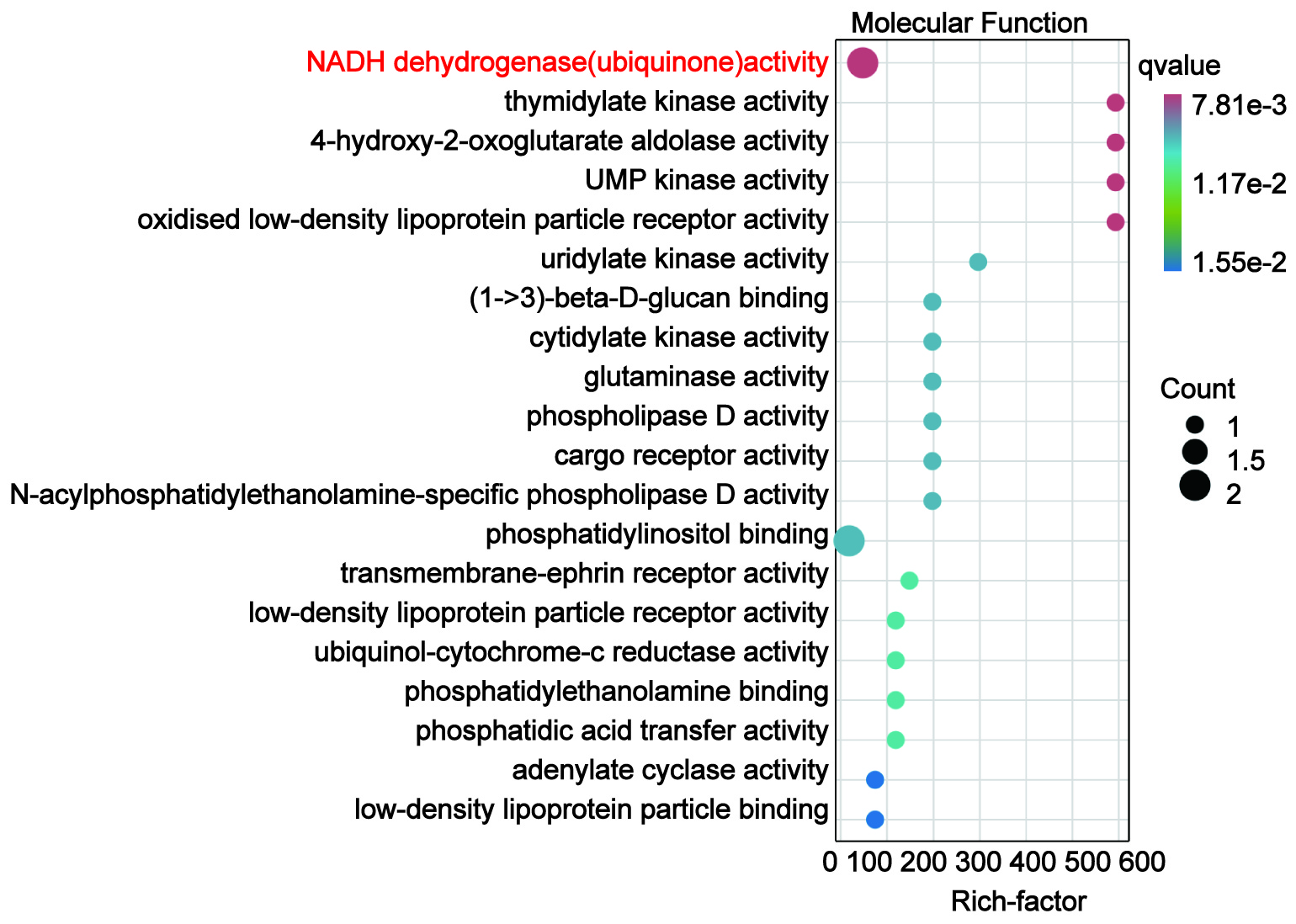

Supplement: Supplementary 1 — Figs. S1 to S34 [file research.0964.f1.zip › SI Figures/26. Figure S26.jpg]

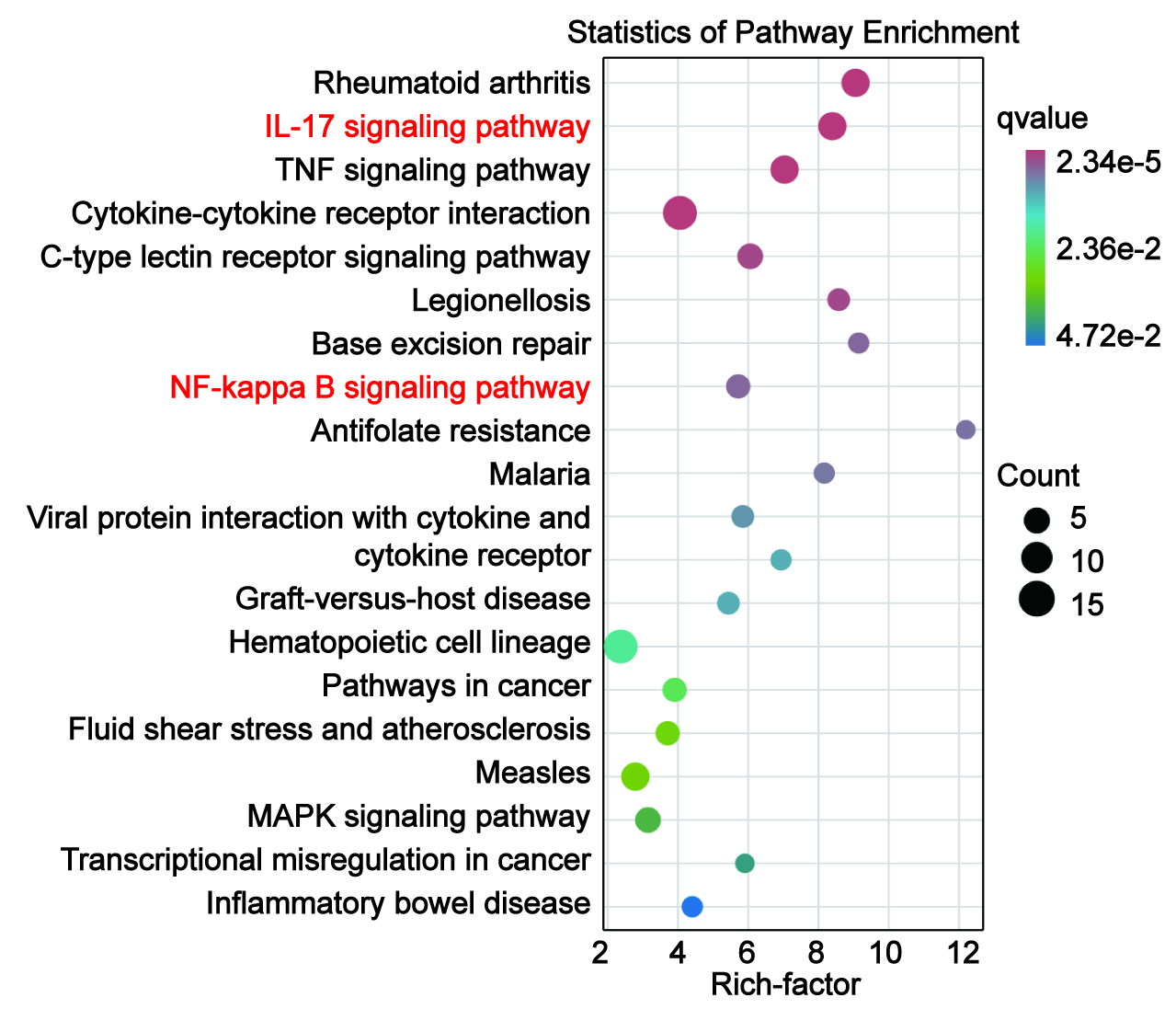

Supplement: Supplementary 1 — Figs. S1 to S34 [file research.0964.f1.zip › SI Figures/27. Figure S27.jpg]

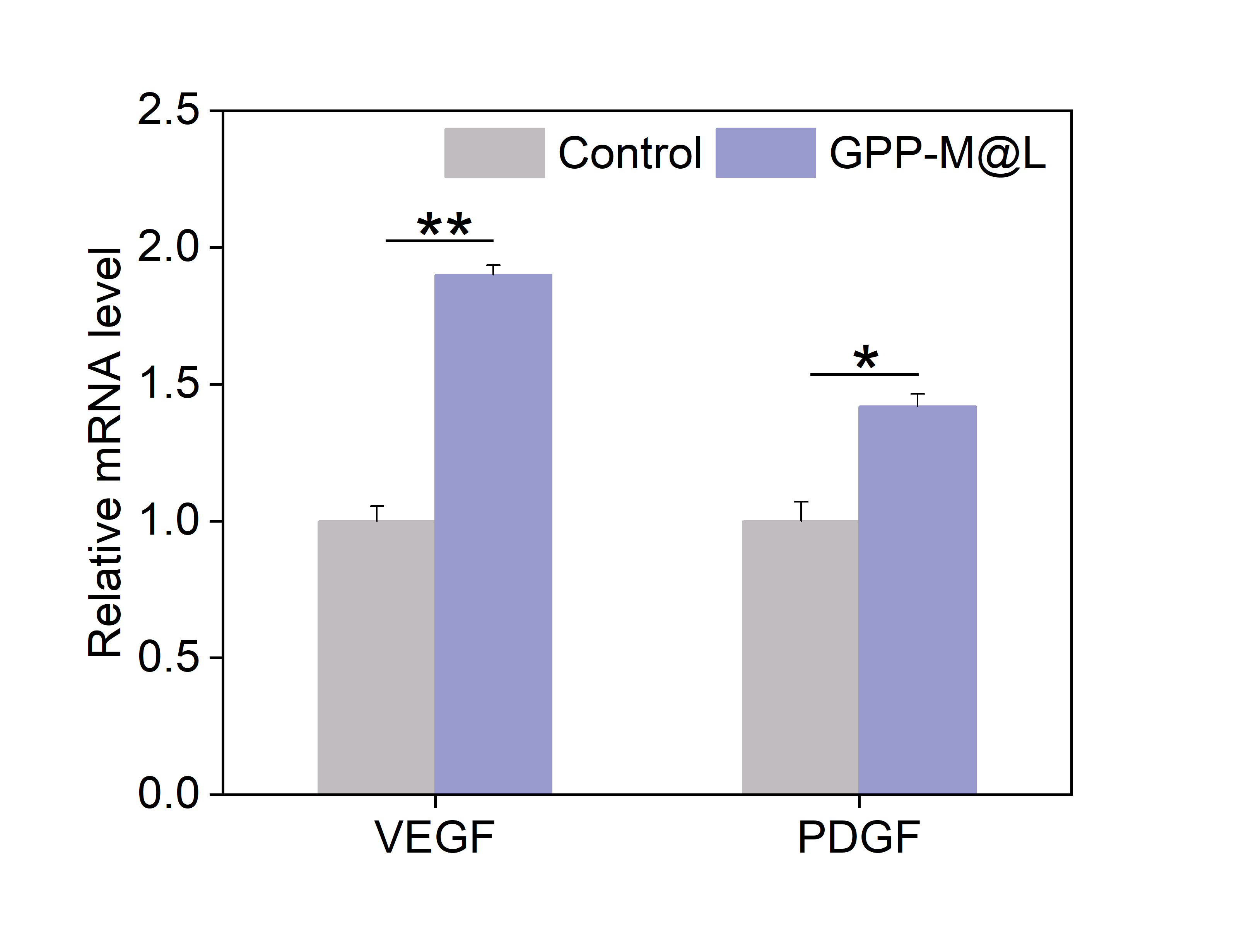

Supplement: Supplementary 1 — Figs. S1 to S34 [file research.0964.f1.zip › SI Figures/28. Figure S28.jpg]

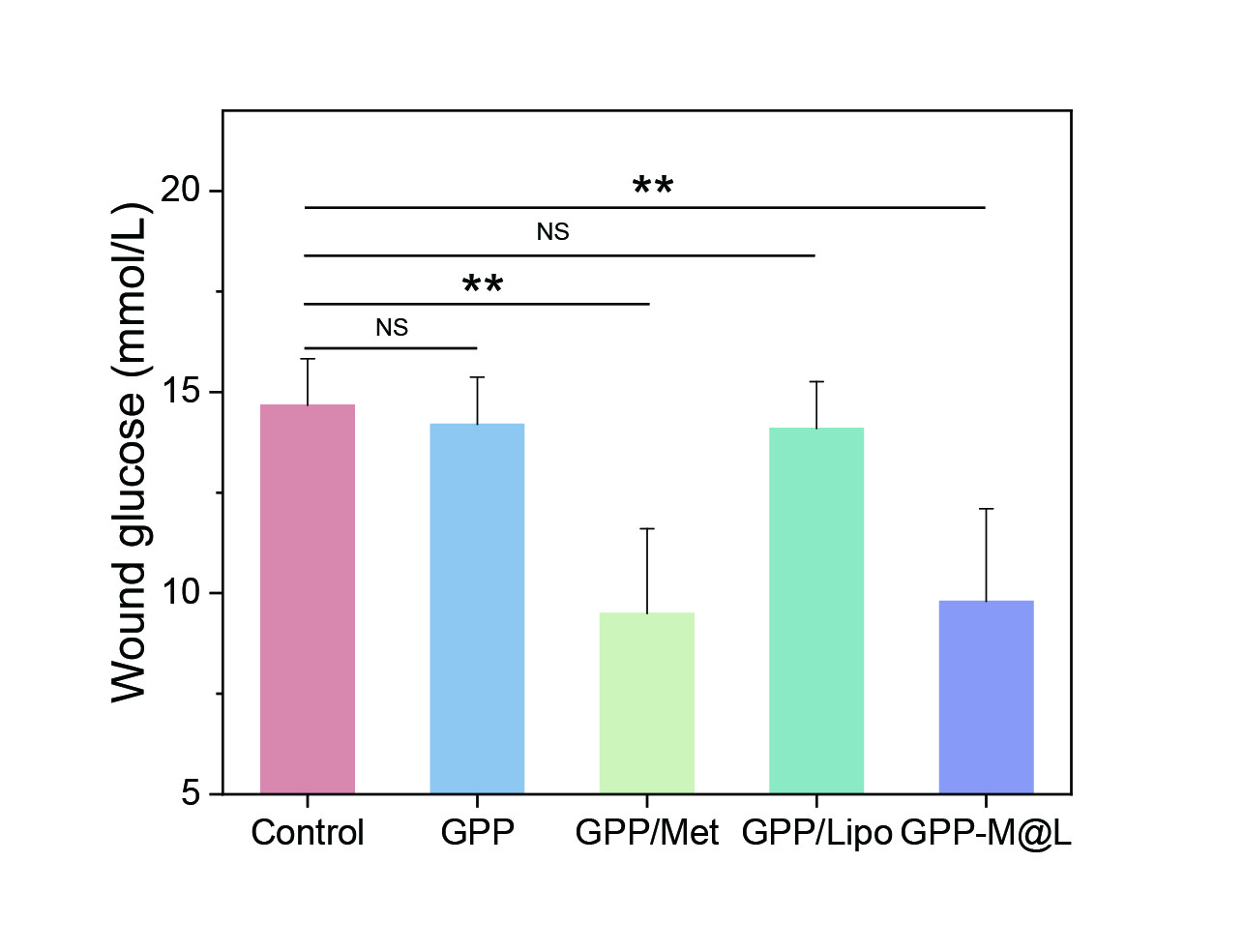

Supplement: Supplementary 1 — Figs. S1 to S34 [file research.0964.f1.zip › SI Figures/29. Figure S29.jpg]

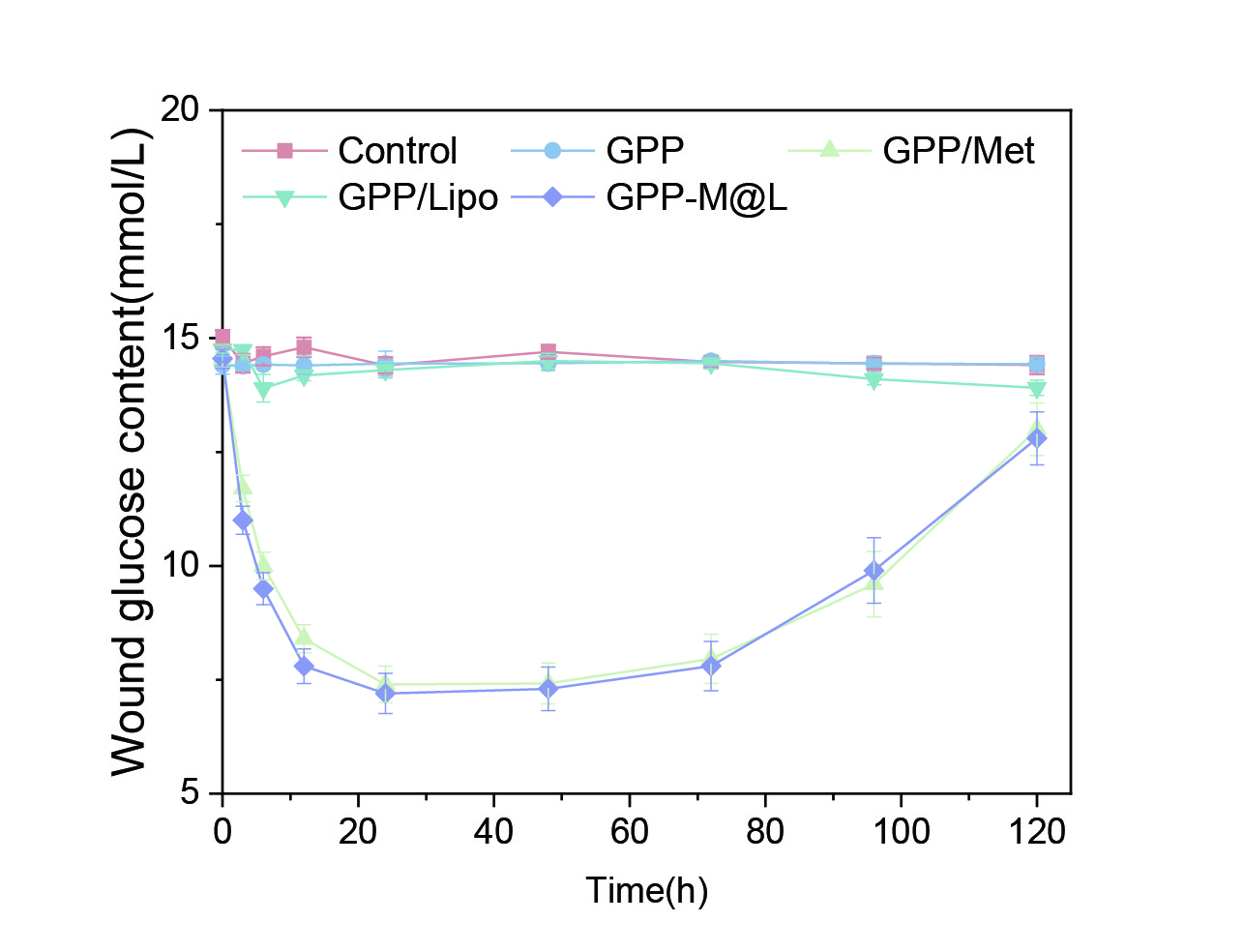

Supplement: Supplementary 1 — Figs. S1 to S34 [file research.0964.f1.zip › SI Figures/30. Figure S30.jpg]

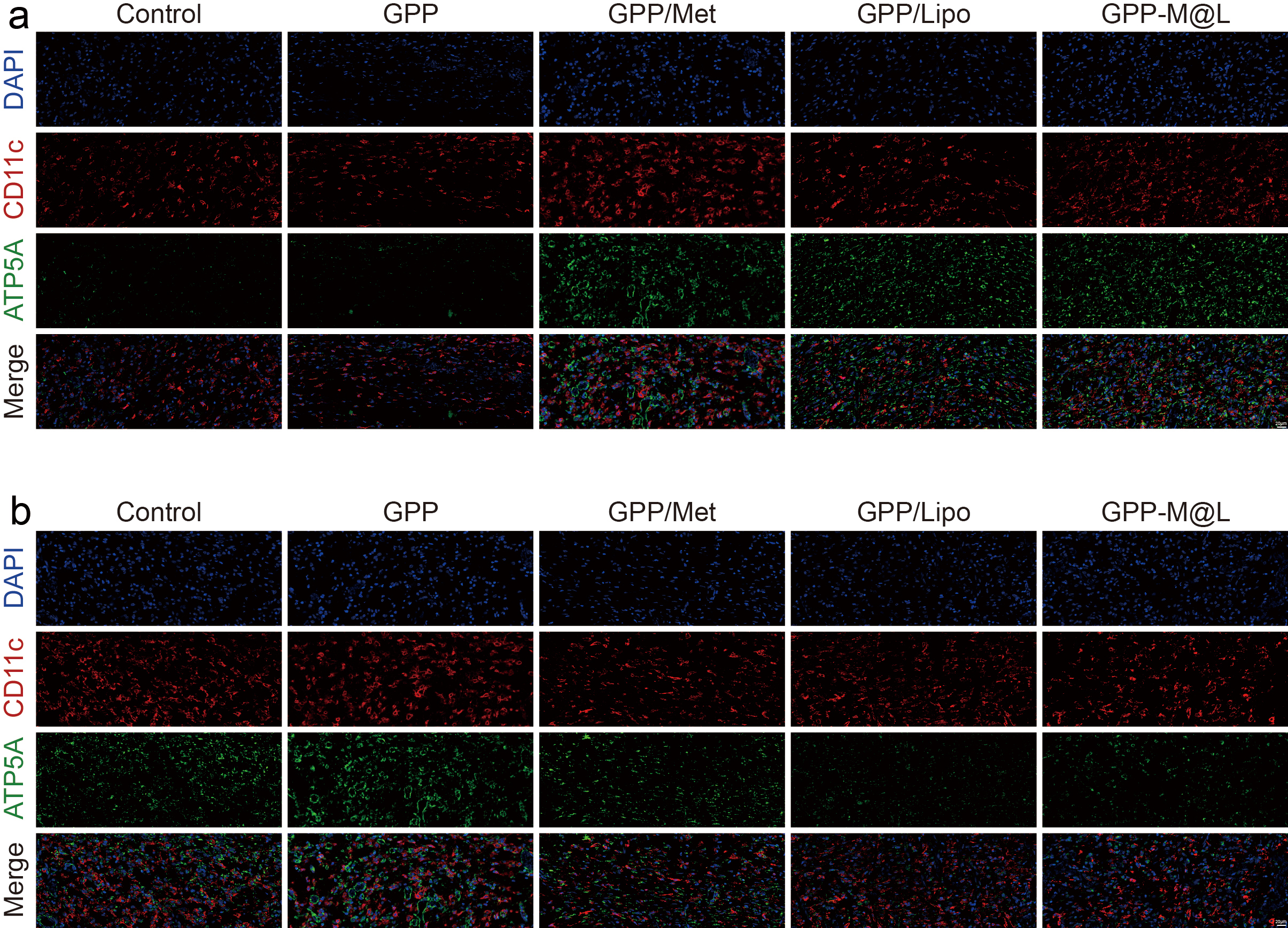

Supplement: Supplementary 1 — Figs. S1 to S34 [file research.0964.f1.zip › SI Figures/31. Figure S31.jpg]

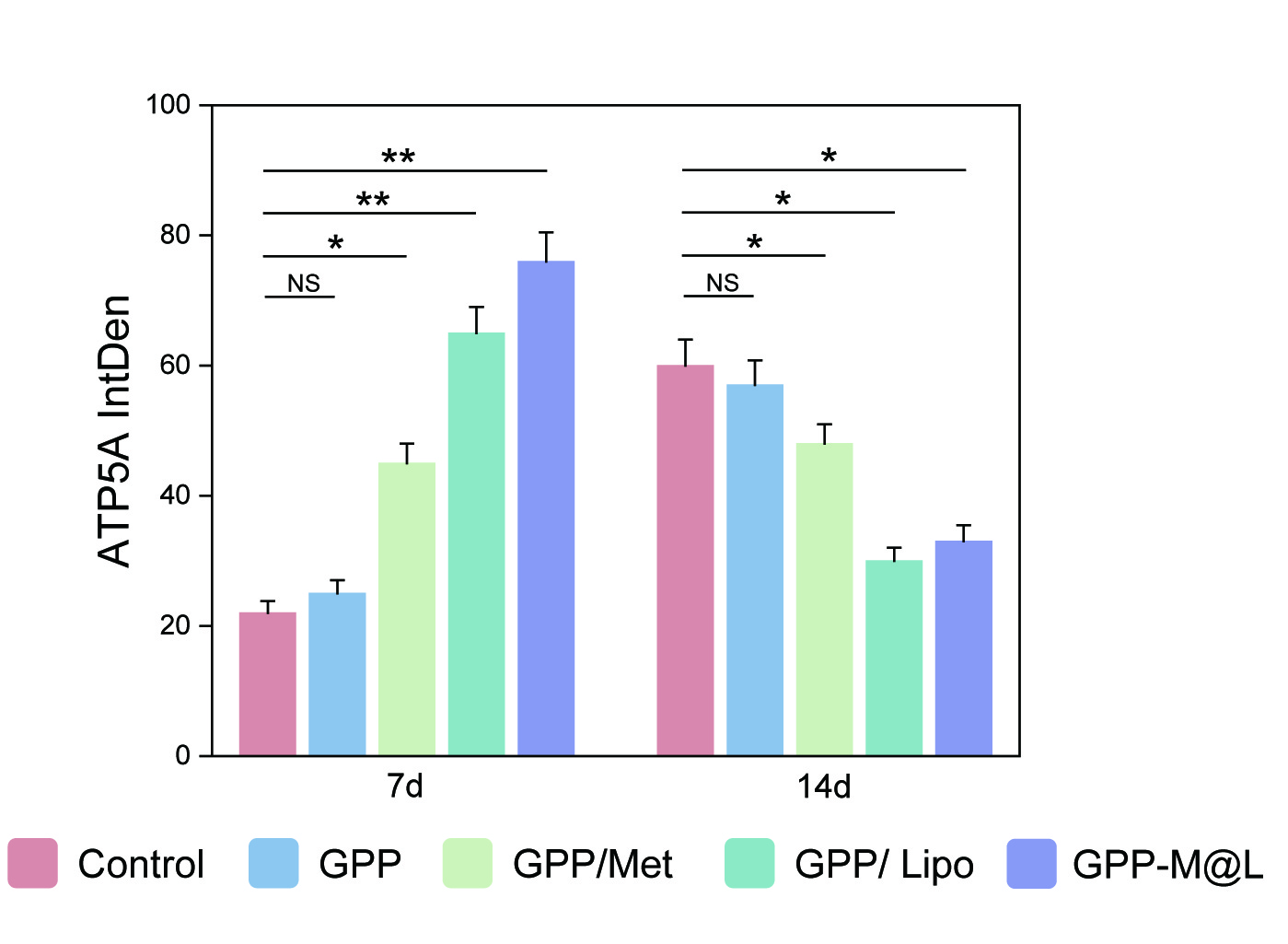

Supplement: Supplementary 1 — Figs. S1 to S34 [file research.0964.f1.zip › SI Figures/32. Figure S32.jpg]

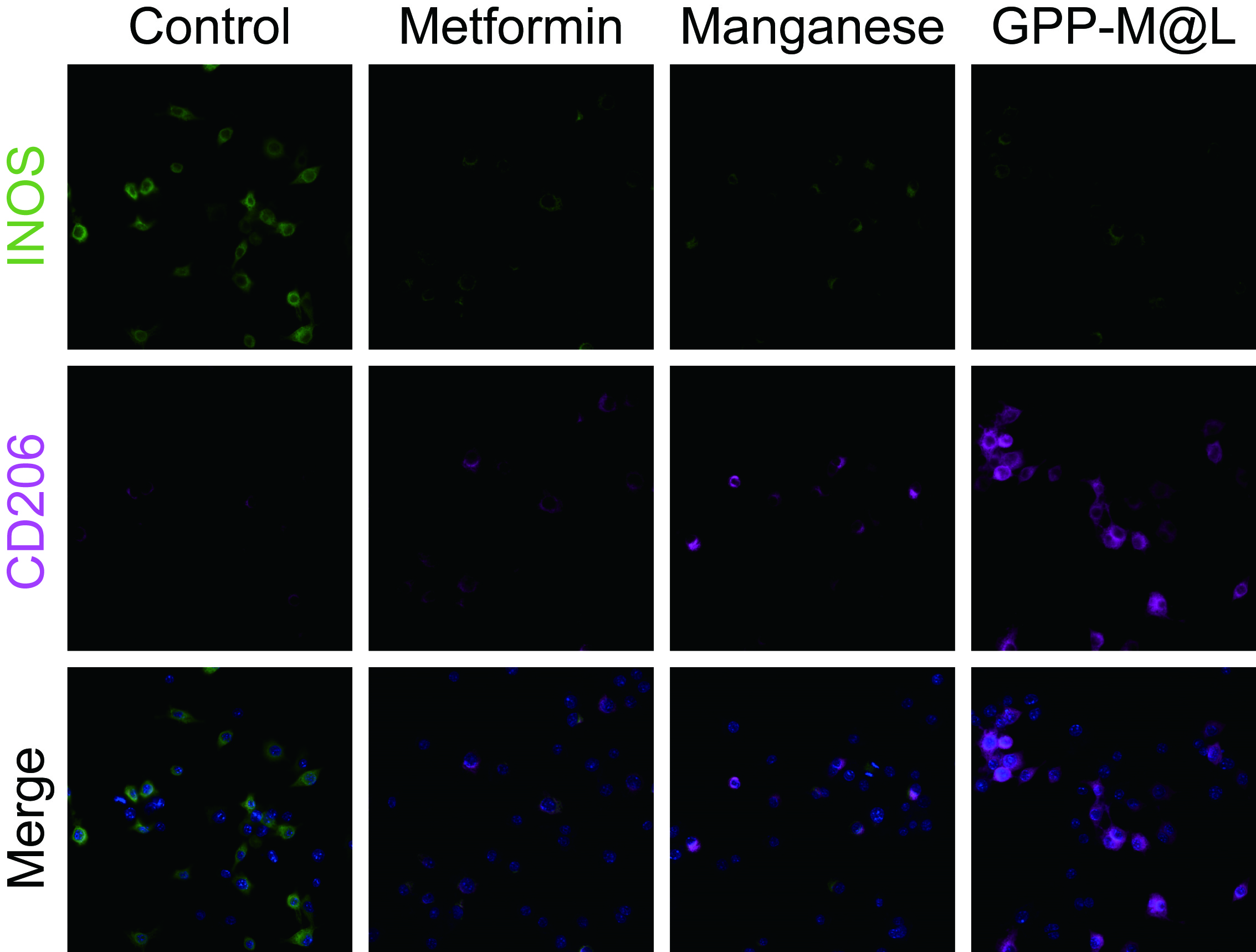

Supplement: Supplementary 1 — Figs. S1 to S34 [file research.0964.f1.zip › SI Figures/33. Figure S33.jpg]

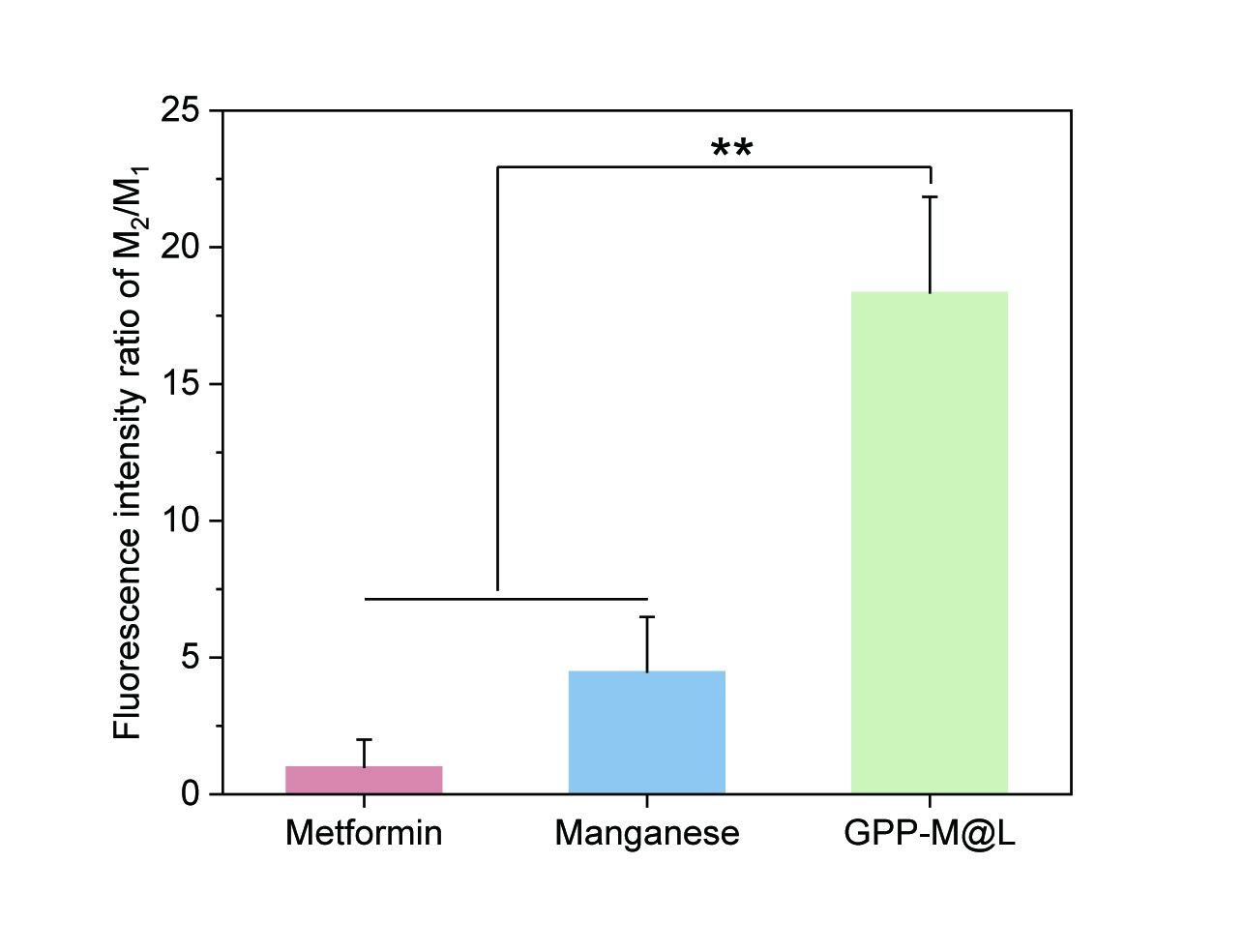

Supplement: Supplementary 1 — Figs. S1 to S34 [file research.0964.f1.zip › SI Figures/34. Figure S34.jpg]
